# Chiral N‑Heterocyclic Carbene On-Surface Induced Electronic Spin Filtration

**DOI:** 10.1021/jacs.6c04854

**Published:** 2026-08-04

**Authors:** Himadri Sekhar Sasmal, Anu Gupta, Arne Nalop, Deb Kumar Bhowmick, Dominik Schwab, Bonnie J. Tyler, Nikos Doltsinis, Ron Naaman, Frank Glorius

**Affiliations:** 1 Organisch-Chemisches Institut, 9185 Universität Münster, Corrensstraße 36, 48149 Münster, Germany; 2 Department of Chemical and Biological Physics, 34976 Weizmann Institute of Science, Rehovot 76100, Israel; 3 Institut für Festkörpertheorie and Center for Multiscale Theory and Computation, Universität Münster, Wilhelm-Klemm-Straße 10, 48149 Münster, Germany; 4 Center for Soft Nanoscience (SoN), University of Münster, Busso-Peus-Strasse 10, 48149 Münster, Germany

## Abstract

It is well established that transport of electrons through chiral molecules can depend on their spins. However, it is not yet comprehensible how to relate molecular structure and size to spin selectivity. N-heterocyclic carbenes (NHCs) are a unique class of small organic molecules with electronic tunability generating huge structural and applicative diversity and covalently bind to metal surfaces to form a robust self-assembled monolayer (SAM). Here, we demonstrate that this family of molecules exhibits high spin selectivity, despite their small size, while being extremely stable on metal surfaces. We have fabricated chiral NHC thin films on the metal surfaces and utilized them as electron spin filters for the first time. The (*S*,*S*)-SINpEt and (*R*,*R*)-SINpEt thin films on Au–Ni surfaces showcase a high electronic spin polarization (SP) of up to 60%. The chiral NHC thin films exhibit electrochemical stability under continuous voltametric interrogation for 50 cycles (within a −0.3 to 0.3 V vs Ag/AgCl voltage window) in an aqueous solution (pH = 13), which enables the utilization of spin-polarized electrons to enhance the oxygen reduction reaction (ORR). The ORR with chiral SINpEt thin films of different thicknesses indicates the importance of the coherency of the electrons in the ORR reaction. The binding, orientation of the chiral SINpEt molecules, and their molecular coverage on the Au surface have also been investigated using quantum chemical calculations, which ensure a good agreement with the experimental observations. This finding introduces a new class of small chiral molecules as a useful source of electronic spin-polarization for various applications.

## Graphical abstract

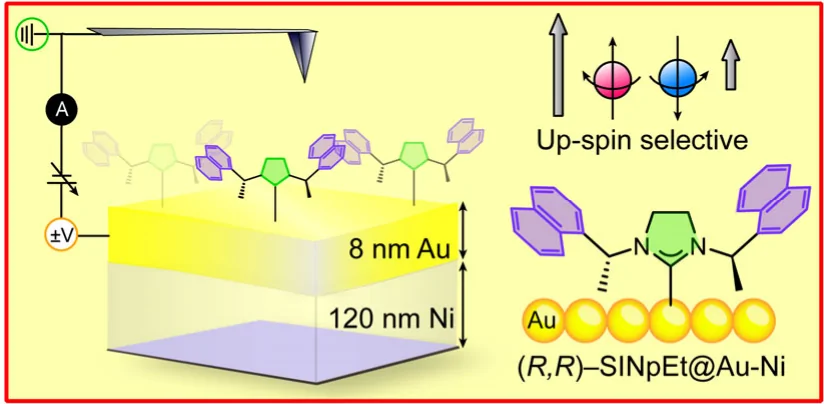

## Introduction

Organic molecules are inferior compared to conventional ferromagnets as spin transport materials due to their weak spin–orbit interaction and low conduction.^1,2^ However, the discovery of the chirality-induced spin selectivity (CISS) effect opened new venues for the development of organic spintronics.^3^ The chiral organic molecules act as spin filters/polarizers and transmit the charge carriers with a preferred spin. Molecular chiral geometry and the spin–orbit coupling (SOC) are the two major parameters controlling the efficiency of the CISS effect.^4^ Larger molecules with heavier atoms possess significant electronic spin–orbital motion interactions and subsequently exhibit stronger SOC compared to small organic molecules with lighter atoms.^5,6^ Indeed, several classes of chiral molecules with large size and higher molecular weight, including biological supramolecules (i.e., DNA, amino acids, and oligopeptides), supramolecular polymers, organic–inorganic hybrid materials, and transition-metal dichalcogenides, have already been investigated as electronic spin filters.^7^

In addition, enantiopure helicenes, overcrowded alkenes, and most recently, bowl-shaped aromatics have been investigated as organic spin filters when absorbed on pyrolytic graphite or various metal surfaces.^2,8,9^ The molecular structure and chirality, orientation relative to the surfaces, the domain, and defects of the self-assembled layer of the molecules significantly influence the overall spin polarization.^10,11^ Conflicting reports over the ability to observe spin polarization in small chiral molecules that are adsorbed on gold substrates raised the question of whether high spin polarization can be obtained with small-sized molecules.^12^

Keeping this in perspective, we introduce chiral NHCs on metal surfaces to form self-assembled thin films and utilize them for efficient spin filtration for the first time. NHCs are a class of organic small molecules that contain a highly active carbon atom stabilized by the “N” heteroatom and its ring structure.^13^ Because of the high structural modularity, tunable electronic and steric properties, and ability to form stable carbon–metal bonds, NHCs have been utilized as robust, versatile, and efficient surface modifiers for various applications.^14–20^ NHCs can form uniform SAMs on surfaces, and their structural orientation can be assessed with various tools like X-ray photoelectron spectroscopy (XPS), sum frequency generation (SFG) spectroscopy, scanning tunneling microscopy (STM), and atomic force microscopy (AFM).^21–30^ The strong electron-donating properties of NHCs and their ability to form stable covalent bonds with transition metals make the SAMs extremely robust on metal surfaces, unlike other organic molecules investigated for the CISS effect. The robustness and exceptional stability of the NHC thin films on Au surfaces surpass the conventional thiol-based modifiers at high temperatures (∼100 °C), under highly acidic or basic (pH range 2–12),^31^ and electrochemical conditions,^32,33^ and establish them as a suitable candidate for various applications like sensing, heterogeneous catalysis, surface protection, and microelectronics.^34–40^ However, the capability of self-assembled layers of chiral NHCs on metal surfaces for electronic spin filtration has not been explored to date.

Hereby, we fabricate self-assembled chiral thin films of NHCs. Subsequently, we investigate the spin filtration ability of the chiral NHC thin film, obtaining high SP values, unheard of for small molecules to date: up to 60% with (*S*,*S*)-SINpEt and (*R*,*R*)-SINpEt. Hence, we clearly demonstrate that the CISS effect can be significant even in the case of small molecules, provided that they are attached to the substrate in a well-defined configuration. We also provide evidence for the role of the film thickness on the efficiency of the ORR reaction when promoted by spin-polarized electrons.

In ORR reactions, the electrochemical potential is influenced by the interaction between the two involved electron pairs and their multiplicity.^41^ Spin-controlled efficiency of the chiral NHC thin films could also be attributed to the coupling between the pair of electrons and thereby influence the ORR mechanism. Additionally, NHC thin film fabrication via chemical vapor deposition also offered control over the thickness of the deposited NHC thin films.

We have performed quantum chemical calculations with the chiral SINpEt molecules on the Au surface to investigate the binding and preferred geometries of the molecules. The thickness of the self-assembled chiral SINpEt thin films and their molecular coverage at different deposition times are well explained and matched with the experimental observations.

## Results and Discussion

In the present study, a 120 nm thick ferromagnetic Ni surface, protected with an 8 nm thin Au layer (Au–Ni), was functionalized with both enantiomers of the chiral NHC [(*S*,*S*)-SINpEt and (*R*,*R*)-SINpEt] using the process of chemical vapor deposition (Figure 1d–f; for additional data, see Supporting Information Section S-2b).

**Figure 1:**
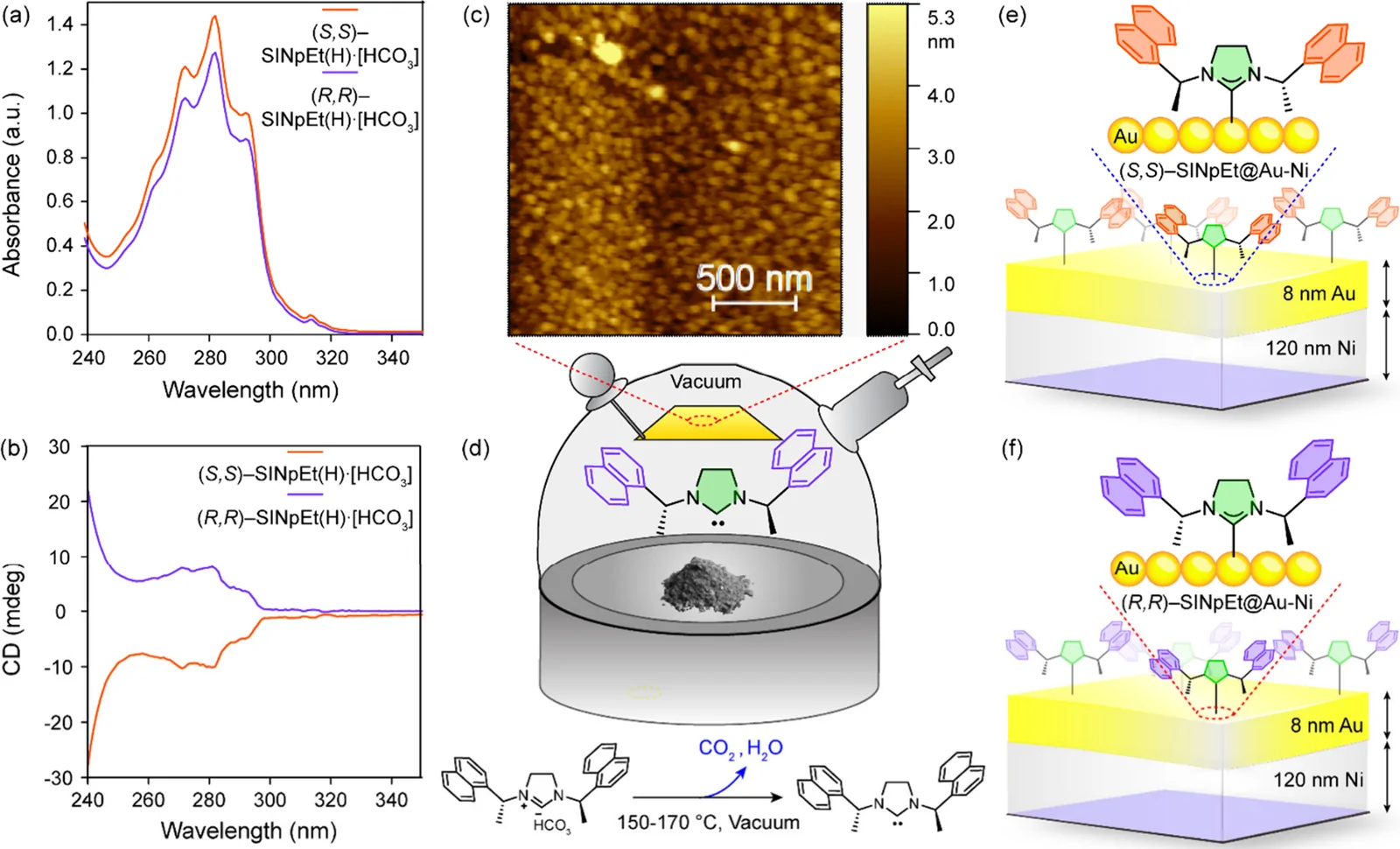
(a) UV–vis and (b) CD spectra of the ∼1 × 10^–5^ M (*S*,*S*)-SINpEt­(H)·[HCO_3_] and (*R*,*R*)-SINpEt­(H)·[HCO_3_], respectively, in methanol. (c) The AFM images of the chiral SINpEt functionalized Au–Ni surface revealed a self-assembled thin film of the SINpEt molecules on the surface. (d) Schematic of the chemical vapor deposition chamber, in which the functionalization of the metal surfaces (8 nm Au–120 nm Ni) with chiral NHCs [(*S,S*)-SINpEt and (*R,R*)-SINpEt] was performed at high temperature (150–170 °C) under vacuum through the generation of free carbenes from the NHC precursors (*S*,*S*)-SINpEt­(H)·[HCO_3_] and (*R*,*R*)-SINpEt­(H)·[HCO_3_]_,_ respectively. (e, f) Schematic of the functionalized Au–Ni surface with (*S,S*)-SINpEt and (*R,R*)-SINpEt, respectively.

### Synthesis of NHC Precursors

Imidazolium hydrogen carbonates (NHC­(H)·[HCO_3_]) are stable NHC precursors that can generate free carbenes by releasing CO_2_ and H_2_O upon heating (40–60 °C).^42,43^ They can generate free carbenes either in solution or in the vapor phase and be successfully fabricated as thin films onto various surfaces for different applications. NHC­(H)·[HCO_3_] salts also have a mild synthetic route and do not include further purification from bases, while anchoring them onto various surfaces.^43^ Several chiral NHCs have already been reported as efficient ligands for asymmetric catalysis.^44^ Glorius and co-workers developed a Ru complex containing 1,3-bis­(1-(naphthalene-1-yl)­ethyl)-4,5-dihydroimidazolylidene (SINpEt) homochiral ligand, which has proven to be a privileged structure with unique activity and selectivity.^45,46^ Keeping these in perspective, we synthesized both (*S*,*S*) and (*R*,*R*) enantiomers of SINpEt­(H)·[HCO_3_] salt and utilized them as active chiral NHC precursors for surface functionalization (Supporting Information Section S-2a).

The enantiomeric pair (*S*,*S*)-SINpEt­(H)·[BF_4_] and (*R*,*R*)-SINpEt­(H)·[BF_4_] was synthesized following the previous literature report.^44,47^ Further, the bicarbonate salts were prepared from their corresponding tetrafluoroborate salts using the anion exchange resin Amberlyst A26-OH.^48^ The recrystallized (*S*,*S*)-SINpEt­(H)·[HCO_3_] and (*R*,*R*)-SINpEt­(H)·[HCO_3_] salts both showcased a broad absorption band in the range of 260–315 nm in the UV–vis spectra (Figure 1a, Supporting Information Section S-3). The circular dichroism (CD) spectra of the 10 μM (*S*,*S*)-SINpEt­(H)·[HCO_3_] and (*R*,*R*)-SINpEt­(H)·[HCO_3_] enantiomers were measured in methanol. As expected, the CD spectra of the respective (*S*,*S*) and (*R*,*R*) enantiomers were mirror images of each other (Figure 1b, Supporting Information Section S-3).

### NHC Thin Films on the Surface

The utmost efficiency in filtering the electrons based on their spin demands a high coverage, densely packed, impurity-free, and defect-free assembly of chiral NHC molecules on surfaces. Initially, we tried to functionalize the ferromagnetic 120 nm thick Ni surface (protected with an 8 nm thin Au layer) with chiral NHCs by generating the free carbenes from the corresponding NHC­(H)·[HCO_3_] salts in solution (Supporting Information Section S-2b). For this purpose, cleaned (with hot ethanol followed by dry acetone) and dried (under argon flow) Au–Ni surfaces were placed inside 5 mL of a 2 mM solution of (*S*,*S*)-SINpEt­(H)·[HCO_3_] in methanol. The surfaces were kept within the solution for 2 h at 40 °C. After the adsorption of NHCs, the Au–Ni surfaces were removed from solution and thoroughly washed with HPLC-grade methanol, followed by HPLC-grade isopropanol and acetone. After drying under an argon flow, the (*S*,*S*)-SINpEt functionalized surfaces were characterized with time-of-flight secondary ion mass spectrometry (ToF−SIMS) and analyzed for their efficiency in electronic spin filtration. A similar procedure was followed for the functionalization of Au–Ni surfaces with (*R*,*R*)-SINpEt molecules.

However, the ToF−SIMS mapping images (Figure S29, Supporting Information Section S-6) of both the surfaces [(*S*,*S*)-SINpEt@Au–Ni and (*R*,*R*)-SINpEt@Au–Ni] revealed the uneven distribution of NHCs on surfaces with inconsistent thicknesses. Consequently, the electronic spin polarization measurement and the *I*–*V* curves recorded by mc-AFM of the chiral NHC-functionalized surfaces were poor and inconsistent throughout the entire surface. In a previously reported work by the Glorius group and co-workers, vibrational sum-frequency generation spectroscopy analysis of the NHC thin film on the Au surface revealed the dynamic nature of the NHC-metal binding mode in solution.^49^ This dynamic change of the binding mode during the formation of the NHC thin film in solution could lead to the inconsistent molecular coverage of the deposited thin film. As an alternative to the previous method, the (*S*,*S*)-SINpEt­(H)·[BF_4_] and (*R*,*R*)-SINpEt­(H)·[BF_4_] salts were treated separately with KO*t*Bu in toluene to generate the corresponding free carbenes. After purification of the generated free carbenes through filtration under argon, they were used for the functionalization of the Au–Ni surface. A similar observation to the previous NHC­(H)·[HCO_3_]–methanol (in solution functionalization from NHC­(H)·[HCO_3_] salt, Supporting Information Section S-2b) method was also observed when the isolated and purified surfaces [(*S*,*S*)-SINpEt@Au–Ni and (*R*,*R*)-SINpEt@Au–Ni] were analyzed and tested for spin-dependent conductivity. These observations demanded different surface functionalization methods to achieve defect-free NHC thin films with consistent molecular coverage and fewer grain boundaries.

In adaptation to the vapor deposition method used for STM analysis, an evaporation chamber was designed and utilized for the fabrication of NHC thin films on surfaces (Figure 1d and Supporting Information Section S-2b). The (*S*,*S*)-SINpEt­(H)·[HCO_3_] and (*R*,*R*)-SINpEt­(H)·[HCO_3_] salts were evaporated at ∼150–170 °C under high vacuum (10^–1^ to 10^–2^ mbar) in the presence of the Au–Ni surfaces within the evaporation chamber for 30 min. This resulted in the generation of free carbenes via the release of CO_2_ and H_2_O and subsequent deposition of the chiral NHC molecules on Au–Ni surfaces through the NHC–metal covalent bond formation. After cooling down the evaporation chamber to room temperature, the functionalized Au–Ni surfaces were treated with isopropanol and dry acetone to remove physisorbed imidazolium salts on the Au–Ni surfaces. Further, the [(*S*,*S*)-SINpEt@Au–Ni and (*R*,*R*)-SINpEt@Au–Ni] surfaces were dried under an argon flow and characterized with XPS, AFM, and ToF-SIMS before analyzing their electron spin filtration ability. We also fabricated (*S*,*S*)-SINpEt@Au and (*R*,*R*)-SINpEt@Au surfaces using the evaporation chamber, where (*S*,*S*)-SINpEt and (*R*,*R*)-SINpEt were deposited on an Au surface under similar conditions.

### Characterization of the NHC Thin Films

The self-assembled thin films of the chiral NHCs on Au–Ni surfaces were analyzed through XPS. The adsorption of (*S*,*S*)-SINpEt and (*R*,*R*)-SINpEt on the Au–Ni surface was verified by recording the N 1s X-ray photoelectron spectra (XPS) (Figure 2c,d, Supporting Information Section S-4). N 1s XP spectra of both the (*S*,*S*)-SINpEt@Au–Ni and (*R*,*R*)-SINpEt@Au–Ni surfaces revealed an intense single peak with the maximum at 400.6 and 400.4 eV binding energy, respectively, which is in good agreement with the reported binding energy of the surface-bound NHC.^51^ The integrated Au 4f and N 1s spectra of both (*S*,*S*)-SINpEt@Au–Ni and (*R*,*R*)-SINpEt@Au–Ni surfaces indicated high monolayer coverages by the SINpEt molecules (Figure 2a,b, Supporting Information Section S-5).^52^ The electrochemical stability of the NHC thin films on Au–Ni surfaces was also investigated using XPS. After continuous voltametric interrogation for 50 cycles in 0.1 M KOH aqueous solution (pH = 13), using (*S*,*S*)-SINpEt@Au–Ni and (*R*,*R*)-SINpEt@Au–Ni as working electrodes within the −0.3 to 0.3 V vs Ag/AgCl voltage window (Supporting Information Section S-8), the XPS spectra of the surfaces were recorded (Figure 2c,d, Supporting Information Section S-8). The N 1s XPS spectra of the electrochemically treated surfaces reveal the presence of chemisorbed NHC molecules on the Au surface. However, we noticed a slight broadening of the N 1s XP spectra of both surfaces, with a decrease in the molecular coverage. We ascribe this observation to the reorientation of the adsorbed NHCs on the Au–Ni surface under the electrochemical conditions. The AFM was used to investigate the morphology of the NHC-functionalized Au surfaces. The AFM imaging of the NHC functionalized Au surface showcased the presence of consistent self-assembled thin films (Figure 1c). Further, the AFM scratching method was employed on (*S*,*S*)-SINpEt@Au and (*R*,*R*)-SINpEt@Au surfaces using the contact mode with increased normal force to determine the thickness of the NHC thin films. The thickness analysis revealed that the thickness of the (*S*,*S*)-SINpEt and (*R*,*R*)-SINpEt thin films on the Au surface is ∼1.29 nm and ∼1.08 nm, respectively (Figure 3e,i). The functionalization of Au–Ni surfaces with (*S*,*S*)-SINpEt and (*R*,*R*)-SINpEt molecules was confirmed by the ToF–SIMS measurement.^53^ The highly intense peak at 379.23 *m*/*z* (C_27_H_27_N_2_^+^) and the relatively less intense peak at 575.20 *m*/*z* (C_27_H_27_AuN_2_^+^) observed in the ToF–SIMS spectra confirmed the presence of covalently bound SINpEt molecules on both the (*S*,*S*)-SINpEt@Au–Ni and (*R*,*R*)-SINpEt@Au–Ni surfaces (Figure 2e,f). The pseudomolecular ion (NHC + H^+^) at 379.23 *m*/*z* was formed during the ionization process during ToF-SIMS measurement. The presence of this pseudomolecular ion even after the thorough washing procedure (with isopropanol followed by dry acetone) after surface functionalization indicated a strong interaction between NHC and the surface beyond adsorption. The peak at 575.20 *m*/*z* confirmed the covalent binding of the SINpEt carbenes to the metal via a covalent bond. The visualization of ToF-SIMS distribution mapping of both the ions at 379.23 *m*/*z* (C_27_H_27_N_2_^+^) and 575.20 *m*/*z* (C_27_H_27_AuN_2_^+^) disclosed the uniform spatial distribution of the SINpEt carbene bound to the Au atom throughout the entire Au–Ni surface (Supporting Information Section S-6). Contact angle measurement with a water droplet was also carried out for the bare Au, (*S*,*S*)-SINpEt@Au, and (*R*,*R*)-SINpEt@Au surfaces to verify the changes in the wettability of the Au surfaces after functionalization (Supporting Information Section S-7). No significant change in the contact angle (∼76°) of the functionalized Au surfaces was observed compared to the bare/pristine Au surface (∼72.1°). Consequently, the functionalization of the Au surface with (*S*,*S*)-SINpEt and (*R*,*R*)-SINpEt molecules did not interrupt the interaction between the electrolyte and the electrode’s surface in water for efficient electron transfer activity.^54^

**Figure 2:**
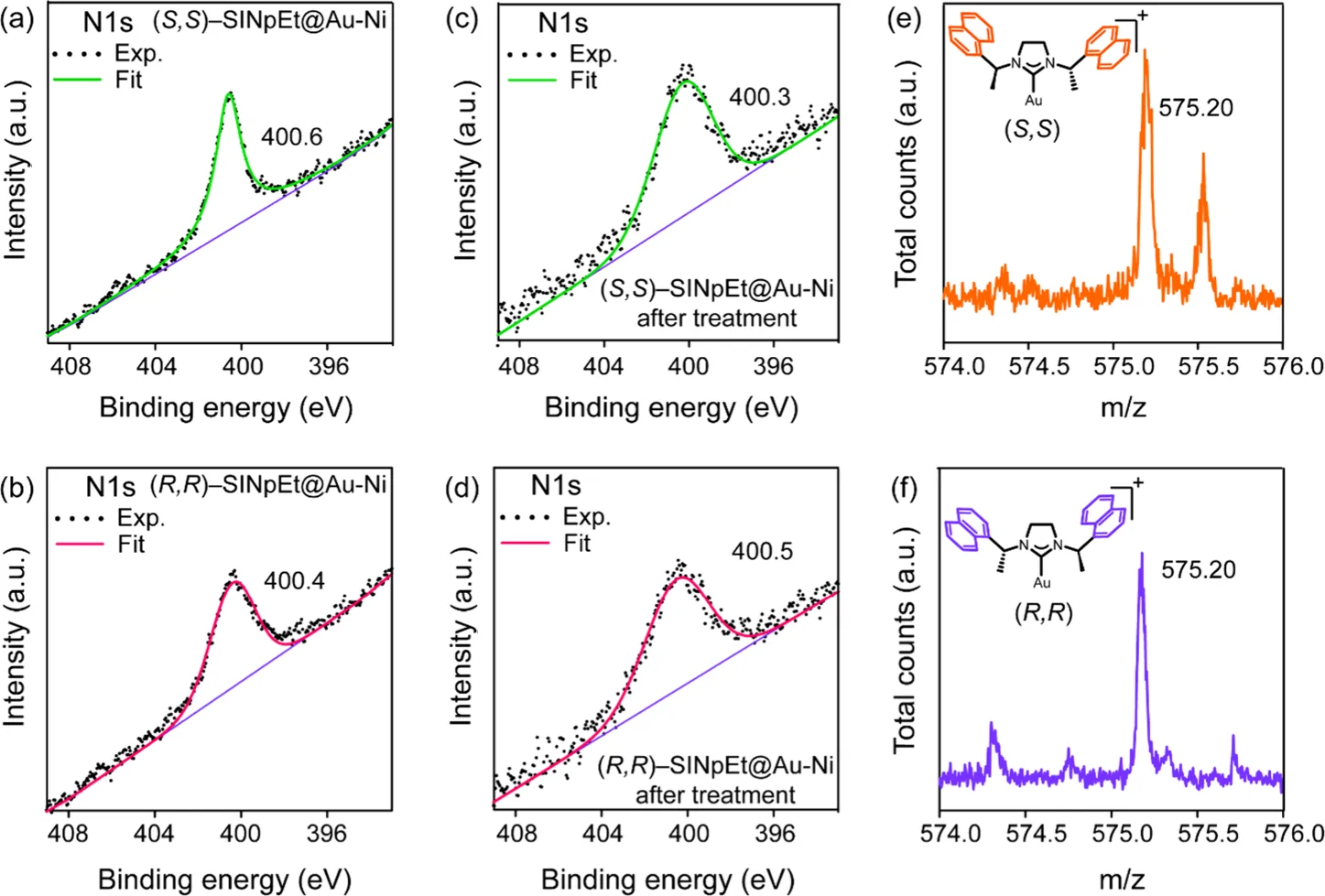
(a, b) XP N 1s spectra obtained from the (*S*,*S*)-SINpEt and (*R*,*R*)-SINpEt functionalized Au–Ni surfaces, respectively. Here, the deposition time was maintained at 30 min during chemical vapor deposition for both enantiomers. (c, d) XP N 1s spectra obtained from the (*S*,*S*)-SINpEt and (*R*,*R*)-SINpEt functionalized Au–Ni surfaces after the continuous voltametric interrogation for 50 cycles in 0.1 M KOH aqueous solution (pH = 13) within the −0.3 to 0.3 V vs Ag/AgCl voltage window, respectively. (e, f) ToF-SIMS spectra obtained from the (*S*,*S*)-SINpEt@Au–Ni and (*R*,*R*)-SINpEt@Au–Ni surfaces, respectively.

**Figure 3:**
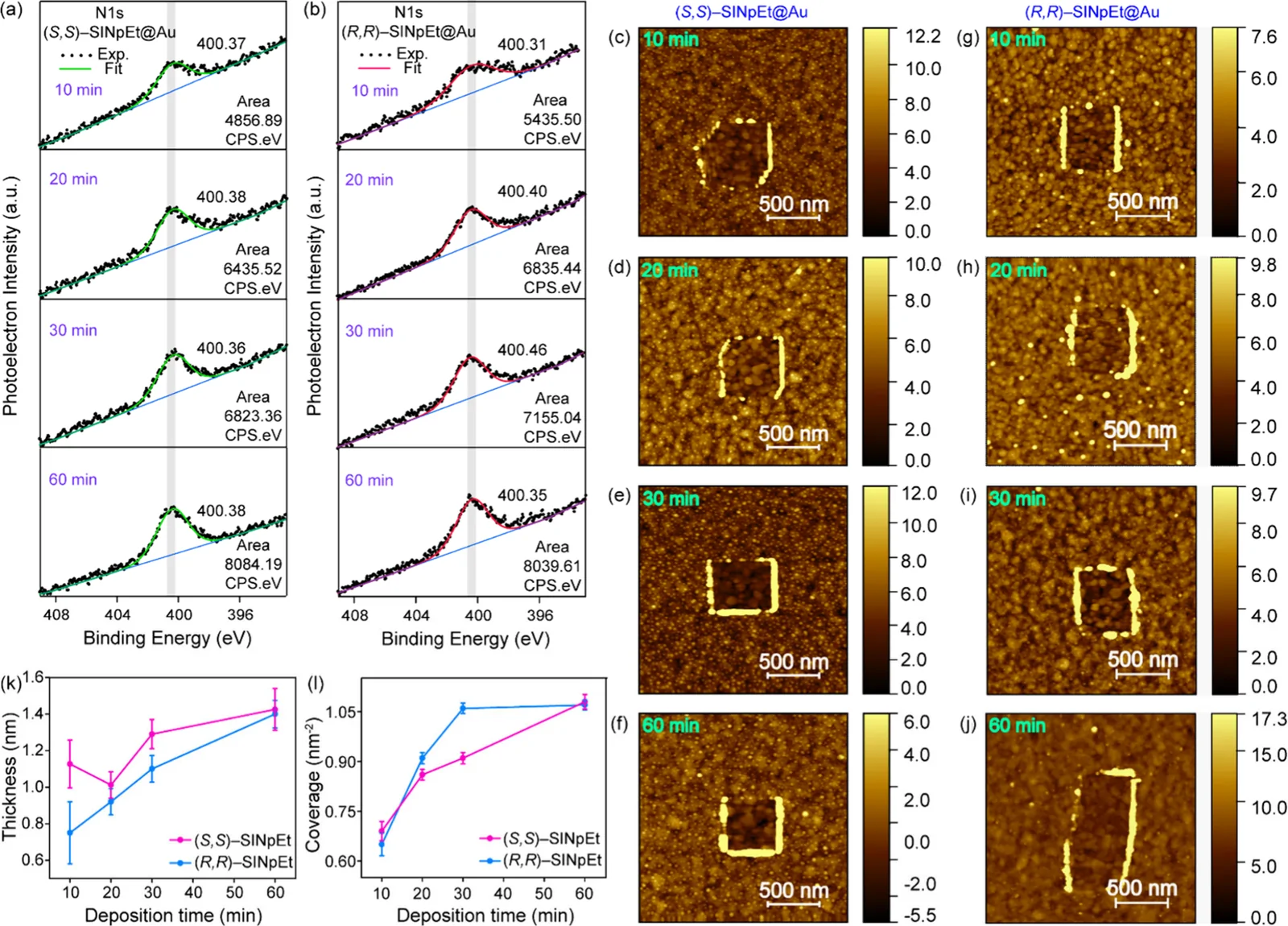
(a, b) The series of XP N 1s spectra obtained from the (*S,S*)-SINpEt and (*R,R*)-SINpEt functionalized Au surface with different deposition times (10, 20, 30, and 60 min), respectively, utilized in the CVD method of surface functionalization with NHCs. (c–f) The AFM images of the (*S*,*S*)-SINpEt thin films and (g–i) (*R*,*R*)-SINpEt thin films on the Au surface, varying with different deposition times (10, 20, 30, and 60 min). AFM images were collected immediately after high-force contact scanning of the NHC functionalized Au surfaces, respectively. The color scale bars on the right indicate the height values in nm. Forces were selected to be sufficient to remove the SINpEt molecules from the surface but small enough to prevent scratching the gold substrate, resulting in film thicknesses of ∼0.75, 0.91, 1.08, and 1.40 nm, respectively.^50^ (k) The thickness of the (*S,S*)-SINpEt and (*R,R*)-SINpEt layers with different deposition times. (l) The coverage of the (*S,S*)-SINpEt and (*R,R*)-SINpEt molecules (number of the SINpEt molecules per nm^2^ surface area) on the Au surface at different deposition times.

### Spin-Polarized Conductive AFM Measurements

The magneto resistance measurements with thin films of (*S*,*S*)-SINpEt and (*R*,*R*)-SINpEt were conducted using magnetic conductive atomic force microscopy (mcAFM) (Figure 4b,c, Supporting Information Section S-10). A scheme representing the mc-AFM setup is shown in Figure 4a. Within some approximations, this established method was demonstrated to be effective for local spin-dependent conductivity measurements across adsorbed molecular layers. In the current studies, the nonmagnetic AFM tip was grounded, and the potential on the conductive Au–Ni surface was varied when a magnet was used to magnetize the Ni substrate with its north pole pointing toward the layer (up) or away from the layer (down). As a result, the spins of the conducted electrons were aligned either parallel or antiparallel to the direction of the current. A magnetic field of 0.5 T was applied using a pair of electromagnets, with the AFM setup positioned between them. The applied magnetic field was large enough to fully saturate the magnetic moment of Ni. The *I*–*V* curves shown in Figure 4b,c were the result of the average of ∼70 *I*–*V* curves acquired on the thin films obtained with the two enantiomers. Both enantiomers consistently exhibited magnetization-direction-dependent current transport with respect to the substrate magnetization orientation. The average *I*–*V* curves exhibited two separate current onsets depending on the direction of the applied magnetic field; each was associated with different spin polarization values (Figure 4d). When the (*R*,*R*)-SINpEt thin film was measured, a higher current was obtained with magnetic fields pointing away from the molecular monolayer, while the opposite behavior was observed with (*S*,*S*)-SINpEt. The applied magnetic field modulated the spin selectivity of the (*S,S*) enantiomer in a manner opposite to that observed for the (*R,R*) enantiomer, highlighting the crucial role of molecular chirality in spin-dependent transport. The observed behavior was in excellent accordance with previous reports,^9^ which showed that spin selectivity changes sign as a consequence of chirality inversion. The variation in threshold voltage for the injection of the two spins indicated that no spin flipping takes place during electron transmission via (*S*,*S*)-SINpEt and (*R*,*R*)-SINpEt thin films. The spin polarization, SP%, is defined as SP(%) = [(*I*_up_ *– I*_dn_)/(*I*_up_ + *I*_dn_)] × 100, where *I*_up_ and *I*_dn_ denote the current when the north pole of the magnetic field is oriented upward or downward, respectively. Since the measurements are performed by measuring charge current in the presence of a magnetic field, the SP is actually related to the magnetoresistance of the system. The dependence of the SP on the applied voltage (Figure 4d) indicated SP values for (*S*,*S*)-SINpEt and (*R*,*R*)-SINpEt of −62 ± 5% and 57 ± 6%, respectively. These values were similar to those obtained from self-assembled monolayers of oligopeptides, dsDNA,^55^ and helicenes.^8^ Notably, the thin films of chiral carbenes exhibited significantly higher spin polarization values than conventional self-assembled monolayers of small molecules, for which SP values are typically lower than 40%.^8^

**Figure 4:**
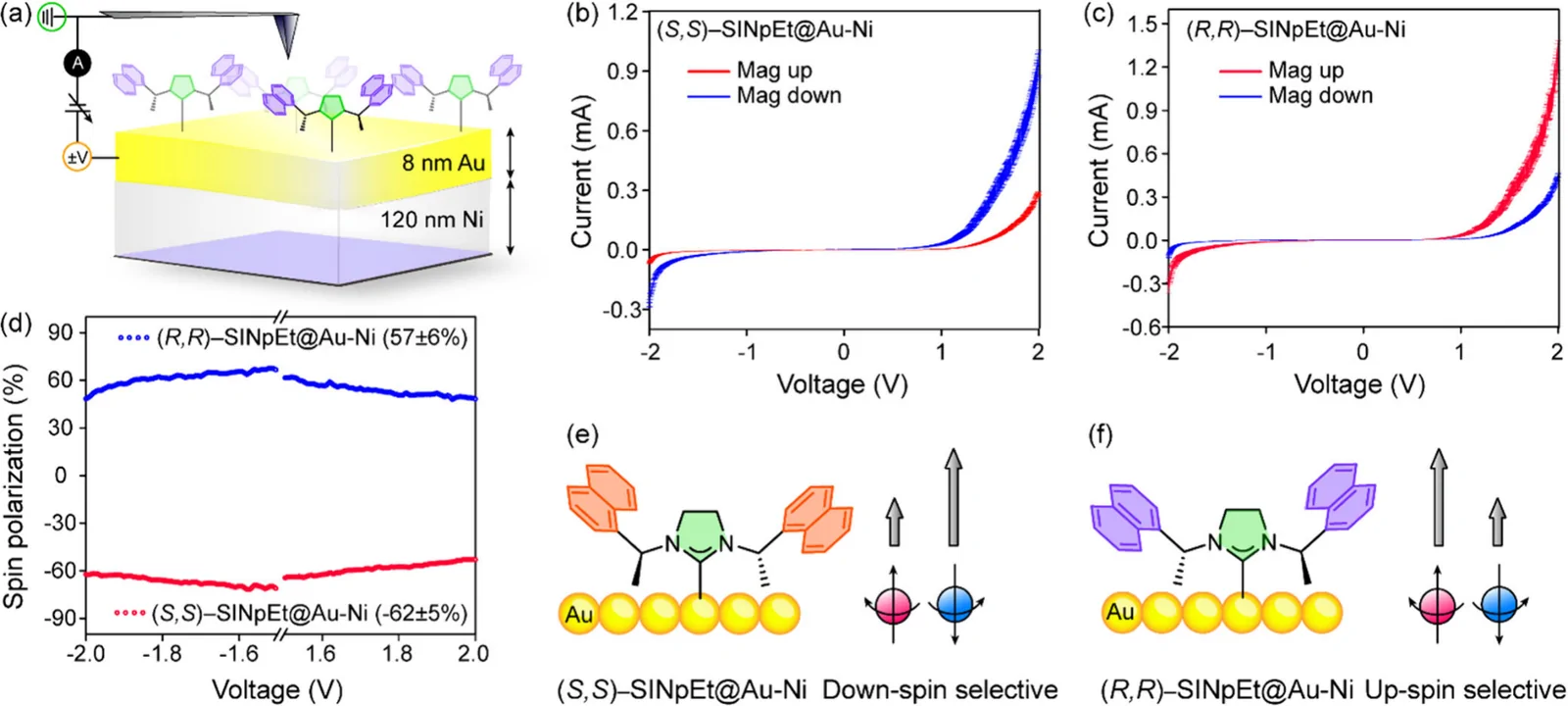
(a) The schematic of the mcAFM measurement of the SINpEt functionalized Au–Ni surfaces. (b, c) The current versus voltage (*I*–*V*) curves measured for the (*S,S*)-SINpEt and (*R,R*)-SINpEt functionalized Au–Ni surfaces. (d) Percentage of spin polarization of (*S,S*)-SINpEt and (*R,R*)-SINpEt functionalized Au–Ni surfaces. (e, f) The spin-selectivity measurement showcased down-spin and up-spin selectivity in electron transmission by the (*S,S*)-SINpEt and (*R,R*)-SINpEt molecules, respectively.

### Enantioselective Interaction between Chiral Ferrocene and Chiral NHC

To further confirm the chirality of the deposited NHC thin films and their enantioselective interaction with chiral molecules, electrochemical experiments utilizing chiral ferrocene were conducted. A direct demonstration that the NHC thin film on the surface is chiral is presented in Figure 5a,b. The cyclic voltammetry (CV) curves were recorded for nonmagnetic electrodes (Au) coated with either (*S*,*S*)-SINpEt and (*R*,*R*)-SINpEt thin films in the presence of a solution containing either the (*R*) or (*S*) chiral *N*,*N*-dimethyl-1-ferrocenyl-ethylamine (Ugi’s amine). A significant enantioselective interaction was observed. The (*S*,*S*)-SINpEt film exhibits a stronger redox peak with (*R*)-Ugi’s amine (blue curve), whereas (*R*,*R*)-SINpEt film shows higher current with (*S*)-Ugi’s amine (pink curve) (Figure 5a,b). The additional scans of the cycles are shown in Figure S34 (Supporting Information Section S-9). To further confirm the reproducibility of enantioselective interaction, additional data from two independent surfaces are shown in Figure S35 (Supporting Information Section S-9).

**Figure 5:**
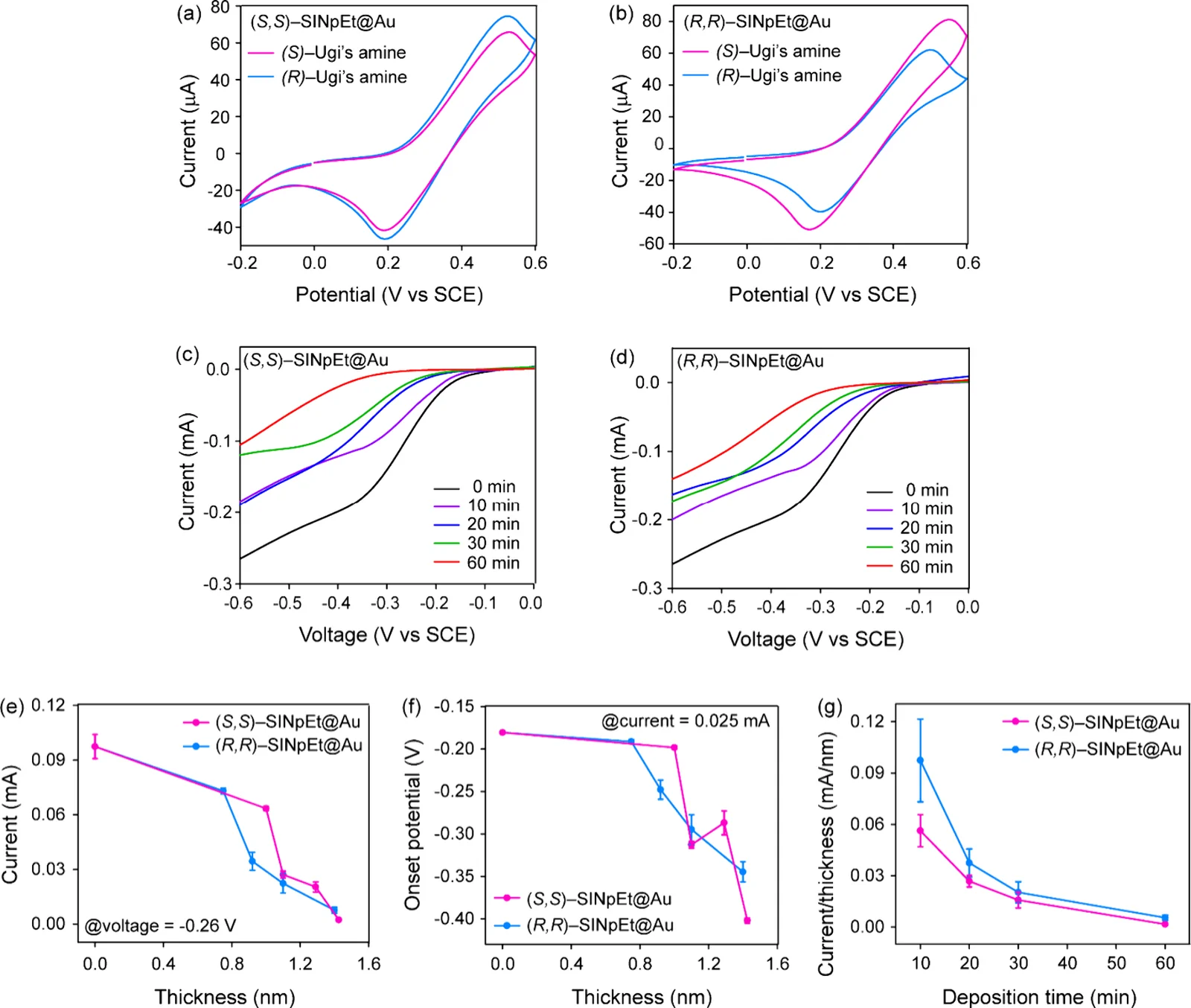
(a, b) Spin-dependent CV of chiral *N*,*N*-dimethyl-1-ferrocenyl-ethylamine ((*R*)- and (*S*)-Ugi’s amine) obtained while using the (*S,S*)-SINpEt and (*R,R*)-SINpEt functionalized Au surfaces as electrodes, respectively. The ORR polarization curves of (c) (*S*,*S*)-SINpEt and (d) (*R*,*R*)-SINpEt functionalized Au surface at different thicknesses, controlled by the different deposition times. (e) The difference in the currents in the LSV diagram obtained from (*S*,*S*)-SINpEt and (*R*,*R*)-SINpEt functionalized Au surface with different thicknesses. (f) The difference in the onset potential in the LSV diagram obtained from (*S*,*S*)-SINpEt and (*R*,*R*)-SINpEt functionalized Au surface with different thicknesses. (g) Current/thickness plot of the (*S*,*S*)-SINpEt and (*R*,*R*)-SINpEt functionalized Au surfaces with different thicknesses tuned by different deposition time during the chemical vapor deposition.

### NHC SAM as Spin Filters in Oxygen Reduction Reaction (ORR)

It is well-known that spin-polarized electron transfer can facilitate the multielectron reduction of diatomic oxygen during the ORR. Since chiral biomolecules are known to generate spin-polarized electrons through the CISS effect, they may serve as effective mediators for enhancing ORR performance and efficiency.^30^ For this purpose, we fabricated (*S*,*S*)-SINpEt and (*R*,*R*)-SINpEt thin films on the Au surface and utilized them as electrodes in the oxygen reduction reaction (ORR). In the past, it was shown that the thickness of the chiral film plays an important role in determining the efficiency of the reaction. It was suggested that the thickness of the layer affects the degree of coherency of the pair of electrons transferred to the oxygen.^56^ By varying the chemical vapor deposition time (10 min, 20 min, 30 to 60 min), we could vary the thickness of the (*S*,*S*)-SINpEt thin films (∼1.01 nm, ∼1.1 nm, ∼1.29 nm to ∼1.43 nm) and (*R*,*R*)-SINpEt thin films (∼0.75 nm, ∼0.91 nm, ∼1.08 nm to ∼1.40 nm) on the Au surface. The AFM scratching method was employed on (*R*,*R*)-SINpEt@Au surfaces to determine the thickness of the NHC thin films (Figure 3c–j). Using the contact mode, forces were applied to remove the SINpEt molecules from the surface, while keeping the gold substrate unaffected. We correlated the molecular coverage (number of NHC molecules in a 1 nm^2^ area) on the Au surface using XPS (Figure 3l) with the deposition time. As deposition time was longer, the molecular coverage increased for both (*S*,*S*)-SINpEt and (*R*,*R*)-SINpEt. The electrodes with different film thicknesses were used in the ORR studies in a three-electrode cell setup. The reference electrode was a Hg/Hg_2_Cl_2_/saturated KCl (saturated calomel electrode), and the counter electrode was a platinum wire. A 0.1 M KOH solution served as the electrolyte at a pH of 13. Linear sweep voltammograms (LSV) were acquired for different thicknesses of NHCs. The efficiency of ORR can be evaluated from various factors, i.e., onset potential, onset current, peak potential, and peak currents. Before each ORR measurement, the electrolyte was purged with O_2_ for at least 30 min, and an O_2_ atmosphere was maintained during the measurement to ensure a stable and reproducible oxygen concentration at the electrode–electrolyte interface. To investigate the role of spin polarization in the ORR, we compared electrodes modified with chiral and achiral NHC thin films, as only the chiral coatings are capable of inducing spin-polarized electron transport through the CISS effect. Figure 5c,d revealed that as the thickness of chiral NHCs increased, both the reduction current and reduction potential changed correspondingly with thickness or deposition time. Hence, all those parameters were correlated. The figures clearly demonstrated that prolonged deposition times led to a positive shift in the ORR reduction potential, together with a reduction in the cathodic current. These observations indicated a significant influence of the deposited layer on the electrochemical reduction of oxygen. Given that chiral thin films can generate spin-polarized electron currents while achiral thin films cannot, a comparative investigation of the ORR on chiral and achiral molecular coatings was conducted to assess the role of spin polarization in the ORR process. As evident from Figure S39 (Supporting Information Section S-12), the ORR on achiral NHC films occurred at a higher onset potential of approximately 0.1 V compared to the corresponding chiral NHC films and was accompanied by significantly lower reduction currents (Supporting Information Section S-11). The onset potential is defined as the potential at which the current reaches a value of 0.025 mA. It is important to mention that a higher reduction potential corresponds to a higher barrier for the ORR. The observations provided further evidence that spin polarization induced by chiral NHC layers played a crucial role in enhancing ORR activity, leading to improved electron-transfer kinetics and catalytic performance. To further elucidate the role of spin coherence in chiral thin films of varying thicknesses, two key parameters, namely the onset current and onset potential, were plotted as a function of film thickness (Figure 5). Figure 5e,f showed the onset current and potential as a function of the film thickness, revealing a decrease in current with increasing thickness, accompanied by a weak oscillatory feature. In past studies, oscillations in current relative to molecular length have been seen in double-stranded DNA^41,55,57^ and peptide nucleic acids (PNA).^58^ The existence of oscillations indicated coherent conduction through the molecules.^53,56^ In the present study, the current and voltage abruptly decreased at a thickness of about 0.7 nm; for thicker films, the variation in those parameters as a function of thickness was very weak. We suggest that the abrupt change observed was related to the loss of coherence between the electrons in each pair transferred to the oxygen, similarly to what was observed before.

### Theoretical Calculations

To investigate the geometry of the chiral NHCs on the surface, density functional theory (DFT) calculations^59,60^ were performed on a variety of geometries and supercell sizes corresponding to different surface coverages (Figure 6, Supporting Information Section S-13).

**Figure 6:**
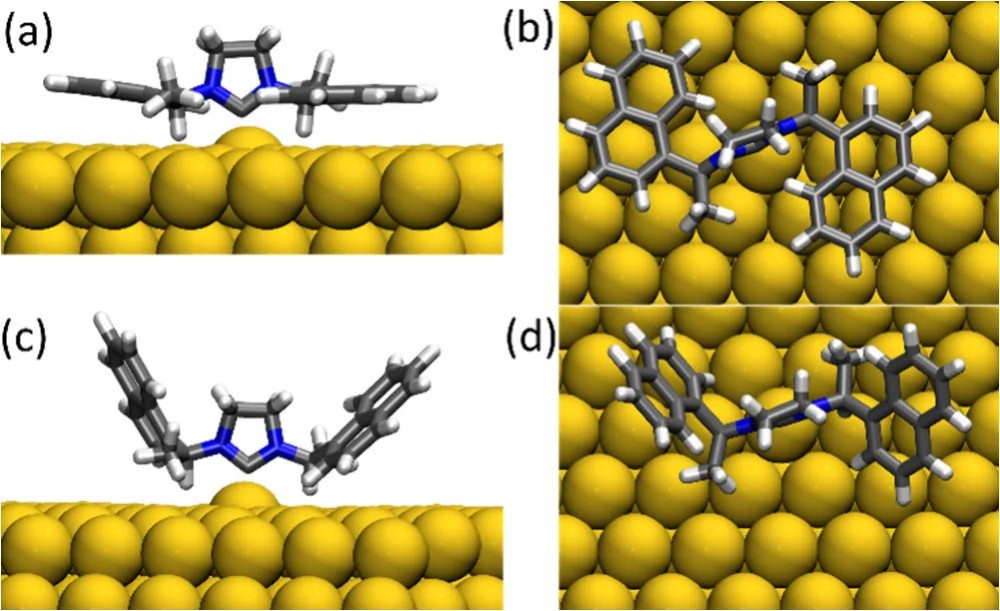
Optimized geometry of the molecule on a 11 × 9 surface. (a) Side view of the flat geometry. (b) Top view of the flat geometry. (c) Side view of the upright geometry. (d) Top view of the upright geometry.

To determine the molecular coverage, the surface area of the Au(111) metal slab was calculated for a given supercell (Supporting Information Section S-13). Comparing with experimental results from Figure 3l, a surface unit cell of size 6 × 4 or 7 × 3 (i.e., a coverage of 0.6–0.7 molecules/nm^2^) seems to fit with coverage measurements at early deposition times, while for later deposition times, a cell size of 4 × 3 or 5 × 3 (0.9–1.1 molecules/nm^2^ coverage) matches our XPS experiments. Initial optimizations were performed on a 11 × 9 × 3 Au(111) slab for both the flat and upright geometries to obtain relaxed structures of individual molecules with negligible neighbor–neighbor interaction. This yielded the structures shown in Figure 6a,b for the flat geometry and Figure 6c,d for the upright geometry at very low coverage. The upright geometry was preferred by 2.21 eV based on calculations of the adsorption energy, as seen in Table 1.

**Table 1:** Height of the Molecules Relative to the Top Gold Layer in nm, Adsorption Energy (*E*_ads_) in eV, and Charge Transferred to the Surface (*q*_CT_) in Units of Elementary Charge for Flat and Upright Geometry on a 11 × 9 Surface Cell

geometry	height [nm]	*E*_ads_ [eV]	*q*_CT_
flat	0.57	–3.21	0.56
upright	0.87	–5.33	0.43

Based on the optimized geometries in the low-coverage case, geometry optimizations were performed for the high-coverage case of a 5 × 3 surface unit cell, which yielded the geometries as shown in Figure 7a–d. Optimizations on a 4 × 3 surface have so far led to unphysical deformations of the surface. Therefore, the calculations suggested a coverage of 0.93 molecules/nm^2^ for the 5 × 3 surface cell (Figure 7), closely matching the experimental value of 1.06 molecules/nm^2^ for large deposition times shown in Figure 3l.

**Figure 7:**
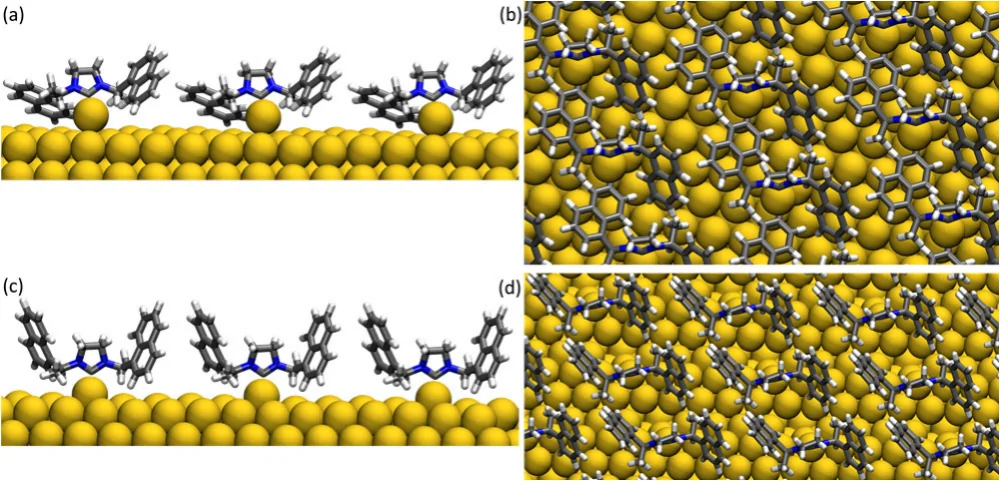
Optimized geometries of molecules on a 5 × 3 surface with a few periodic images. (a) Side view of the flat geometry. (b) Top view of the flat geometry. (c) Side view of the upright geometry. (d) Top view of the upright geometry.

For the flat configuration, a ballbot-type geometry is favored, while in the upright case, a gold atom is partially pulled out of the surface. Energetically, the flat geometry is preferred by 0.82 eV compared to the upright geometry (cf. Table 2). Figure 7a reveals that the wings of the molecules are tilted with respect to the surface instead of being parallel to the surface due to periodic interactions with neighboring molecules. Similarly, in the case of the upright molecule at increased coverage, the increasing interactions with neighbors lead to both wings becoming even more upright. In both cases, the molecular packing density (i.e., the coverage) increased as a result. From the optimized geometries, the height of the molecules relative to the surface could be measured (Tables 1 and 2).

**Table 2:** Height of the Molecules Relative to the Top Gold Layer in nm, Adsorption Energy (*E*_ads_) in eV, and Charge Transferred to the Surface (*q*_CT_) in Units of Elementary Charge for Flat and Upright Geometry on a 5 × 3 Surface Cell

geometry	height [nm]	*E*_ads_ [eV]	*q*_CT_
flat	0.75	–3.19	0.37
upright	0.93	–2.37	0.32

The height of the energetically favored flat geometry was predicted to be 0.75 nm, which is significantly below the experimental value for a complete monolayer of 1.40 nm (Figure 3k). This discrepancy is likely due to experimental uncertainties related to the AFM measurements (e.g., tip scratching the gold surface). As a consequence, AFM measurements are unable to resolve the small height difference of ∼0.2 nm between the flat and upright geometries.

In addition, the charge density redistribution upon adsorption has been analyzed for the different geometries and coverages (Tables 1 and 2, Figures S40–S43). By integrating the charge density difference over the Au slab, the amount of charge transferred from the NHC molecule to the surface was calculated. It is evident that the flat geometries lead to a more pronounced charge transfer due to the larger contact region with the surface. Furthermore, charge transfer is less pronounced at high coverage, as intermolecular interactions prevent the molecules from maximizing their contact area with the surface.

## Conclusions

In summary, for the very first time, we fabricated chiral NHC (SINpEt) thin films on an Au (8 nm) protected Ni (120 nm) surface for efficient electronic spin filtration. Surprisingly, despite such a small molecular structure with light elements, the NHC thin films showcased excellent electronic spin polarization of up to 60% with (*S*,*S*)-SINpEt and (*R*,*R*)-SINpEt thin films, which was unprecedented before. The chiral NHC-functionalized Au surface was further successfully utilized as a working electrode for ORR in 0.1 M KOH aqueous solution at pH = 13. (*S*,*S*)-SINpEt and (*R*,*R*)-SINpEt thin films with different thicknesses on the Au surface were fabricated with a range from ∼0.75 nm to ∼1.43 nm. The thickness-dependent ORR was also investigated using (*S*,*S*)-SINpEt and (*R*,*R*)-SINpEt thin films of different thickness coated on Au surfaces. The structural modularity of the NHCs (position of chiral substituents, number of chiral centers, electronic and steric tunability) could help to exhibit a huge library of chiral molecules. Consequently, the screening of the CD spectra and their spin polarization ability of the diverse and wide library of NHCs could help to establish a structure–property relationship and to understand the precise electronic spin filtration mechanism. Thus, the class of NHCs as organic small molecules could engineer the CISS effect and manipulate well-established and conventional electrochemical processes for developing robust and durable next-generation energy devices

## Supplementary Material



## Data Availability

All data are available in the main text or Supporting Information.

## Notes

Details of the experimental section, supporting experiments, details of the synthetic procedures, and experimental results (PDF)
