# Supplementary material for: Chiral N‑Heterocyclic Carbene On-Surface Induced Electronic Spin Filtration
Source: J Am Chem Soc. 2026 Aug 4;148(32):34173–83. doi: 10.1021/jacs.6c04854 (PMC13495767; doi:10.1021/jacs.6c04854)
Supplement: Supplementary file 1 [file ja6c04854_si_001.pdf]

(Electronic Supporting Information)

Chiral N-heterocyclic carbene on-surface induced electronic spin filtration

Himadri Sekhar Sasmal,[†,1] Anu Gupta,[†,2] Arne Nalop,[1] Deb Kumar Bhowmick,[2] Dominik Schwab,[3] Bonnie J. Tyler,[4] Nikos Doltsinis,[*,3] Ron Naaman,[*,2] and Frank Glorius[*,1]

[1]Organisch-Chemisches Institut, Universität Münster, Corrensstraße 36, 48149 Münster, Germany

[2]Department of Chemical and Biological Physics, Weizmann Institute of Science, Rehovot 76100, Israel

[3]Institut für Festkörpertheorie and Center for Multiscale Theory and Computation, Universität Münster, Wilhelm-Klemm-Str. 10, 48149 Münster, Germany

[4]Center for Soft Nanoscience (SoN), University of Münster, Busso-Peus-Strasse 10, 48149, Münster, Germany

*Email: nikos.doltsinis@uni-muenster.de; Tel: +49 251 83-33582.

*Email: ron.naaman@weizmann.ac.il; Tel: +972 8 934-2367.

*Email: glorius@uni-muenster.de; Tel: +49 251 83-35345.

Table of contents

Section		Page No
S-1	General information	S3-S6
S-2	Experimental section	S6-S14
S-2a	Synthetic procedures	S6-S10
S-2b	NHC thin film fabrication on the Au surface	S10-S14
S-3	UV-Vis and CD spectra	S15-S16
S-4	XPS analysis	S17-S24
S-5	Molecular coverage calculations	S25-S28
S-6	ToF-SIMS analysis	S28-S31
S-7	Contact angle measurement	S32
S-8	Electrochemical stability	S33-S34
S-9	Spin-dependent cyclic voltammetry	S34-S35
S-10	mcAFM measurements	S36
S-11	ORR with chiral Au surface	S36-S37
S-12	ORR control experiment with achiral Au surface	S38-S40
S-13	Theoretical calculations	S42-S45
S-14	References	S45-S48

Section S-1: General information

^{1}H and ^{13}C nuclear magnetic resonance (NMR) spectra were recorded on a Bruker AV 400 at room temperature. Chemical shifts (δ) were presented in ppm units. The residual solvent signals were used as references, and the chemical shifts were converted to the TMS scale (MeOD: δ_H = 4.87 ppm, δ_C = 49.00 ppm; CD_2Cl_2: δ_H = 5.32 ppm, δ_C = 54.00 ppm; D_2O: δ_H = 4.79 ppm). All the NMRs were processed using Mestrenova 14, applying standard phase and baseline corrections. Coupling constants (J) were quoted in Hz.

Mass spectra were recorded in electrospray ionization (ESI) mode using a high-resolution mass spectrometer (HRMS) from Thermo Fisher Scientific (Orbitrap Exploris 120 Electrospray). ESI spectra show the relative abundance, after normalization to the maximum signal intensity (NL), as a function of the mass-to-charge ratio (m/z).

X-ray photoelectron spectra were measured using a K-Alpha X-ray photoelectron spectroscopy **(XPS)** instrument made by Thermo Fisher Scientific. For XPS measurements of the C 1s, O 1s, N 1s, and Au 4f core levels, a separate UHV system (base pressure 3×10^{-8} Pa) was used hosting a hemispherical energy analyzer (SPECS Phoibos 100) using monochromatized Al Kα X-rays with hν = 1486.7 eV (SPECS FOCUS 500 Ellipsoidal Crystal Monochromator and X-Ray source SPECS XR50 M) yielding an instrumental resolution of ~0.5 eV. The measured XPS spectra were deconvoluted using Avantage software. The binding energy scale was calibrated to the Au 4f line at 84.0 eV using an Au foil in direct contact with the sample holder.

Time-of-Flight Secondary Ion Mass Spectrometry (ToF-SIMS) measurements were executed using a custom ION-TOF™ (Münster, Germany) instrument, which is largely equivalent to the ION-TOF M6 instrument. For the images, a 30 kV Bi_3^+ primary ion source with a spot size of approximately 100 nm, a pulse length of 100 ns, and a pulsed current of 0.03 pA was used. The images were collected using the delayed extraction mode of the M6 analyzer, which provided better than 6000 m/Δm mass resolution without degradation of the lateral image resolution. For all images, the primary ion dose density was kept within the static ion limit, i.e., $\sim 10^{12}$ ions cm^{-2}. The SIMS spectra and images were collected and analyzed using the Surface Lab 7.1 (IONTOF) software package. The data was exported from the Surface Lab 7.1 (ION-TOF) software package and processed in MatLab™.

Circular Dichroism (CD) and Ultraviolet-Visible (UV-Vis) spectroscopy measurements were performed using a Chirascan spectrometer (Applied Photophysics, England) with a thermoelectrically controlled single-cell holder. The measurements were performed at a 2-second time per point, with a 1 nm step size and a bandwidth of 1 nm. The optical path of the quartz cuvette used was 0.5 cm.

Magnetic-conductive atomic force microscopy (mc-AFM)

The mc-AFM measurements were performed at room temperature. The AFM system is equipped with a Besocke-Beetle inertial Ambient AFM, which is controlled by an R9 electronics controller (RHK Technology). The entire AFM system is housed in an HV-7V electromagnet, which was purchased from Laboratoria Elettrofisico Walker LDJ Scientific, allowing a continuously adjustable magnetic field (from 0 to 1T). The current signal was taken by applying voltage ramps between a platinum-coated silicon tip and the surface. Cantilever B (nominal force constant 2.7 N/m) of a platinum-coated silicon tip from Micromasch (HQ:DPE-XSC11) was used in the measurements. The force exerted on the sample by the tip was set to be 8-10 nN. At least 70 consecutive individual *I-V* curves under an external magnetic field of 0.5 T for both magnetic field orientations of north pole up and down were collected at different positions. The representative results of mc-AFM experiments are shown in Section S-10. The results shown in the manuscript are the average value of all the individual recorded curves. AFM topographies were obtained by Bruker-AFM MultiMode 8 with a silicon tip (Model AC240TS-R3 from Oxford Instruments). For measuring the thickness of films, two back-to-back AFM imaging techniques have been employed. First, AFM tapping mode imaging was performed; after obtaining an aggregate-free uniform layer using tapping mode topography, we switched to contact mode for AFM nanoshaving. For nanoshaving, we used the same tip without changing the sample position. The spring constant of the AFM cantilever was calibrated by deflection sensitivity (α) calculation and thermal-K tuning operation. The precise contact force delivered by the AFM cantilever was determined using the deflection sensitivity (the reciprocal of the slope from the linear region of the force-distance curve in the repulsive regime) and the spring constant (k) acquired from thermal-K measurements of the AFM cantilever.

$$\text{Contact force [nN]} = (\text{Setpoint} - \text{Free deflection [volt]}) \times \alpha \text{ [nm/volt]} \times k\text{[nN/nm]}$$

For all samples, nano-shaving was performed over ~500×500 nm^2 scan area with 130-150 nN applied contact force, which was effective for the removal of NHCs from the Au substrate. To obtain a clear topographic image of the nano-shaved region, we moved to tapping mode following the nano-shaving of the film. The thickness of the film was obtained from the subtraction between the RMS roughness of the non-scratched area and the RMS roughness of the scratched area. *Gwyddion-2.66* software was used for processing the AFM images.

Contact Angle (CA) measurements executed with a DSA 100 from Krüss. Deionized water was used for the droplets, and their volume was set to 1 μL at a rate of 2 μL/s. The data were analyzed with Krüss Advance Drop Shape (OpenCV v.3.1, Moq v.4.7.99, NLog, v.4.412, Swashbuckle.Net v.5.2.1).

Cyclic Voltametry (CV) and Electrochemical data were recorded at room temperature on a potentiostat (PalmSens4) electrochemical workstation, by employing a typical three-electrode electrochemical cell arrangement. For the chiral ferrocene experiment, a chiral redox probe of 0.8 mM (*R* or *S*) chiral *N,N* -dimethyl-1-ferrocenyl-ethylamine in a 10 mM potassium phosphate buffer solution at pH 7 was used. A Pt wire and a saturated calomel electrode (SCE) were used as the counter and reference electrodes, respectively. The working electrode area of 0.25 cm^2 was fixed in all the measurements. The electrolyte for ORR was 0.1 M aqueous KOH (pH 12.6). Before each measurement, the electrolyte was purged with O_2 for 30 mins. During the measurement, the needle for O_2 purging was removed from the solution to maintain a stable current. However, it was kept on top of the solution to avoid a change in O_2 concentration. The oxygen reduction data were taken at room temperature using linear sweep voltammograms (LSV) at a scan rate of 50 mV/s.

Section S-2: Experimental section

All reactions were carried out in oven-dried glassware with oven-dried Teflon-coated magnetic stir bars. Dry solvents were either taken from a solvent purification system (HPLC grade, dried over activated alumina columns) or purchased from Acros Organics, Sigma-Aldrich, or Carl Roth (stored over activated molecular sieves). All reagents were obtained from Acros Organics, Fisher Scientific, Merck, Sigma-Aldrich, and were utilized as received.

Materials

Starting materials (*S*)-1-(1-naphthyl)ethylamine and (*R*)-1-(1-naphthyl)ethylamine were purchased from Sigma-Aldrich (produced by BASF). 1,2-dibromoethane was bought from Merck. Triethyl orthoformate and Ammonium tetrafluoroborate were purchased from Acros Organics and Fisher Scientific, respectively. Amberlyst^{TM} A-26 (OH) ion exchange resin was bought from Acros Organics. All the chemicals were used as received without any further purification.

Section S-2a: Synthetic procedures

Synthesis of (*S*,*S*) and (*R*,*R*)–SINpEt(H)·[BF_4]:

1,3-Bis((*S*)-1-(naphthalen-1-yl)ethyl)-4,5-dihydro-1H-imidazol-3-ium tetrafluoroborate, descripted as (*S*,*S*)–SINpEt(H)·[BF_4] and 1,3-Bis((*R*)-1-(naphthalen-1-yl)ethyl)-4,5-dihydro-1H-imidazol-3-ium tetrafluoroborate, descripted as (*R*,*R*)–SINpEt(H)·[BF_4] were synthesized as following the previous synthesis procedure.[1]

In the first step, (*S*)-1-(1-naphthyl)ethylamine (**I**) (642 mL, 4.0 mmol, ee>99%) and 1,2-dibromoethane (164 mL, 2.0 mmol) were mixed in a dry Schlenk tube. The Schlenk was then flushed with argon, sealed, and heated to 100 °C overnight. The resulting solid was taken up in CH_2Cl_2 (50 mL) and aqueous NaOH (1 M). The pH of the basified solution was maintained at pH~13 using aqueous NaOH (2 M), and extracted with CH_2Cl_2 (3 x 20 mL). The combined organic layers were dried (Na_2SO_4) and concentrated in vacuo to furnish the respective bis-amino compound (**II**), which was used directly for the next step without further purification. To compound **II** (800 mg, 2.17 mmol), NH_4BF_4 (227.8 mg, 2.17 mmol), and $CH(OEt)_3$ (361 mL, 2.17 mmol) was added in a dry Schlenk tube as before, and the resulting mixture was heated to 120 °C overnight. After cooling to room temperature, EtOH was removed in vacuo.

The crude containing the product (**III**) was washed with hexane (10 mL) and Et_2O (10 mL). The product (*S*,*S*)–SINpEt(H)·$[BF_4]$ was purified by re-crystallization in EtOH/Et_2O. The (*S*,*S*)–SINpEt(H)·$[BF_4]$ (**III**) was isolated as a white crystalline solid (~420 mg, 0.9 mmol, 45% yield).

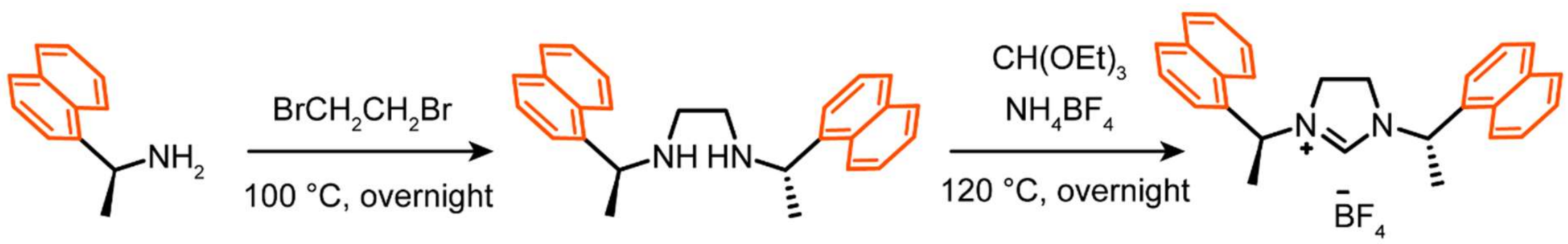

I II III

Figure S1: Scheme for the synthesis procedure of (*S*,*S*)–SINpEt(H)·$[BF_4]$.

^{1}H NMR (400 MHz, $CDCl_3$): 8.64 (s, 1H), 8.04 (d, *J* =8.9 Hz, 2H), 7.89(dd, *J* =8.1 Hz, 1.5 Hz, 2H), 7.86-7.81 (m, 2H), 7.61-7.51 (m, 4H), 7.47-7.43 (m, 4H), 5.83 (q, *J* =6.8 Hz, 2H), 3.77-3.66 (m, 2H), 3.61-3.49 (m, 2H), 1.94 (d, *J* =6.8 Hz, 6H).

^{13}C NMR (75 MHz, $CDCl_3$): 155.8, 134.2, 132.8, 130.6, 129.9, 129.4, 127.7, 126.5, 125.5, 124.3, 122.1, 54.5, 46.7, 19.2.

^{19}F NMR (375 MHz, $CDCl_3$): −151.78 (bs), −151.83 - −151.84 (q).

ESI-MS: calculated for $[C_{27}H_{27}N_2]^+$: 379.2169, found: 379.21652.

Following an exact similar procedure, we also synthesized (*R*,*R*)–SINpEt(H)·$[HBF_4]$. (*R*)-1-(1-naphthyl)ethylamine (**IV**) (642 mL, 4.0 mmol, ee>99%) and 1,2-dibromoethane (164 mL, 2.0 mmol) were taken as the starting materials. The similar synthesis strategy resulted in (*R*,*R*)–SINpEt(H)·$[HBF_4]$ (**VI**), which was recrystallized in EtOH/Et_2O to obtain a white crystalline solid (~382 mg, 0.82 mmol, 41% yield).

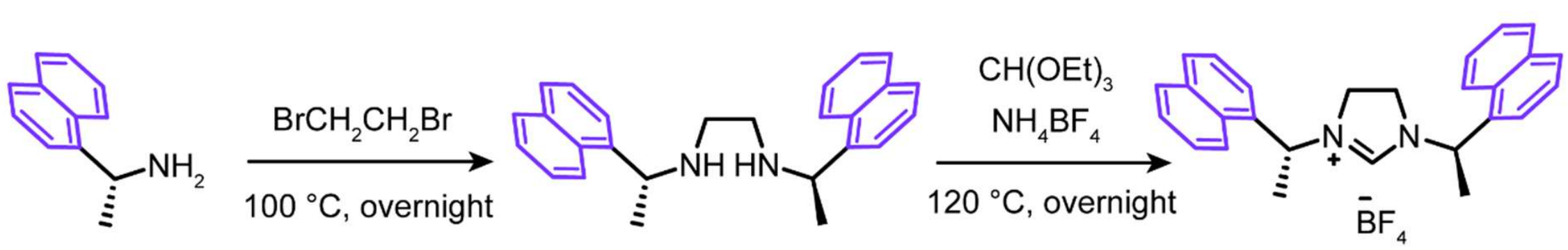

IV V VI

Figure S2: Scheme for the synthesis procedure of (*R*,*R*)–SINpEt(H)·$[BF_4]$.

^{1}H NMR (400 MHz, CDCl$_3$): 8.66 (s, 1H), 8.04 (d, J =8.5 Hz, 2H), 7.89 (dd, J =8.1 Hz, 1.5 Hz, 2H), 7.86-7.81 (m, 2H), 7.61-7.51 (m, 4H), 7.48-7.42 (m, 4H), 5.83 (q, J =6.8 Hz, 2H), 3.77-3.65 (m, 2H), 3.61-3.50 (m, 2H), 1.94 (d, J =6.8 Hz, 6H);

^{13}C NMR (75 MHz, CDCl$_3$): 155.5, 133.9, 132.5, 130.3, 129.6, 129.1, 127.4, 126.2, 125.2, 124.0, 121.8, 54.2, 46.3, 18.9

^{19}F NMR (375 MHz, CDCl$_3$): −151.77 (bs), −151.81 - −151.83 (q).

ESI-MS: calculated for $[C_{27}H_{27}N_2]^+$: 379.2169, found: 379.21692.

Synthesis of (*S*,*S*) and (*R*,*R*)–SINpEt(H)·[HCO$_3$]:

In the adoption of a literature procedure[2], (*S*,*S*) and (*R*,*R*)–SINpEt(H)·[HCO$_3$] were prepared from the (*S*,*S*) and (*R*,*R*)–SINpEt(H)·[BF$_4$], respectively. At first, a hydrogen carbonate exchange resin was prepared from Amberlyst^{TM} A-26 (OH) ion exchange resin. To accomplish the conversion, 25 g of the resin was suspended in 50 mL of deionized water (pH ~ 8), and carbon dioxide was bubbled through the solution for 30 minutes (pH ~ 6).
After this, Resin-HCO$_3$. suspended in water, was taken in a typical fritted column used for silica chromatography. The column was flushed with water and then with MeOH (100 mL, twice). Following the first cycle, the column was gently rotated to remove air bubbles from the resin material and ensure proper packing. After the settlement of all resin material, the MeOH was eluted to the level of resin in the column.

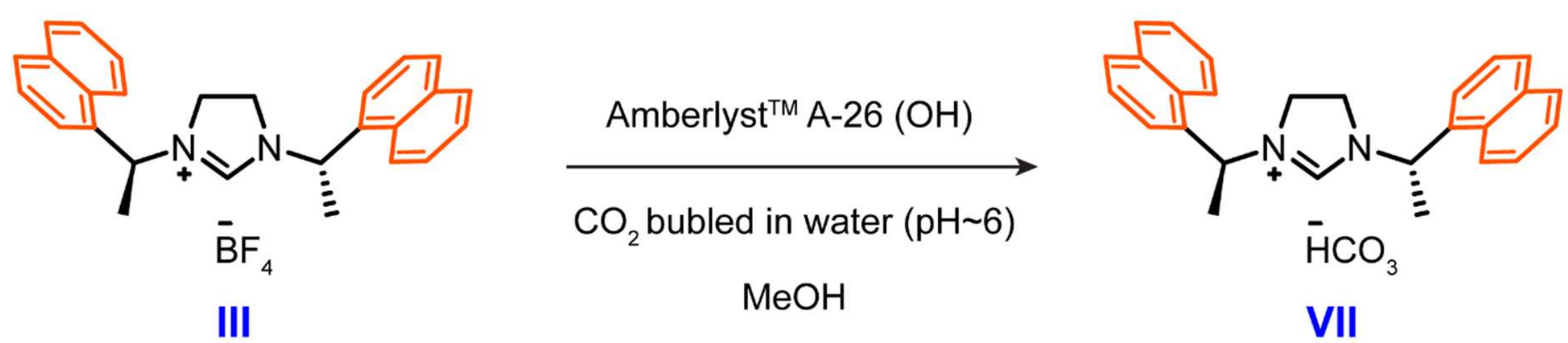

Figure S3: Scheme for the synthesis procedure of (*S*,*S*)–SINpEt(H)·[HCO$_3$].

(*S*,*S*)–SINpEt(H)·[BF$_4$] (100 mg, 0.214 mmol) was dissolved in MeOH (10 mL) and added onto the resin. The solvent was eluted to allow the molecules to penetrate the resin; then, the column was refilled with MeOH (100 mL). The solvent was collected in reaction tube fractions and checked for the elution of the corresponding bicarbonate salt through TLC plate staining under UV light (254 nm) exposure. The positive fractions were combined, and the solvent was removed by rotary evaporation (temperature should not exceed 25 °C). Drying of the remaining

residue in an oil pump vacuum (<1 mbar) resulted in a white solid, which was further recrystallized in EtOH/Et_2O at low temperatures (-5 °C to 0 °C) to obtain a white crystalline solid (*S*,*S*)–SINpEt(H)·[HCO_3] (56.1 mg, 0.127 mmol) with 59% yield. The crystalline white solid was characterized by NMR, HRMS (ESI), and Direct-Inlet EI-MS measurements.

^{1}H NMR (400 MHz, CD_3OD): 7.97-7.91 (m, 6H), 7.59-7.49 (m, 8H), 5.75 (q, *J* =6.9 Hz, 2H), 3.82-3.70 (m, 2H), 3.62-3.53 (m, 2H), 1.88 (d, *J* =6.9 Hz, 6H). The C2 and HCO_3 protons could not be observed due to their rapid exchange with the deuterated solvent on the NMR time scale.

^{13}C NMR (75 MHz, CD_3OD): 165.3, 135.6, 134.0, 131.9, 130.9, 130.5, 128.4, 127.4, 126.5, 125.3, 122.9, 55.1, 47.7, 19.3. The carbene C2 is expected to be broadened by solvent exchange on the time scale of the NMR measurement.

ESI-MS: calculated for $[C_{27}H_{27}N_2]^+$: 379.2169, found: 379.21656.

Direct inlet EIMS: m/z (% rel. abundance) calculated for $[M]^{\cdot+}$ = $[C_{27}H_{26}N_2]^{\cdot+}$ 378.2, at 150 (±20) °C, found $[M+H_2O]^{\cdot+}$ 396.2.

(*R*,*R*)–SINpEt(H)·[BF_4] (100 mg, 0.214 mmol) was taken to synthesize the corresponding bicarbonate salts in a similar procedure. The ion exchange led to the formation of a white solid, which was further recrystallized in EtOH/Et_2O at low temperatures (-5 °C to 0 °C) to obtain a white crystalline solid (*R*,*R*)–SINpEt(H)·[HCO_3] (62.2 mg, 0.141 mmol) with 66% yield. The crystalline solid was characterized by NMR, HRMS (ESI), and Direct-Inlet EI-MS measurements.

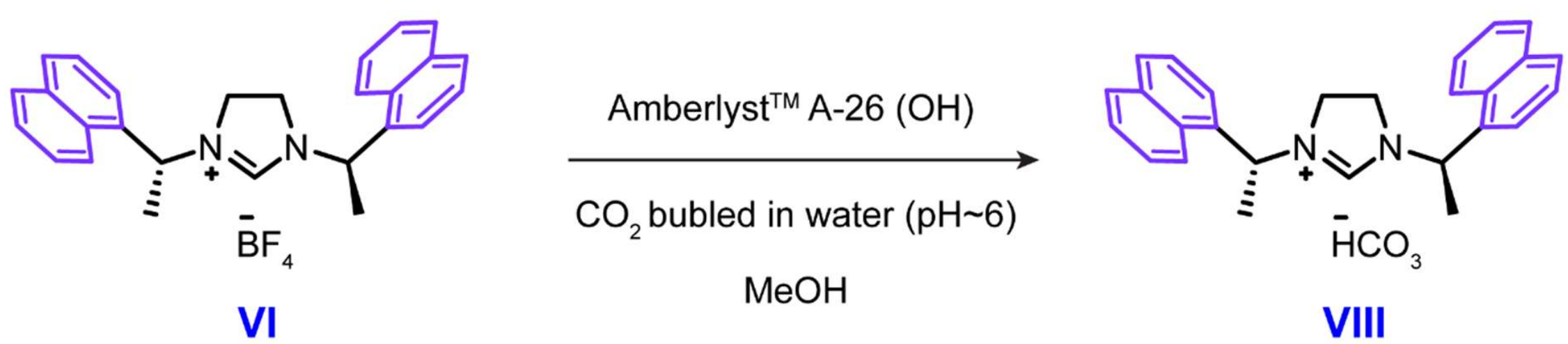

Figure S4: Scheme for the synthesis procedure of (*R*,*R*)–SINpEt(H)·[HCO_3].

^{1}H NMR (400 MHz, CD_3OD): 7.97-7.91 (m, 6H), 7.58-7.49 (m, 8H), 5.75 (q, *J* =6.9 Hz, 2H), 3.80-3.70 (m, 2H), 3.62-3.53 (m, 2H), 1.88 (d, *J* =6.9 Hz, 6H).The C2 and HCO_3 protons could not be observed due to their rapid exchange with the deuterated solvent on the NMR time scale.

^{13}C NMR (75 MHz, CD_3OD): 165.3, 135.6, 134.0, 131.9, 130.9, 130.5, 128.4, 127.4, 126.5, 125.3, 122.9, 55.1, 47.7, 19.3. The carbene C2 is expected to be broadened by solvent exchange on the time scale of the NMR measurement.

ESI-MS: calculated for $[C_{27}H_{27}N_2]^+$: 379.2169, found: 379.2168.

Direct inlet EIMS: m/z (% rel. abundance) calculated for $[M]^{\cdot+} = [C_{27}H_{26}N_2]^{\cdot+}$ 378.2, at 150 (±20) °C, found $[M+H_2O]^{\cdot+}$ 396.2.

Fabrication of Au-Ni surfaces:

The substrates are composed of a silicon wafer (University Wafer), on top of which several metal layers were deposited via e-beam evaporation: an 8 nm Ti layer, which serves as an adhesion layer, a 120 nm Ni layer, and an 8 nm Au layer. Here, the 8 nm Au acts as a protective layer preventing Ni oxidation. Thicker Au layers act as strong spin barriers, impeding spin polarization from reaching the Ni, while thinner Au layers allow for better transmission of spin polarization.

Fabrication of Au surfaces:

Au surfaces were fabricated on silicon wafers (University Wafer). An 8 nm Ti adhesion layer was first deposited, followed by the deposition of a 120 nm Au layer.

Section S-2b: NHC thin film fabrication on the Au surface

NHC thin films were fabricated on two different Au surfaces. In the first case (surface A), NHC thin films (with chiral NHC precursors) were fabricated on an Au-Ni (where an 8 nm thick Au layer was deposited on top of a 120 nm thick Ni layer) surface. The NHC molecules bound with the Au atoms present at the top part of the Au-Ni surfaces. Further, these NHC-functionalized Au-Ni surfaces were investigated for the spin-selective transport measurements using magnetic conductive atomic force microscopy (mcAFM).

In the second case (surface B), NHC thin films were fabricated on Au surfaces (120 nm thick) and were investigated as the spin filters in the oxygen reduction reaction (ORR). All surfaces (both surface A and B) were washed with hot Ethanol, followed by dry acetone. After washing, the surfaces were dried with argon flow before modification with NHC using different functionalization methods.

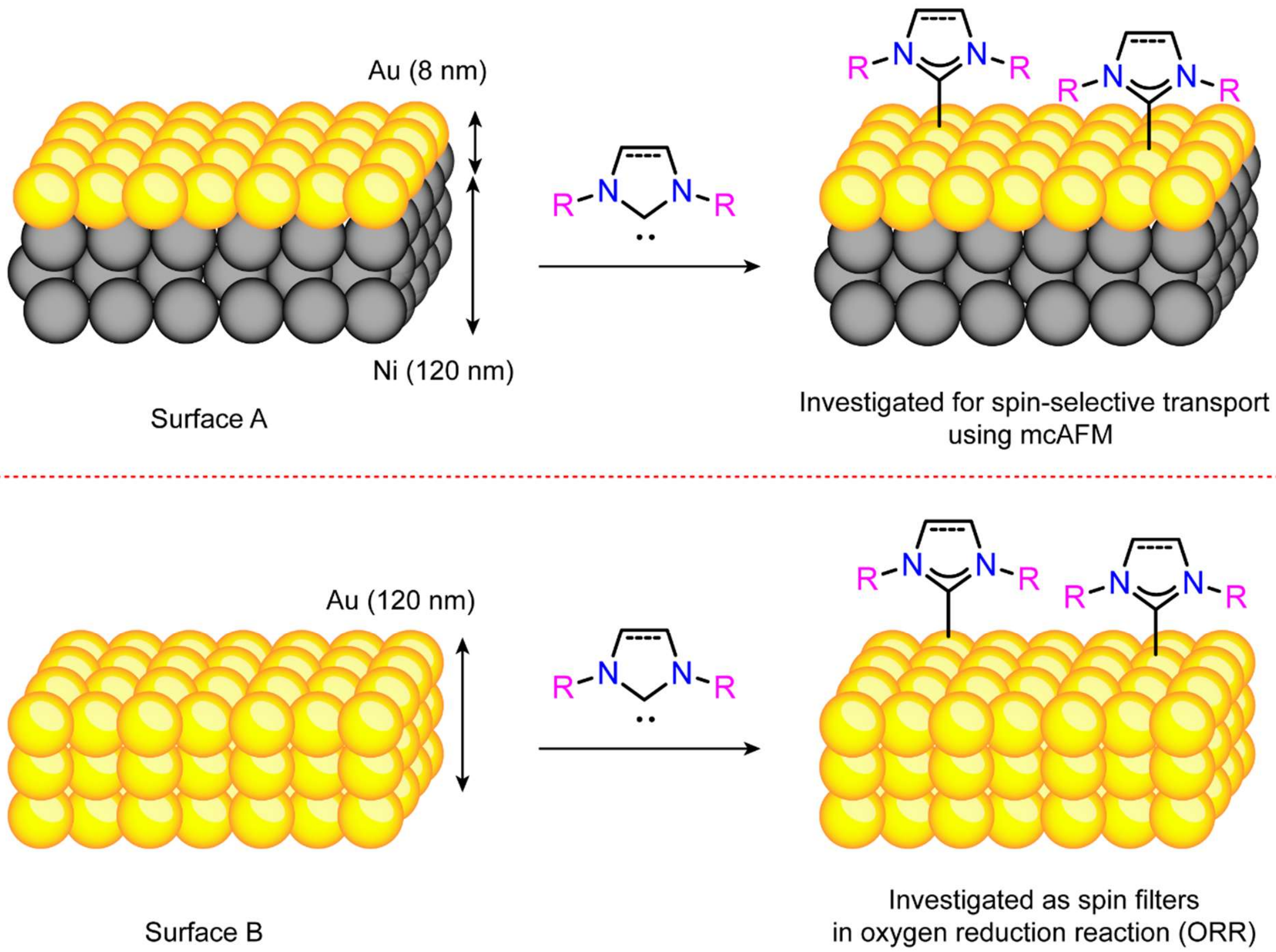

Figure S5: Types of surfaces on which the NHC thin films were fabricated using chiral NHC precursors.

The fabrication of NHC thin film on the Au surface (surface A) was attempted in three different procedures: i) in solution functionalization from NHC(H)·[HCO_3] salt, ii) free carbene functionalization from NHC(H)·[BF_4] salt, and iii) chemical vapour deposition from NHC(H)·[HCO_3] salt.

The first method of fabricating NHC thin film on the Au surface (surface A: Au-Ni surface), the Au surface was treated with a methanolic solution of (*S*,*S*)– and (*R*,*R*)–SINpEt·[HCO_3] separately for functionalization.[3] Both (*S*,*S*)–SINpEt(H)·[HCO_3] and (*R*,*R*)–SINpEt(H)·[HCO_3] (4.4 mg, 0.01 mmol) were dissolved in 5 mL of MeOH. Then, the washed Au surfaces were exposed to a methanolic solution (2 mM) of (*S*,*S*)–SINpEt(H)·[HCO_3] and (*R*,*R*)–SINpEt(H)·[HCO_3] separately for 2 hours at 40 °C in a screw cap vial. The bicarbonate salts released CO_2 and H_2O to generate the free carbene *in situ* in the methanolic solution and consequently functionalized the Au surfaces. Both the surfaces were further washed with HPLC-grade methanol, followed by HPLC-grade 2-propanol and acetone, dried, and preserved under Argon for characterization.

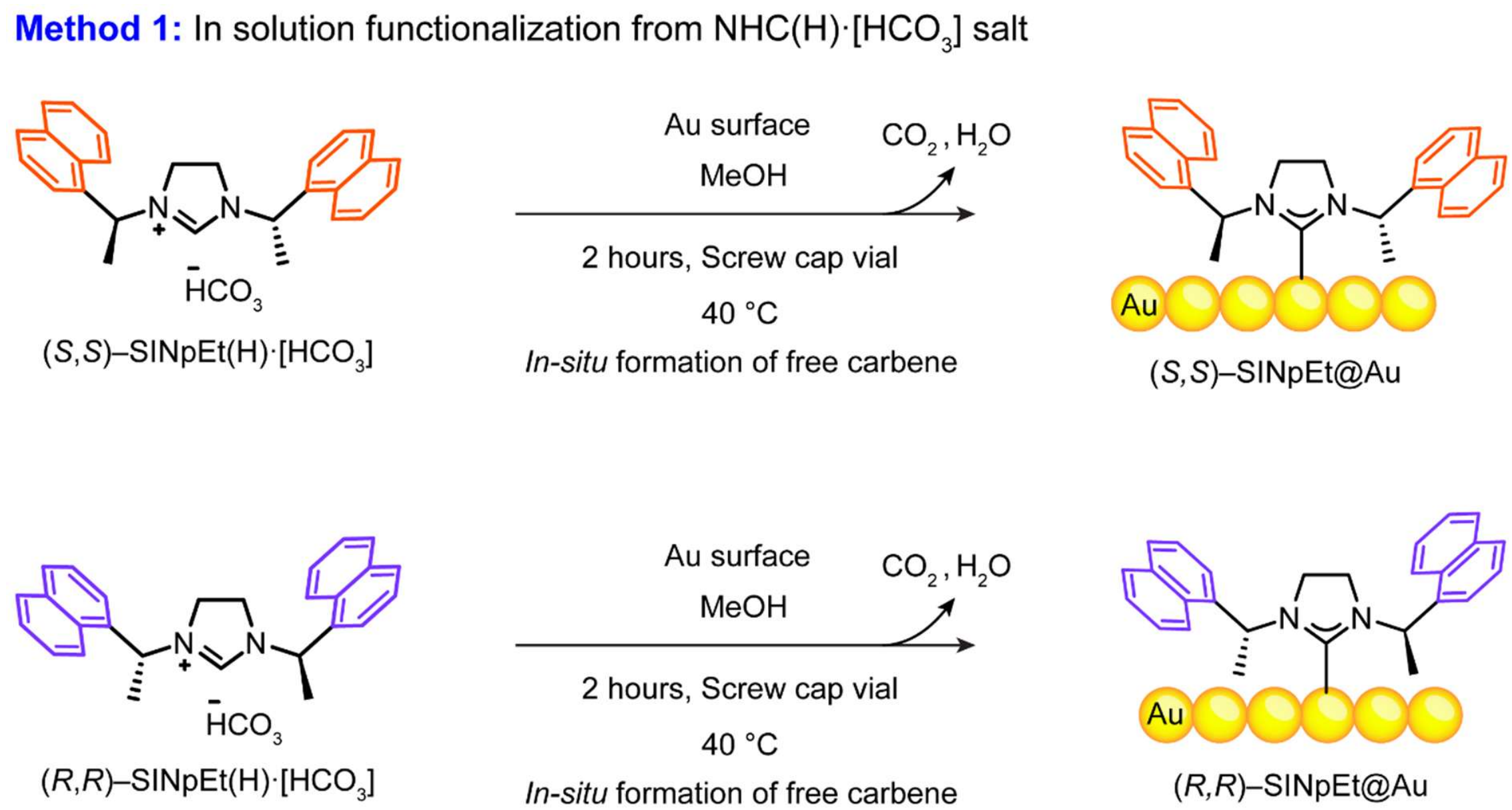

Figure S6: Schematic for the first method used for the fabrication of the (*S*,*S*)–, and (*R*,*R*)–SINpEt@Au thin film: free carbene functionalization from (*S*,*S*)–, and (*R*,*R*)–SINpEt·[BF_4] salt.

The second method of fabricating NHC thin film on the Au surface (surface A) involved non-nucleophilic strong potassium *tert*-butoxide (KO*t*Bu) to generate the free carbene *via* deprotonation of the (*S*,*S*)–SINpEt(H)·[BF_4] salt.[3] Because of its high reactivity and instability in ambient conditions, the reaction and the subsequent functionalization of the Au surface were performed under an argon atmosphere. At first, (*S*,*S*)–SINpEt(H)·[BF_4] (4.7 mg, 0.01 mmol) and KO*t*Bu (1.3 mg, 0.012 mmol) were mixed and stirred in 5ml dry toluene under an Argon atmosphere at room temperature (25 °C) for 4 hours. After that, the reaction mixture was filtered with Celite under an Argon atmosphere (filter frit). The filtrate is a free carbene [(*S*,*S*)–SINpEt] solution (concentration of ~2 mM), which was directly added to the cleaned and dried Au surface under an Argon atmosphere. Thus, the Au surface was exposed to the free carbene solution for 2 hours at 25 °C before it was washed (with HPLC-grade toluene, 2-propanol, and acetone), dried, and preserved under Argon for characterization.

Similarly, a thin film of (*R*,*R*)–SINpEt was also fabricated on the Au surface using the (*R*,*R*)–SINpEt(H)·[BF_4] salt.

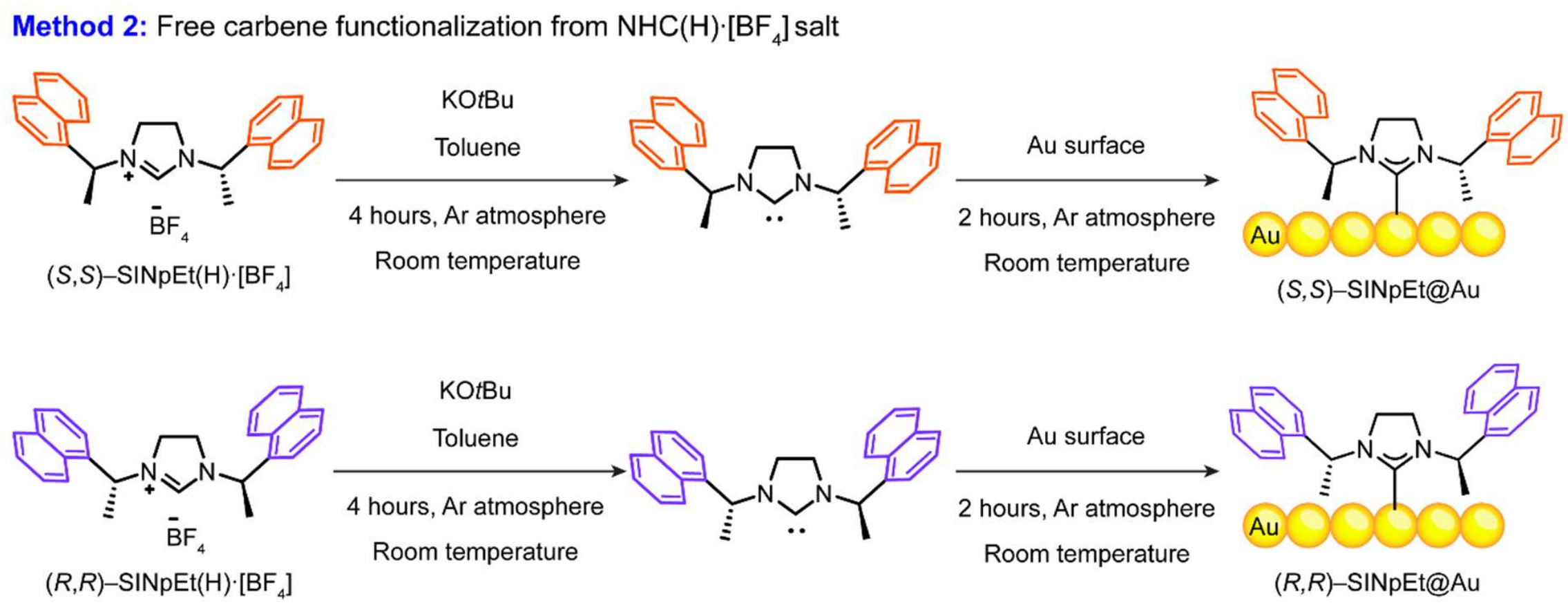

Figure S7: Schematic for the second method used for the fabrication of the (*S*,*S*)–, and (*R*,*R*)–SINpEt@Au thin film: in solution functionalization from (*S*,*S*)–, and (*R*,*R*)–SINpEt·[HCO_3] salt.

Inspired by the surface functionalization technique within the Scanning Tunneling Microscopy (STM), a vapor phase approach was subsequently developed as a third method.[4] A chemical vapour deposition (CVD) chamber was designed and developed in the lab with the help of the precision mechanics workshop of the Institute of Organic Chemistry at the University of Münster. Herein, within the CVD chamber, the (*S*,*S*)–SINpEt(H)·[HCO_3] salt (2 mg, ~0.0045 mmol) was heated under vacuum at 150-170 °C. During the heating under vacuum, bicarbonate salt liberated H_2O and CO_2 to generate the free carbene (*S*,*S*)–SINpEt. The free carbene forms within the CVD chamber reacts with the Au surface (both surface A and B individually) already placed within it using a surface holder (just on top of the bicarbonate salt). Because the primary side products of this procedure are H_2O and CO_2, this process is known to produce exceptionally clean NHC thin films. The time of vapour deposition was varied from 10 minutes to 60 minutes, depending on the desired thickness of the (*S*,*S*)–SINpEt@Au thin film. After cooling down the CVD chamber to room temperature, the surfaces were washed with HPLC-grade 2-propanol and acetone, dried, and preserved under Argon for characterization. A similar pathway was followed for the fabrication of (*R*,*R*)–SINpEt@Au thin film from the corresponding (*R*,*R*)–SINpEt·[HCO_3] salt (2 mg, ~0.0045 mmol) through chemical vapour deposition.

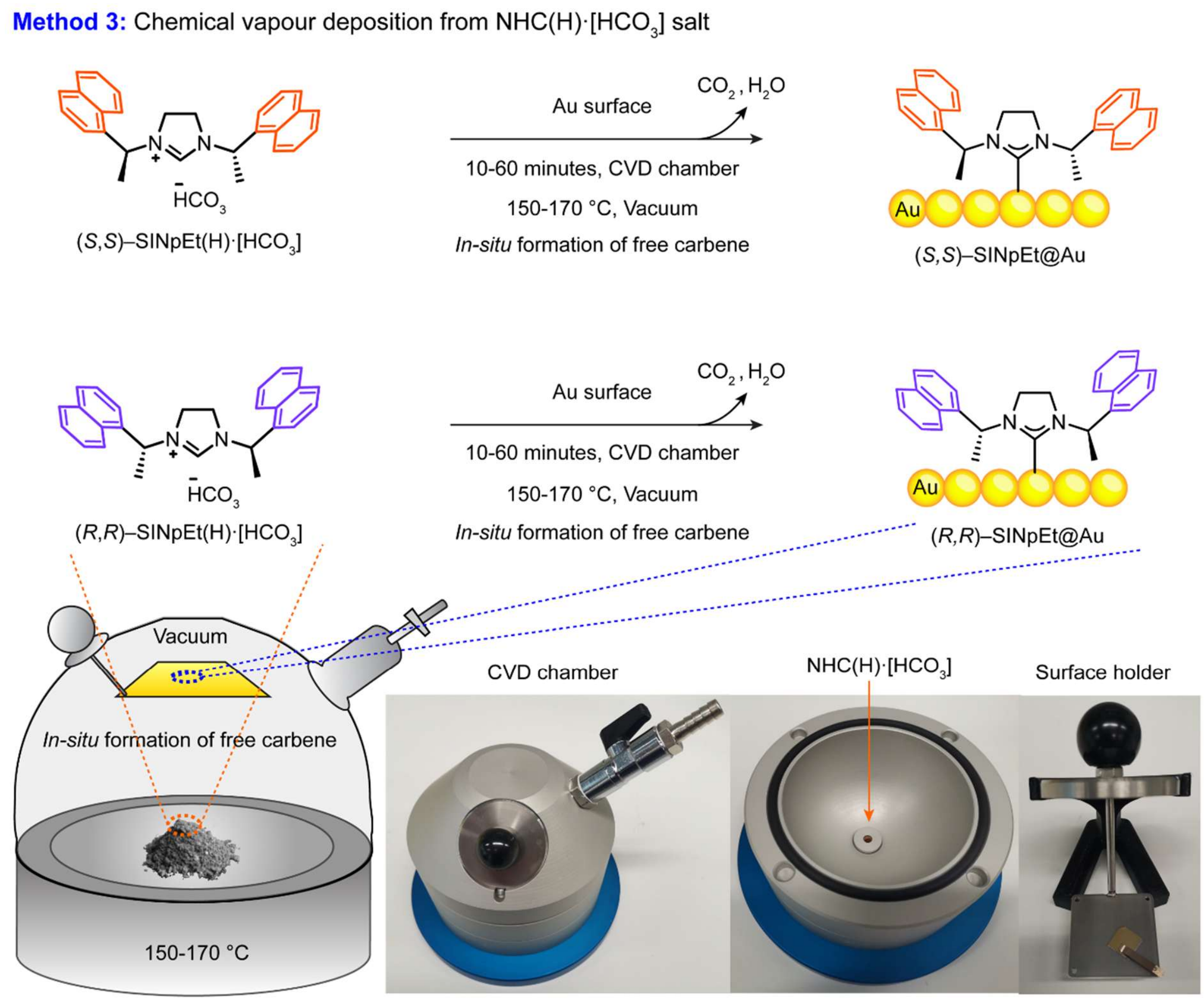

Figure S8: Schematic for the third method used for the fabrication of the (*S*,*S*)–, and (*R*,*R*)–SINpEt@Au thin film: chemical vapour deposition from (*S*,*S*)–, and (*R*,*R*)–SINpEt·[HCO_3] salt.

Section S-3: UV-Vis and CD spectra

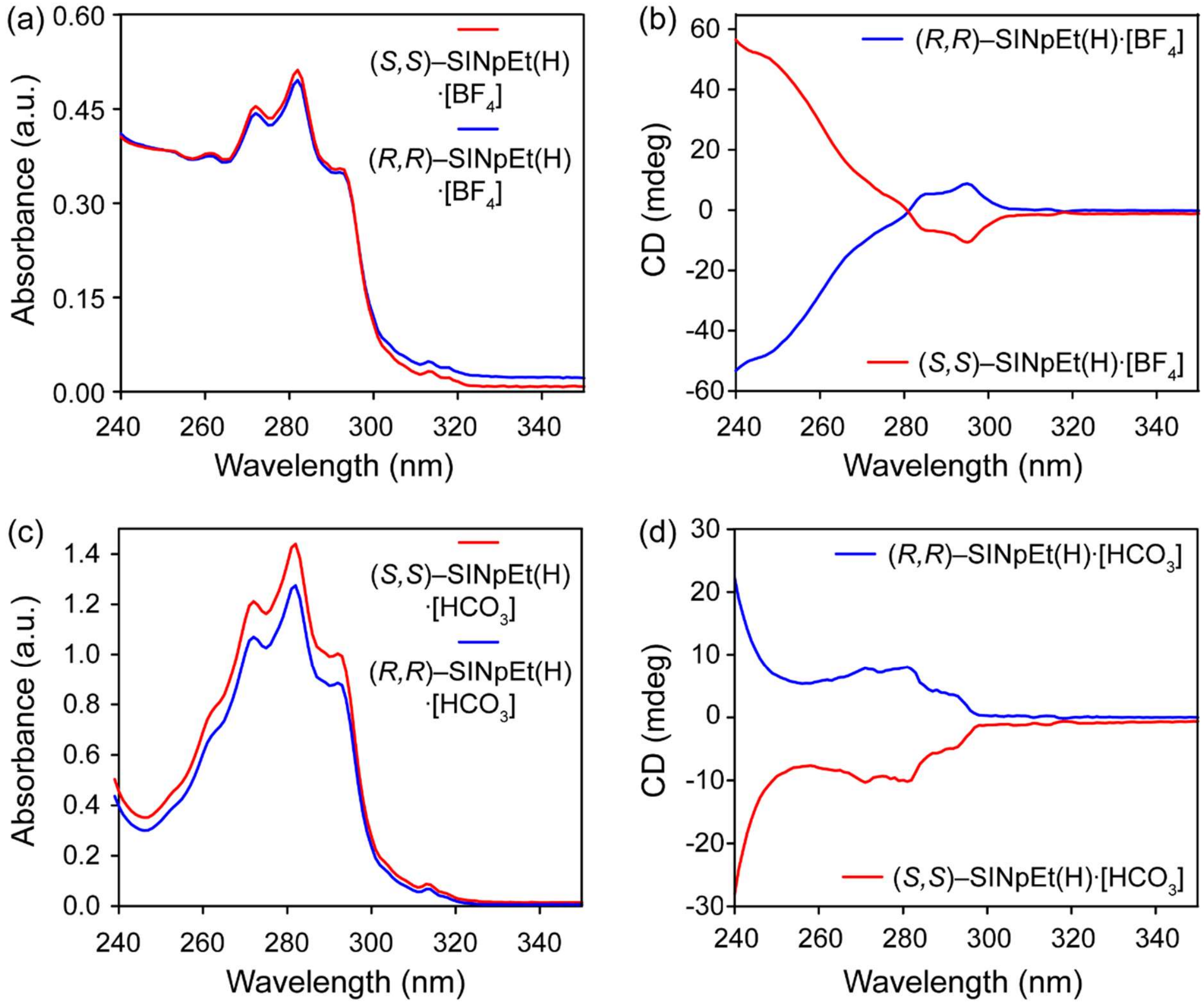

Figure S9: (a) UV-Vis spectra and (b) CD spectra of the ~1 x 10^{-5} M (*S*,*S*)–, and (*R*,*R*)–SINpEt·$[BF_4]$ salt in Methanol. (c) UV-Vis spectra and (b) CD spectra of the ~1 x 10^{-5} M (*S*,*S*)–, and (*R*,*R*)–SINpEt·$[HCO_3]$ salt in Methanol, respectively.

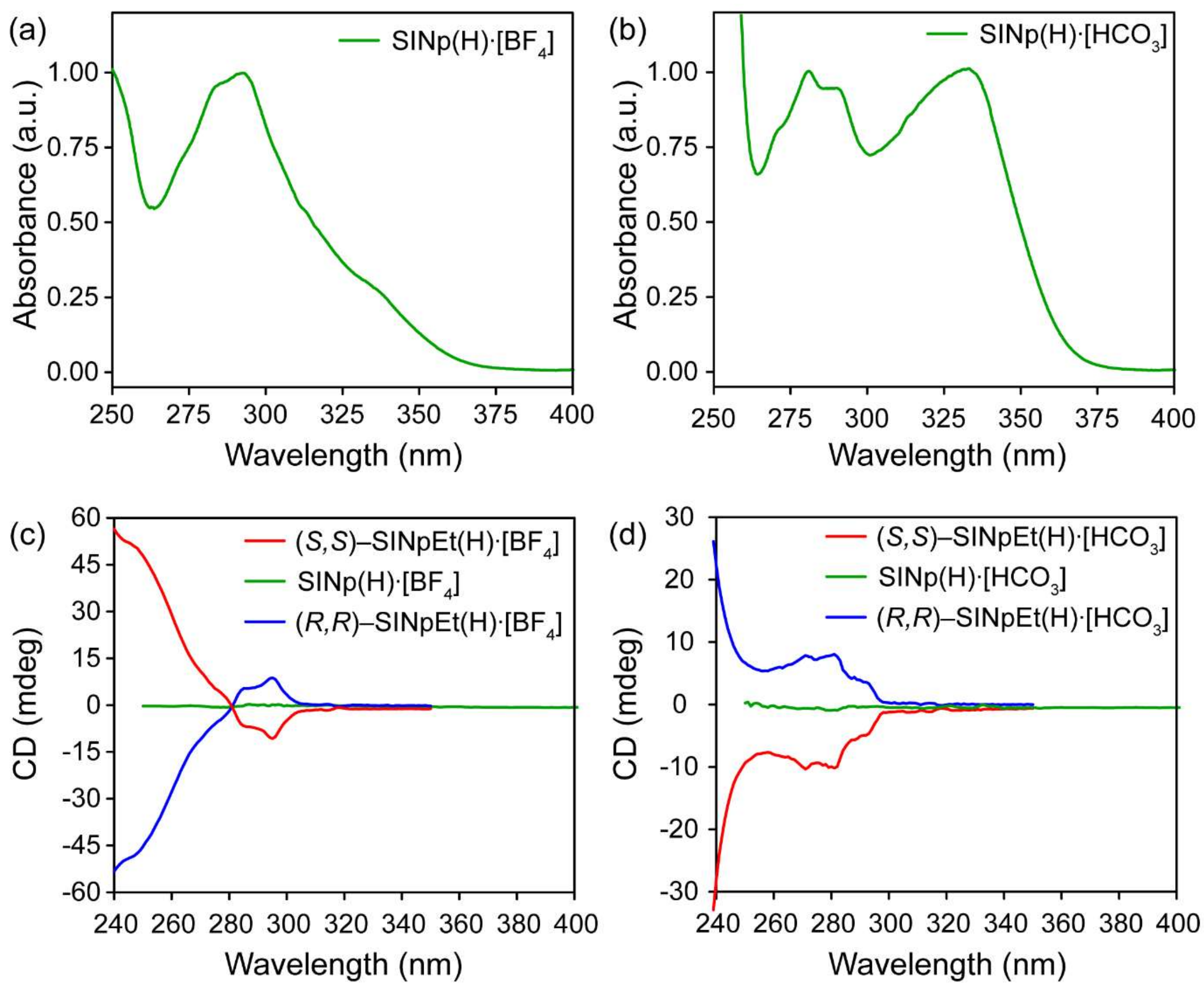

Figure S10: UV-Vis spectra of the ~1 x 10^{-5} M (a) SINp(H)·[BF_4] salt and (b) SINp(H)·[HCO_3] salt in Methanol. CD spectra of the ~1 x 10^{-5} M (c) SINp(H)·[BF_4] salt and (d) SINp·[HCO_3] salt in Methanol, respectively, along with the CD spectra of (*S*,*S*)–SINpEt·[BF_4], (*R*,*R*)–SINpEt·[BF_4], and the (*S*,*S*)–SINpEt·[HCO_3], (*R*,*R*)–SINpEt·[HCO_3] salts, respectively. The CD spectra of both SINp(H)·[BF_4] and SINp(H)·[HCO_3] salt revealed that they are achiral, as expected.

Section S-4: XPS analysis

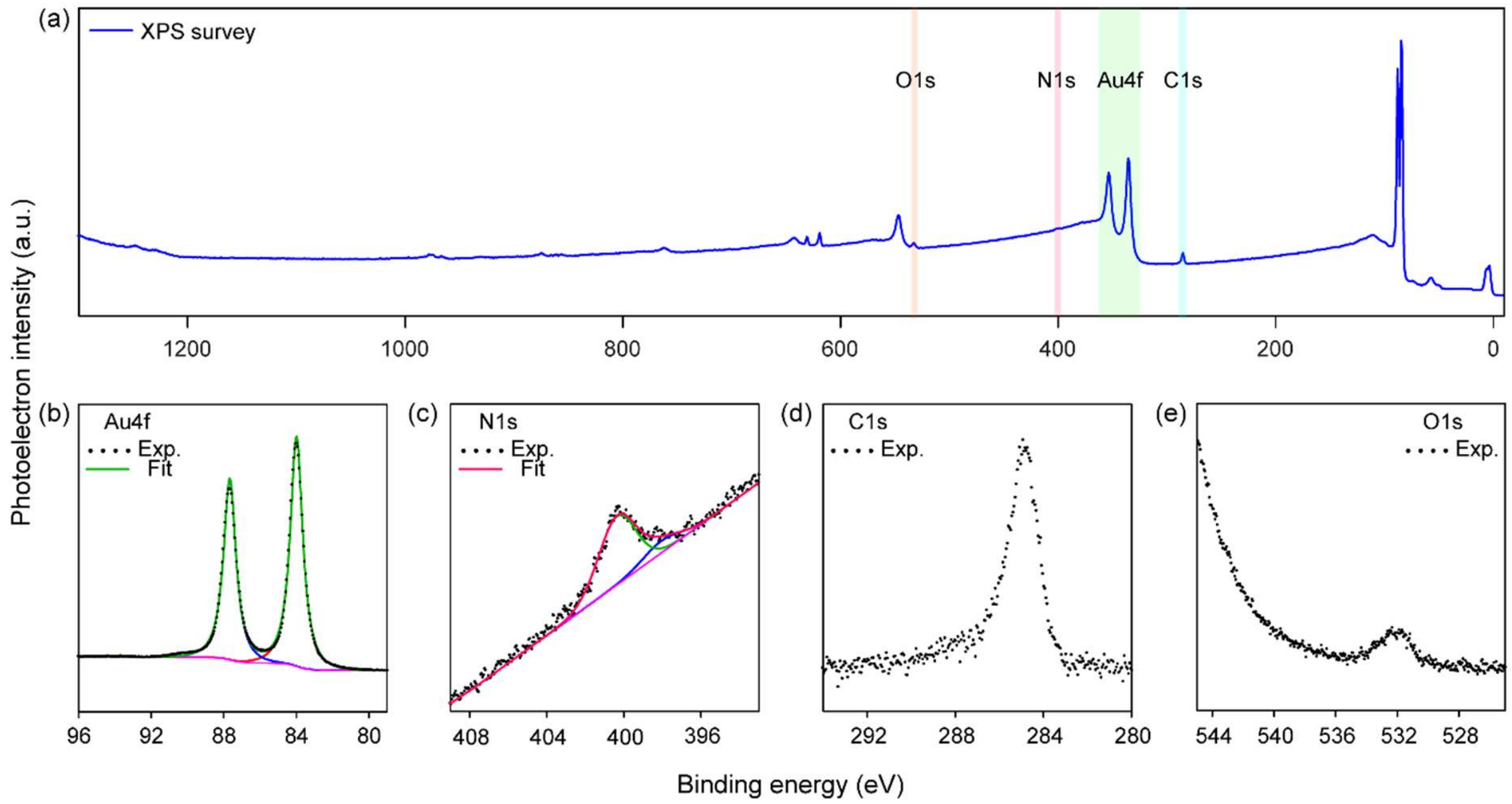

Figure S11: XPS spectra analysis of the (*S*,*S*)–SINpEt@Au-Ni surface, obtained in solution functionalization from NHC(H)·[HCO_3] salt (method 1). (a) XPS survey spectra. High resolution (b) Au 4f (c) N 1s (d) C 1s and (e) O 1s spectra. Herein, the concentration and the deposition time were maintained as 2 mM (in MeOH) and 2 hours, respectively.

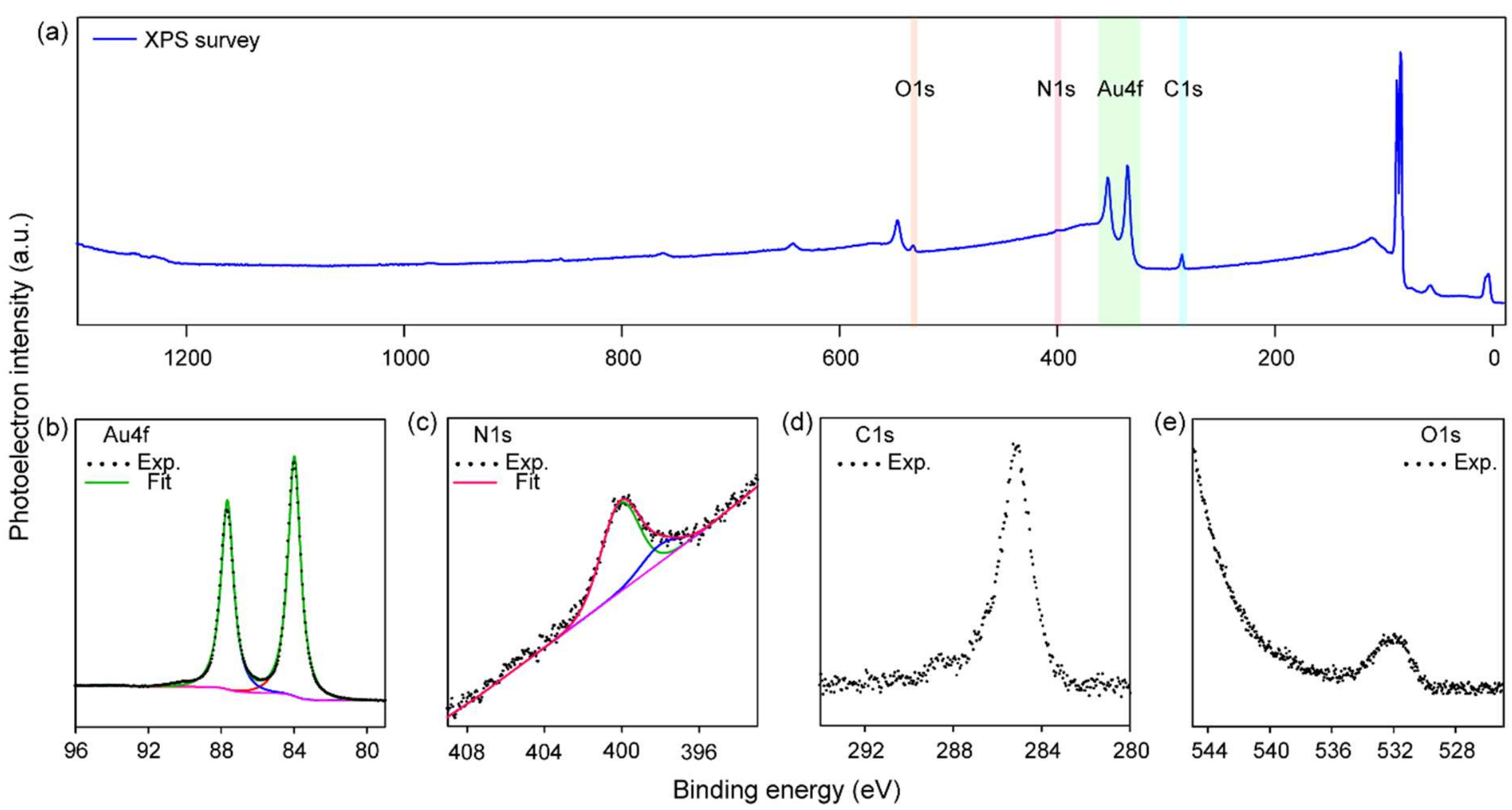

Figure S12: XPS spectra analysis of the (*R*,*R*)–SINpEt@Au-Ni surface, obtained in solution functionalization from NHC(H)·[HCO_3] salt (method 1). (a) XPS survey spectra. High resolution (b)

Au 4f (c) N 1s (d) C 1s and (e) O 1s spectra. Herein, the concentration and the deposition time were maintained as 2 mM (in MeOH) and 2 hours, respectively.

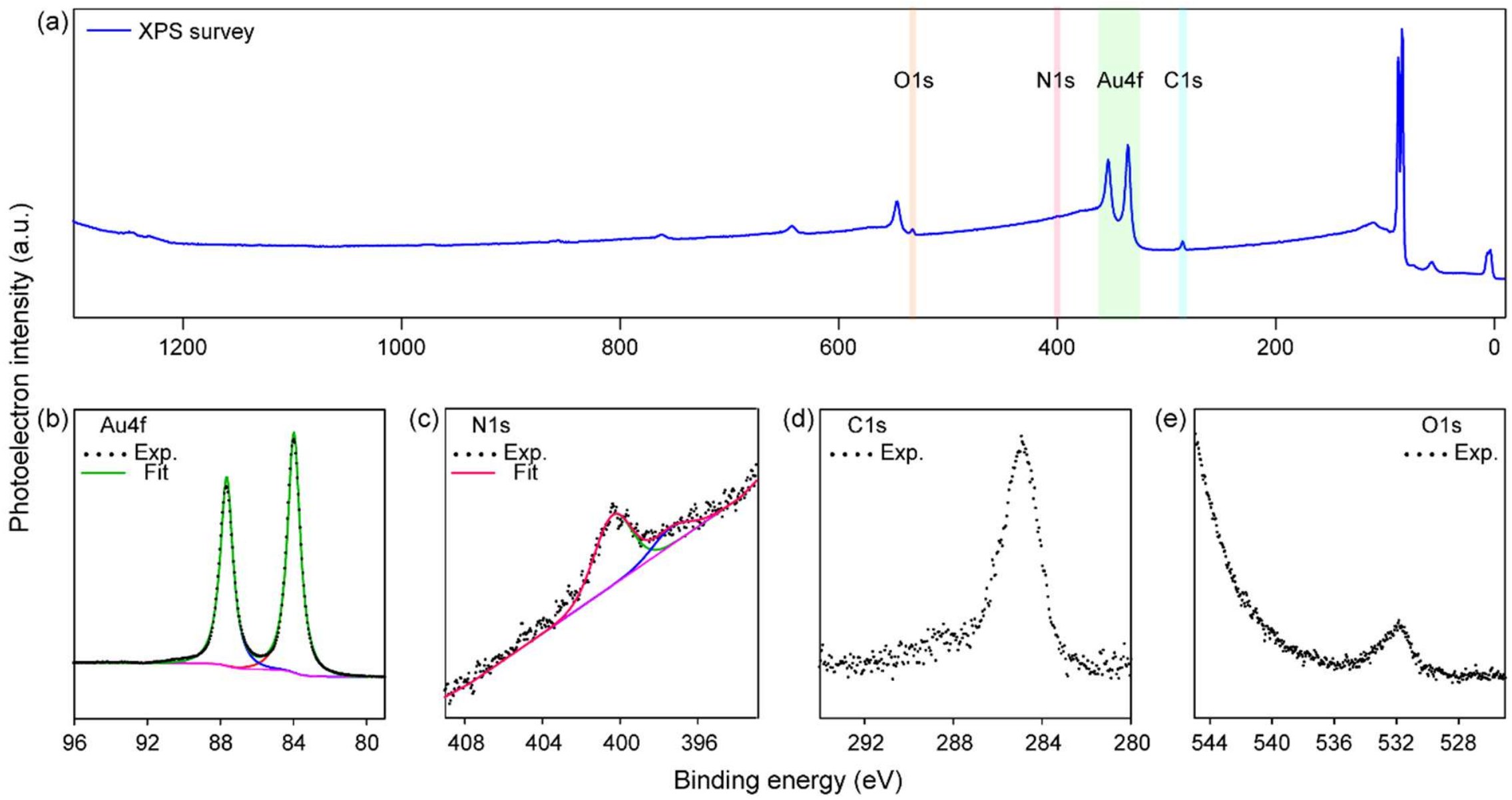

Figure S13: XPS spectra analysis of the (*S*,*S*)–SINpEt@Au-Ni surface, obtained in free carbene functionalization from NHC(H)·[BF_4] salt (method 2). (a) XPS survey spectra. High resolution (b) Au 4f (c) N 1s (d) C 1s and (e) O 1s spectra. Herein, the concentration and the deposition time were maintained as ~2 mM (in toluene) and 2 hours, respectively.

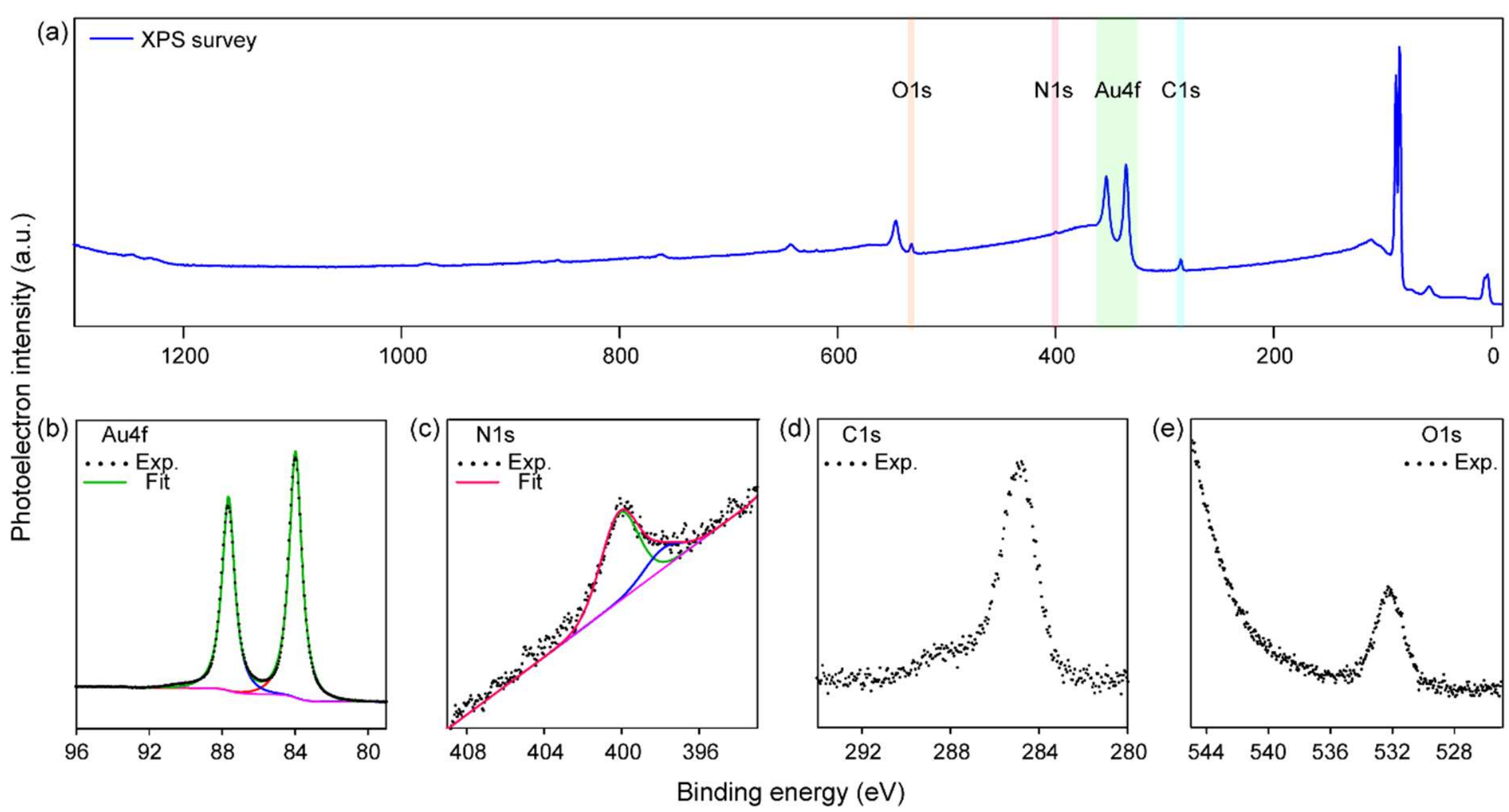

Figure S14: XPS spectra analysis of the (*R*,*R*)–SINpEt@Au-Ni surface, obtained in free carbene functionalization from NHC(H)·[BF_4] salt (method 2). (a) XPS survey spectra. High resolution (b) Au

4f (c) N 1s (d) C 1s and (e) O 1s spectra. Herein, the concentration and the deposition time were maintained as ~2 mM (in toluene) and 2 hours, respectively.

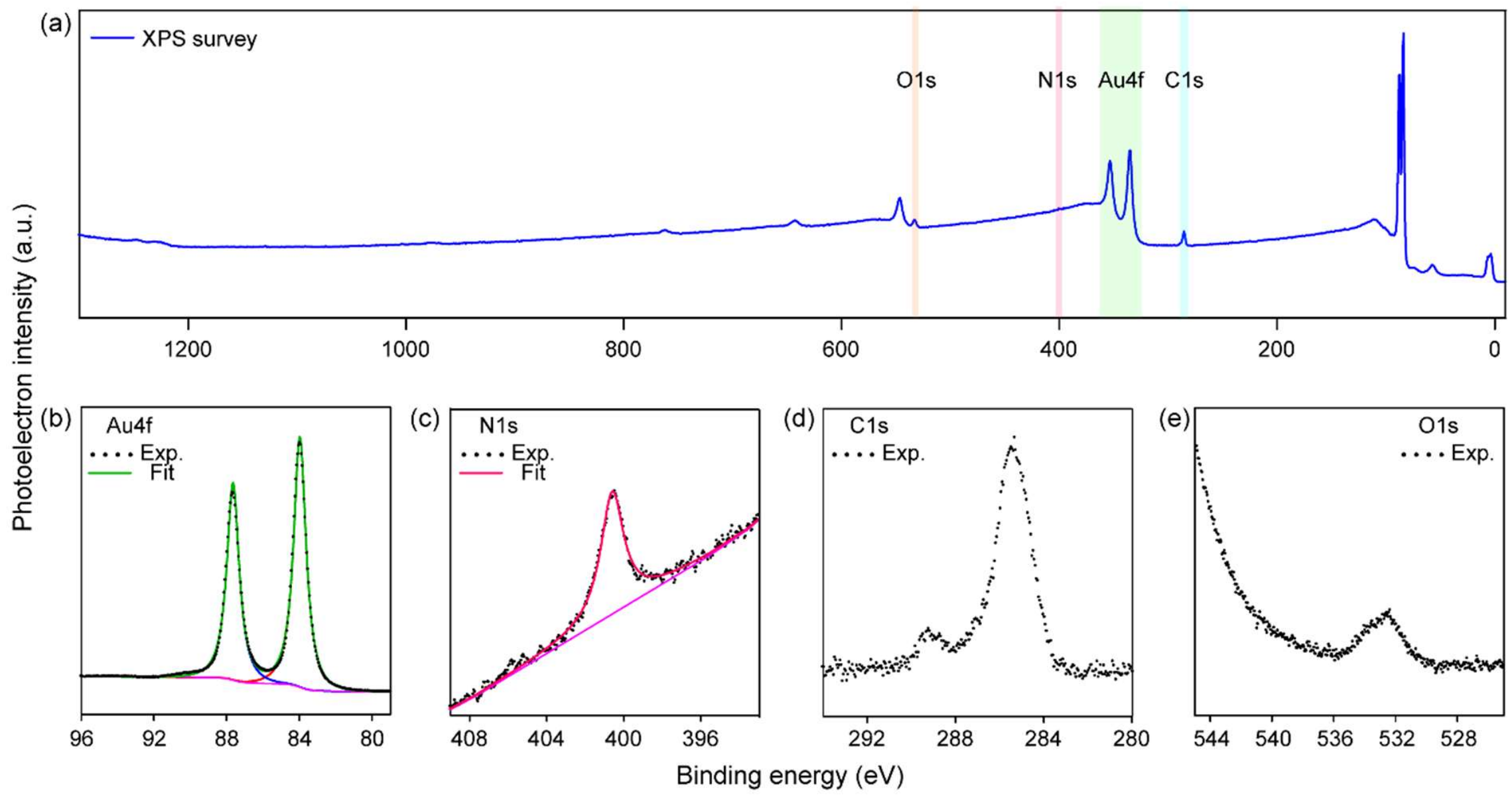

Figure S15: XPS spectra analysis of the (*S*,*S*)–SINpEt@Au-Ni surface obtained in chemical vapour deposition from NHC(H)·[HCO_3] salt (method 3). (a) XPS survey spectra. High resolution (b) Au 4f (c) N 1s (d) C 1s and (e) O 1s spectra. Herein, the deposition time was maintained at 30 minutes during the chemical vapour deposition.

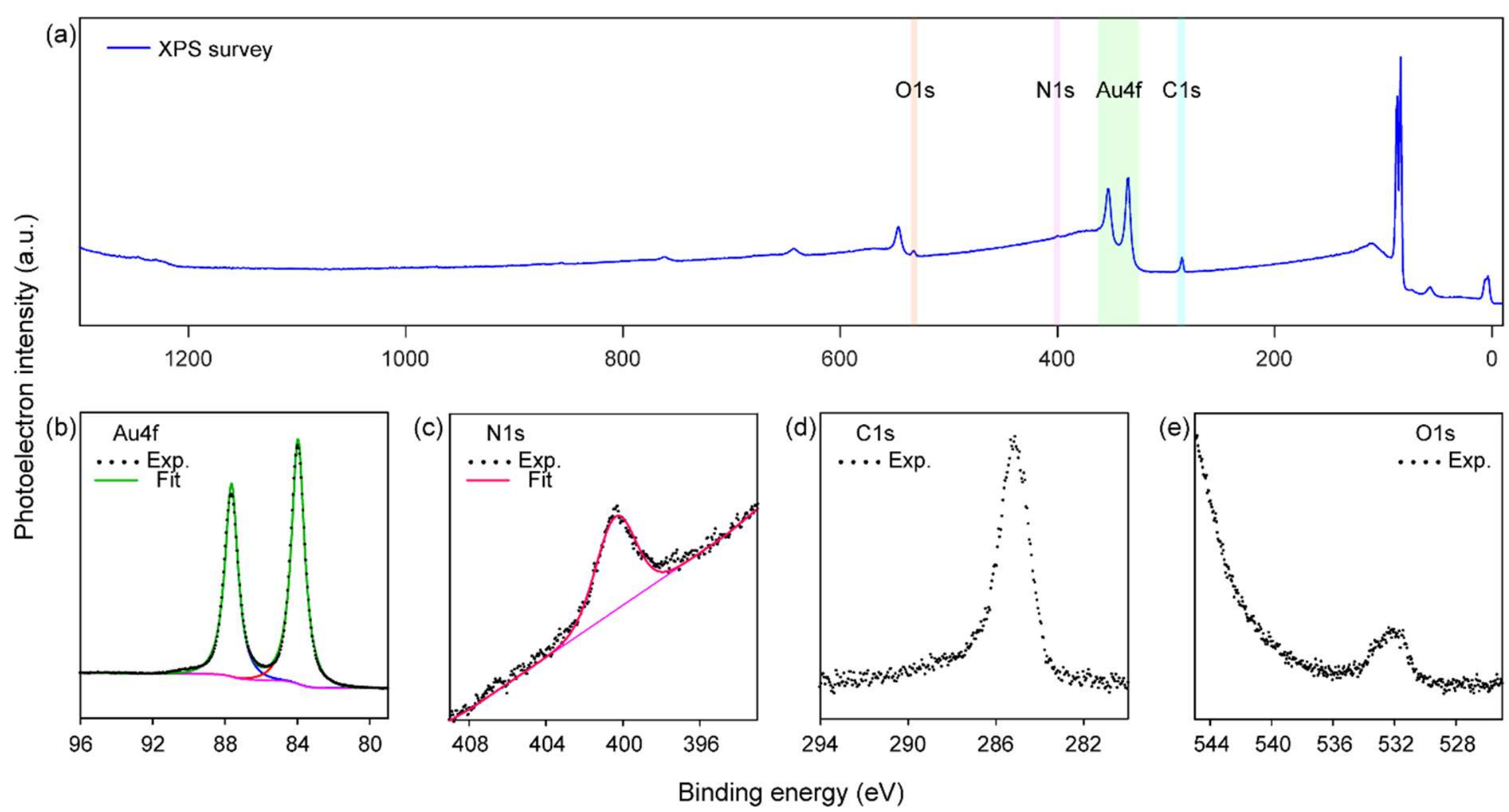

Figure S16: XPS spectra analysis of the (*R*,*R*)–SINpEt@Au-Ni surface obtained in chemical vapour deposition from NHC(H)·[HCO_3] salt (method 3). (a) XPS survey spectra. High resolution (b) Au 4f

(c) N 1s (d) C 1s and (e) O 1s spectra. Herein, the deposition time was maintained at 30 minutes during the chemical vapour deposition.

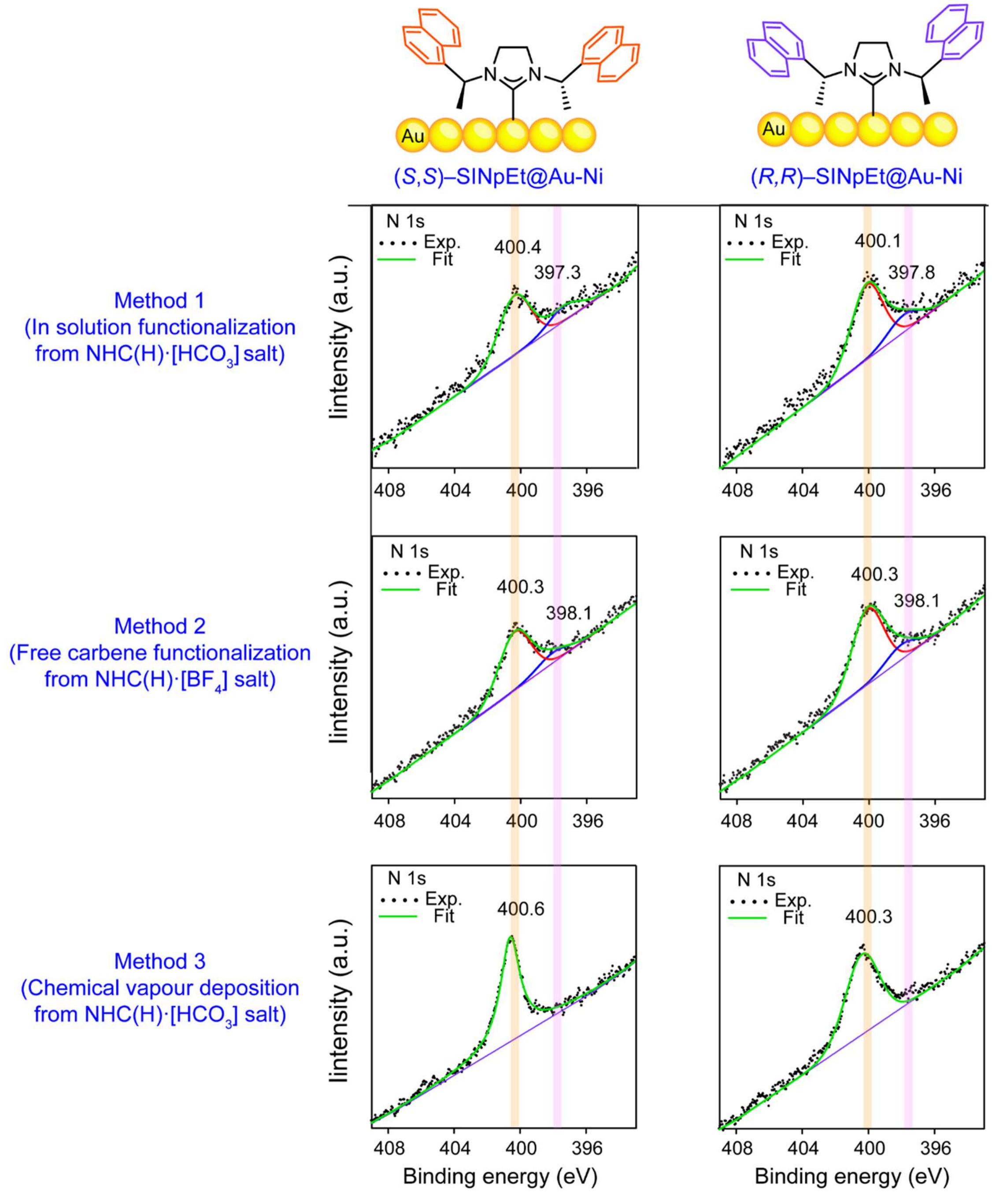

Figure S17: Comparison of the XP N 1s spectra of the (*S*,*S*)–, and (*R*,*R*)–SINpEt functionalized Au-Ni surfaces fabricated in three different methods, respectively.

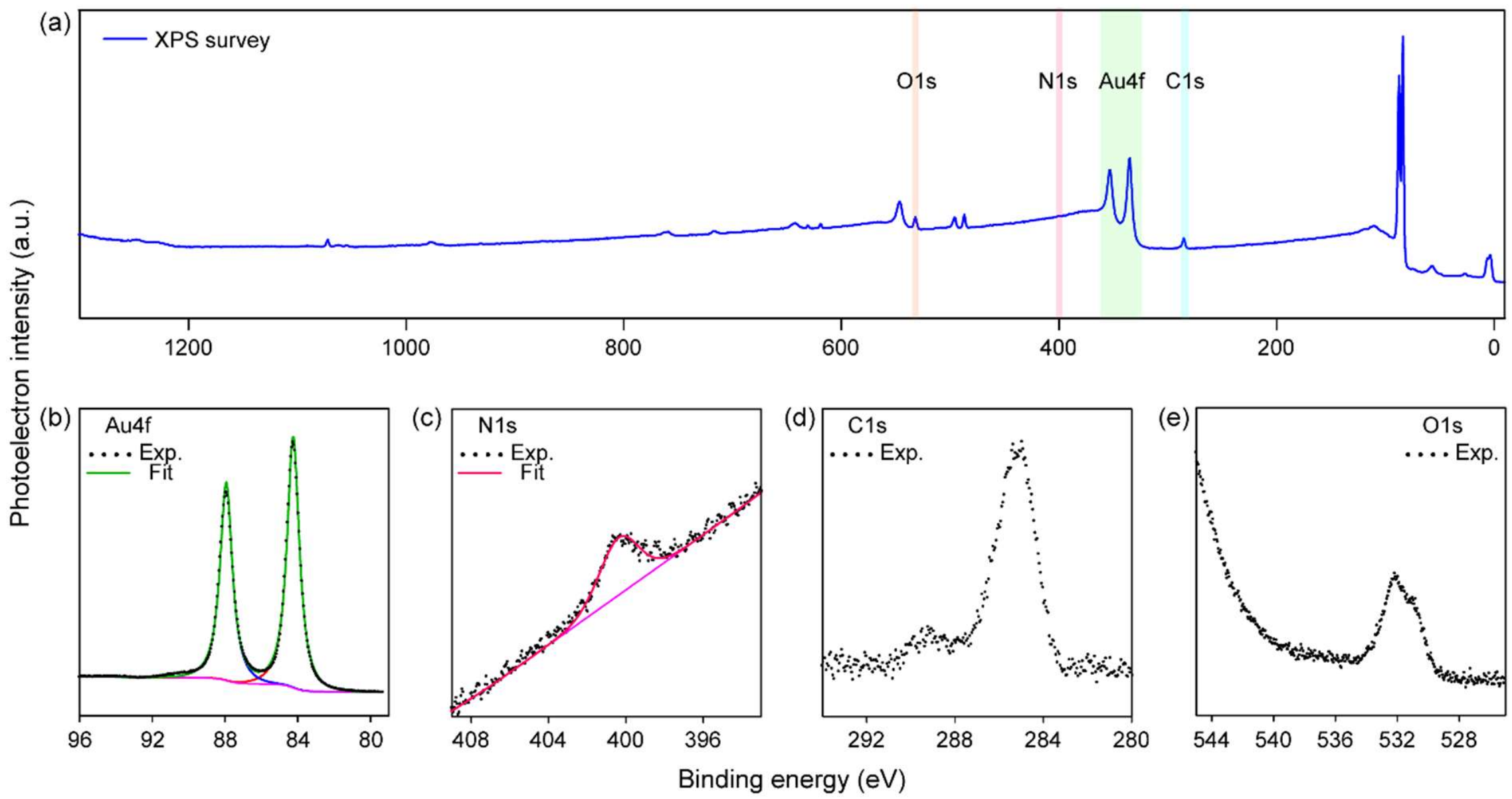

Figure S18: XPS spectra analysis of the (*S*,*S*)–SINpEt@Au surface obtained in chemical vapour deposition from (*S*,*S*)–SINpEt(H)·[HCO_3]. (a) XPS survey spectra. High resolution (b) Au 4f (c) N 1s (d) C 1s and (e) O 1s spectra. Herein, the deposition time was maintained at 10 minutes during the chemical vapour deposition.

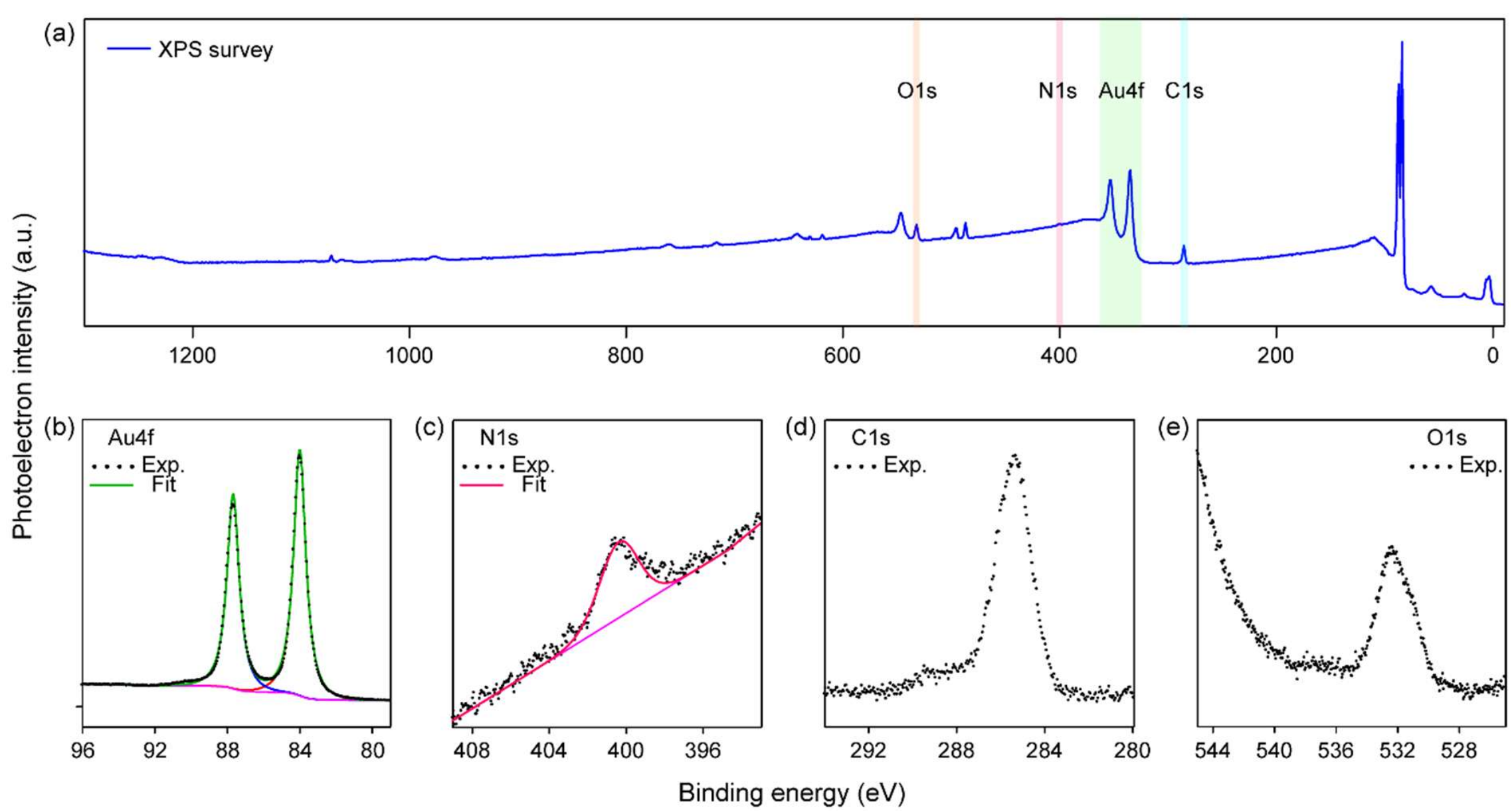

Figure S19: XPS spectra analysis of the (*S*,*S*)–SINpEt@Au surface obtained in chemical vapour deposition from (*S*,*S*)–SINpEt(H)·[HCO_3]. (a) XPS survey spectra. High resolution (b) Au 4f (c) N 1s (d) C 1s and (e) O 1s spectra. Herein, the deposition time was maintained at 20 minutes during the chemical vapour deposition.

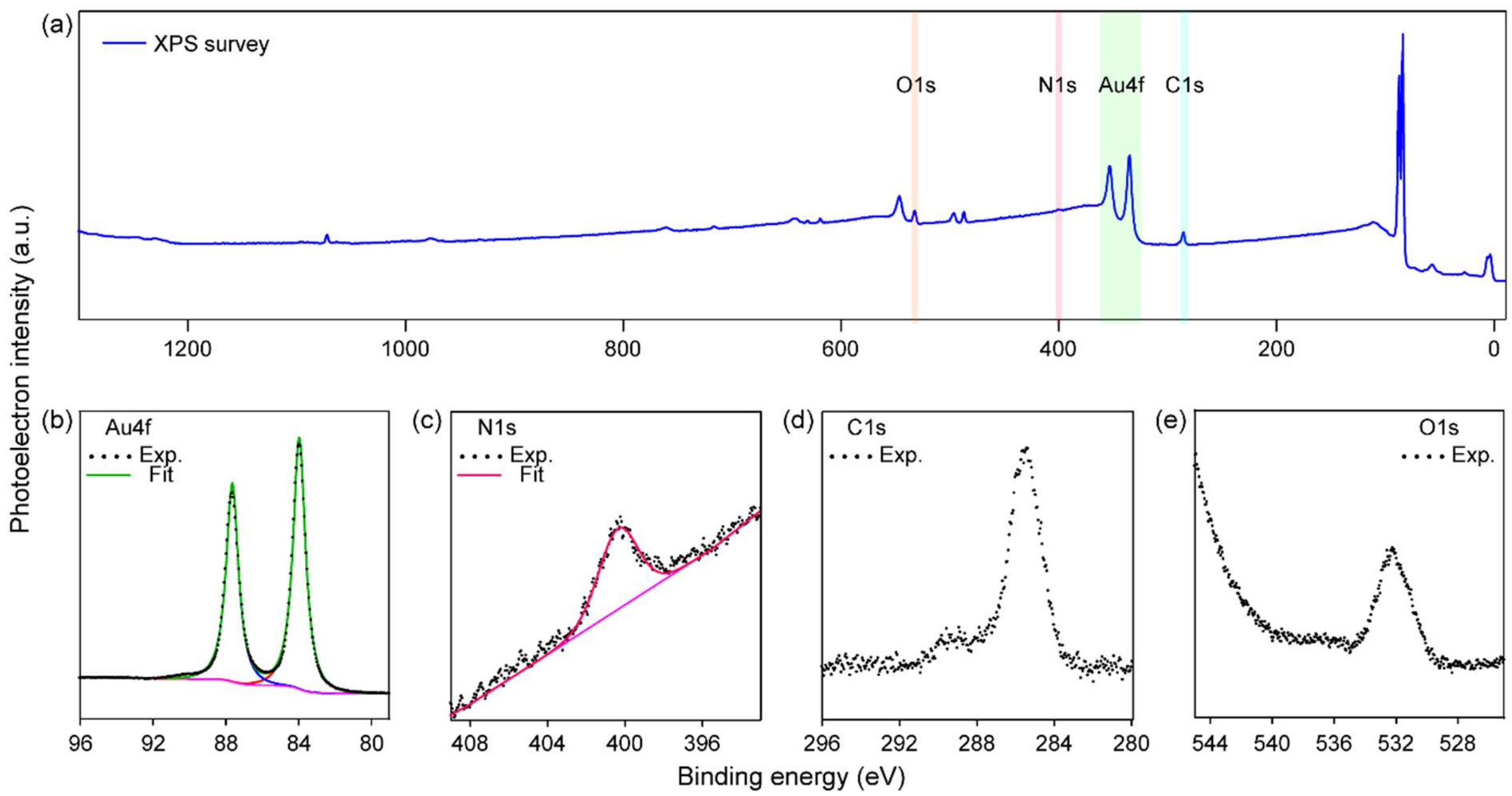

Figure S20: XPS spectra analysis of the (*S*,*S*)–SINpEt@Au surface obtained in chemical vapour deposition from (*S*,*S*)–SINpEt(H)·[HCO_3]. (a) XPS survey spectra. High resolution (b) Au 4f (c) N 1s (d) C 1s and (e) O 1s spectra. Herein, the deposition time was maintained at 30 minutes during the chemical vapour deposition.

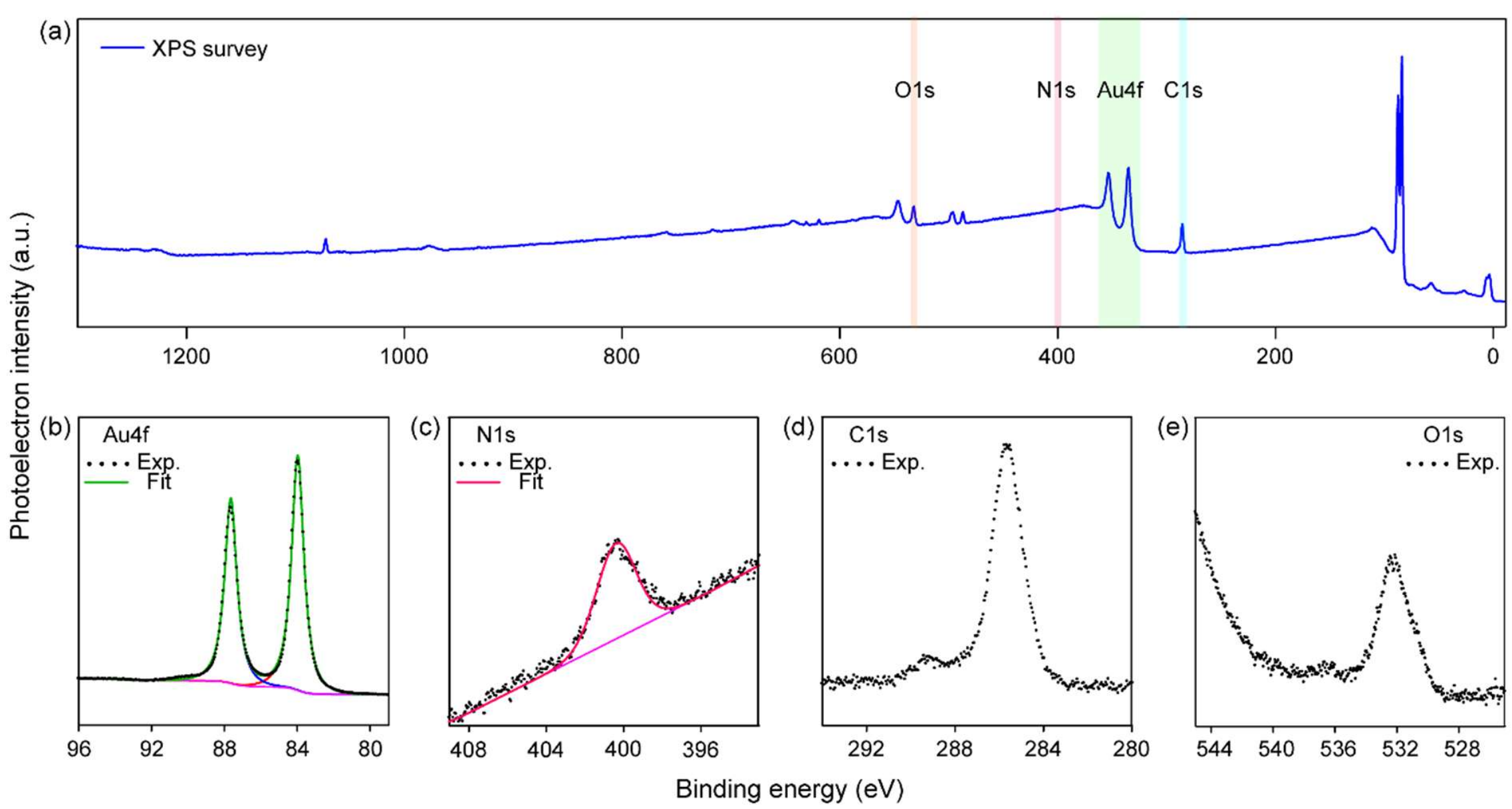

Figure S21: XPS spectra analysis of the (*S*,*S*)–SINpEt@Au surface obtained in chemical vapour deposition from (*S*,*S*)–SINpEt(H)·[HCO_3]. (a) XPS survey spectra. High resolution (b) Au 4f (c) N 1s (d) C 1s and (e) O 1s spectra. Herein, the deposition time was maintained at 60 minutes during the chemical vapour deposition.

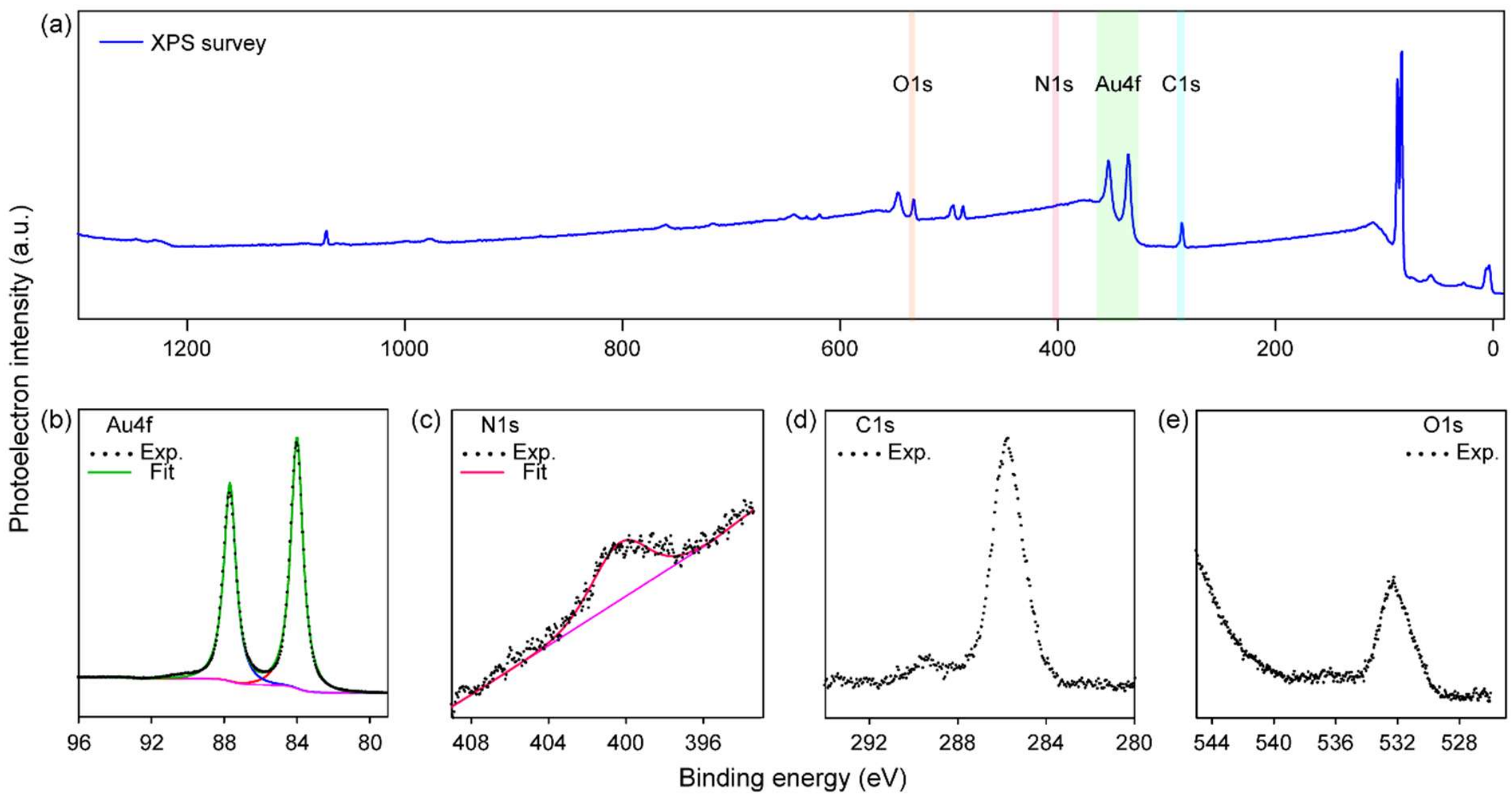

Figure S22: XPS spectra analysis of the (*R*,*R*)–SINpEt@Au surface obtained in chemical vapour deposition from (*R*,*R*)–SINpEt(H)·[HCO_3]. (a) XPS survey spectra. High resolution (b) Au 4f (c) N 1s (d) C 1s and (e) O 1s spectra. Herein, the deposition time was maintained at 10 minutes during the chemical vapour deposition.

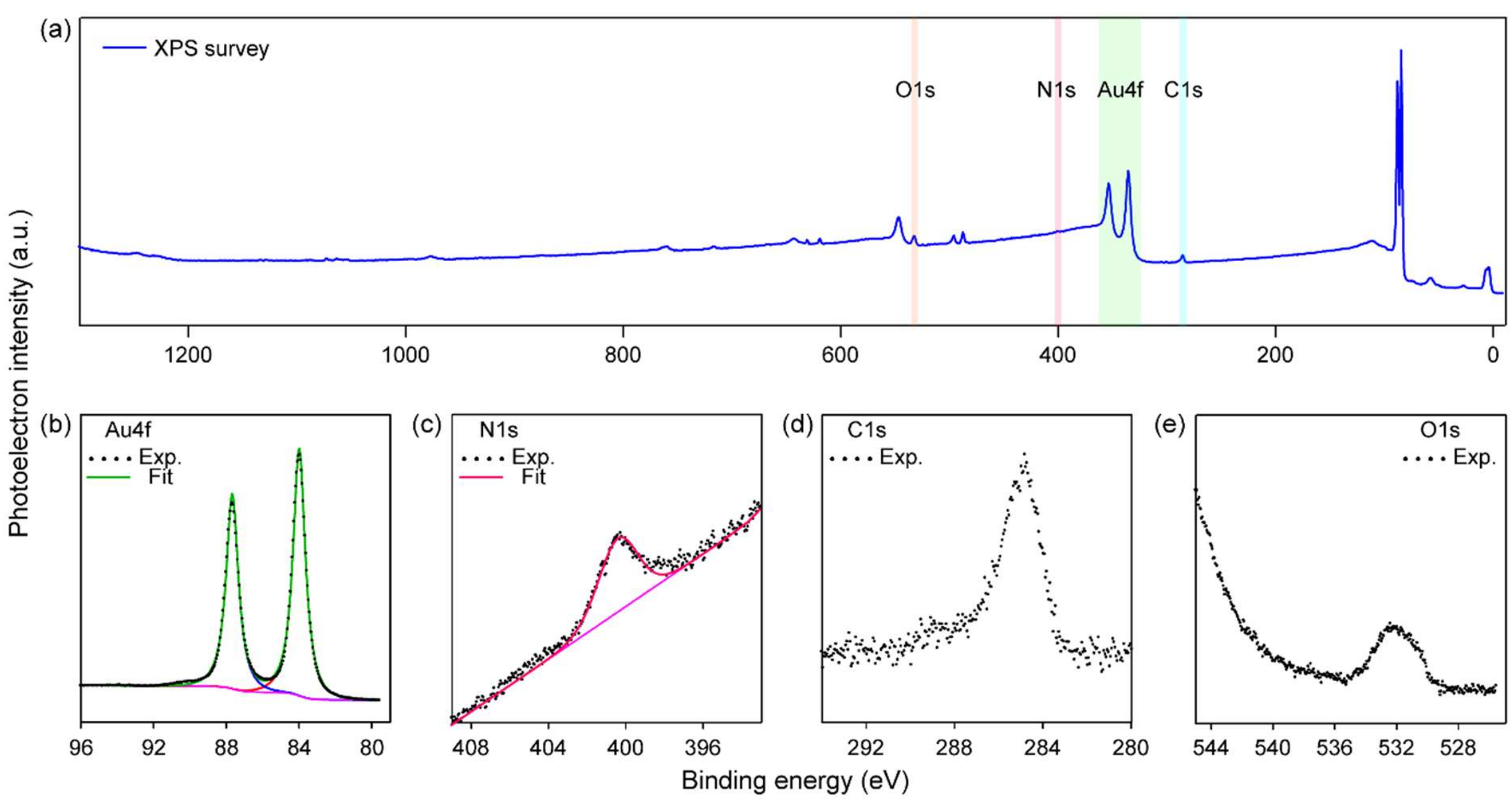

Figure S23: XPS spectra analysis of the (*R*,*R*)–SINpEt@Au surface obtained in chemical vapour deposition from (*R*,*R*)–SINpEt(H)·[HCO_3]. (a) XPS survey spectra. High resolution (b) Au 4f (c) N 1s (d) C 1s and (e) O 1s spectra. Herein, the deposition time was maintained at 20 minutes during the chemical vapour deposition.

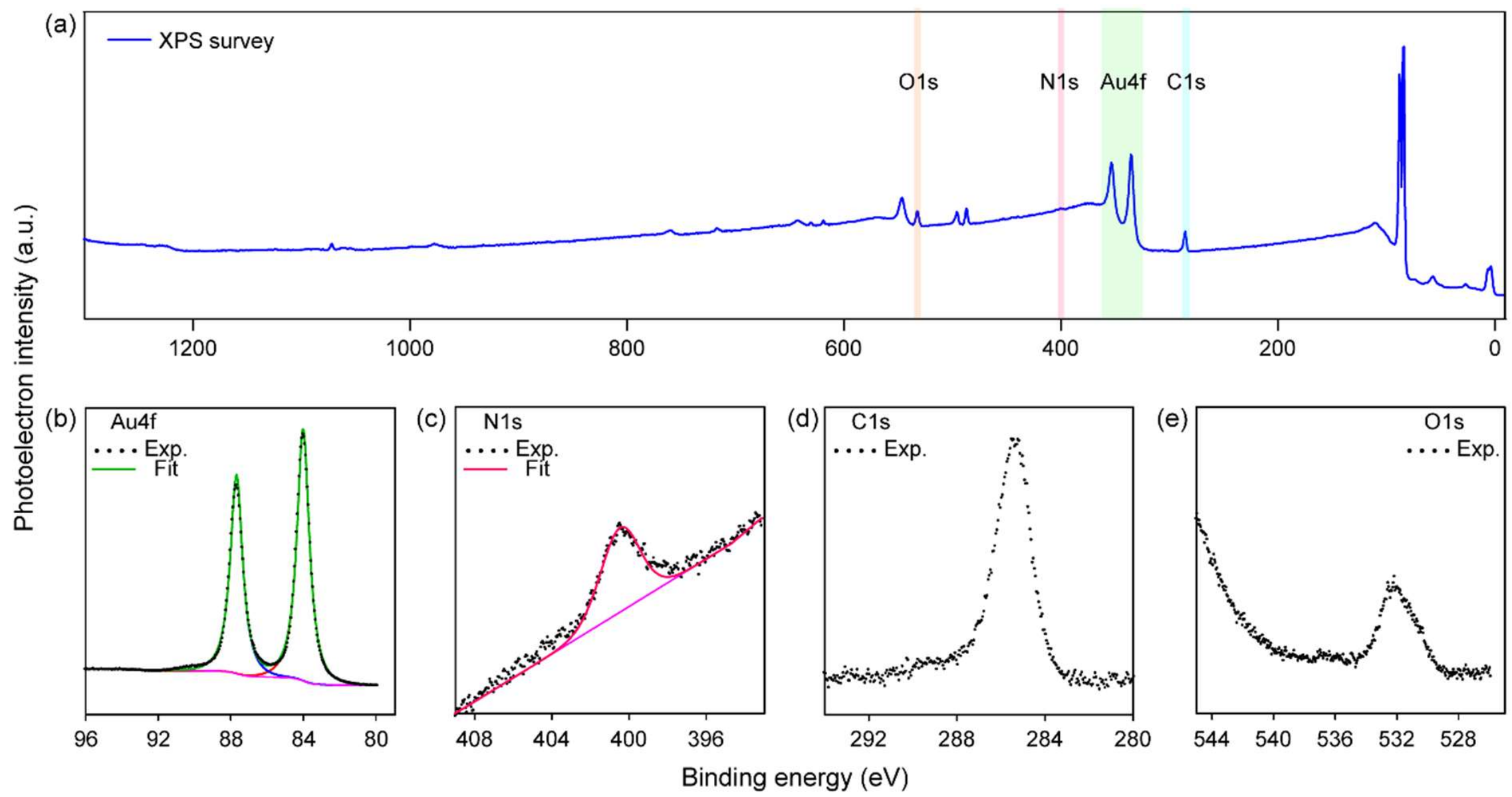

Figure S24: XPS spectra analysis of the (*R*,*R*)–SINpEt@Au surface obtained in chemical vapour deposition from (*R*,*R*)–SINpEt(H)·[HCO_3]. (a) XPS survey spectra. High resolution (b) Au 4f (c) N 1s (d) C 1s and (e) O 1s spectra. Herein, the deposition time was maintained at 30 minutes during the chemical vapour deposition.

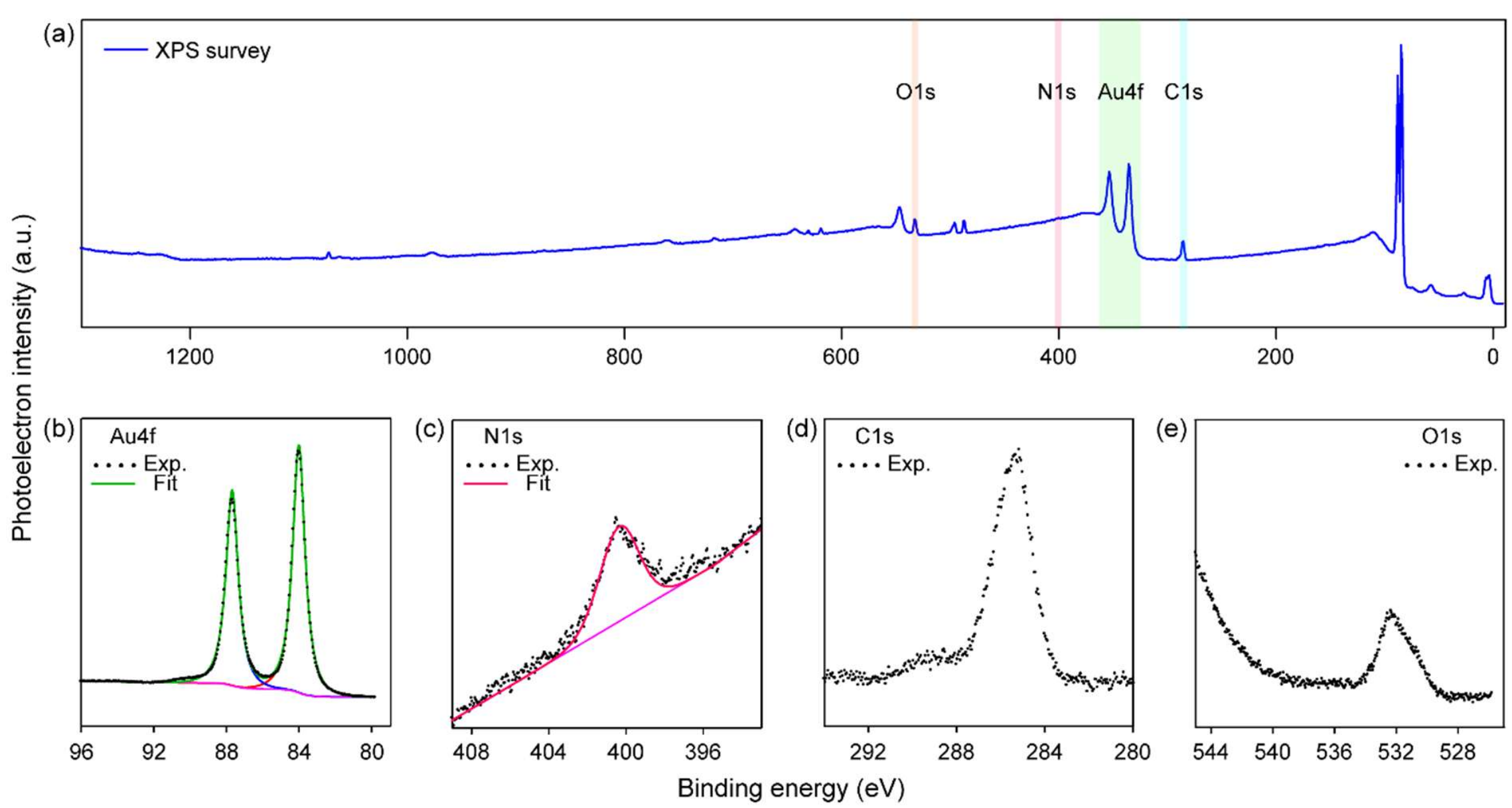

Figure S25: (XPS spectra analysis of the (*R*,*R*)–SINpEt@Au surface obtained in chemical vapour deposition from (*R*,*R*)–SINpEt(H)·[HCO_3]. (a) XPS survey spectra. High resolution (b) Au 4f (c) N 1s (d) C 1s and (e) O 1s spectra. Herein, the deposition time was maintained at 60 minutes during the chemical vapour deposition.

Section S-5: Molecular coverage calculations

X-ray Photoelectron Spectroscopy (XPS) depth profiling maps elemental and chemical state composition below the top surface. It evaluates molecular coverage and layer thickness by measuring changes in concentration against depth, utilizing either destructive sputtering or non-destructive angular analysis. We have followed the destructive sputtering pathway for depth profiling to determine the molecular surface coverage of the surfaces of the (*S,S*)–SINpEt@Au and (*R,R*)–SINpEt@Au surfaces at different deposition times. For estimating the coverage, we neglected any attenuation of the Al K_α radiation ($h\nu = 1486.7\ eV$) due to passing through the sample, as the attenuation length of X-ray photons exceeds the attenuation length of photoelectrons by several orders of magnitude. The short attenuation length of photoelectrons was the reason for the inherent surface sensitivity. Here, we consider the gold Au $4f_{7/2}$ photoelectrons to be attenuated by other gold atoms and by the organic NHC layer. We also considered the nitrogen N 1s photoelectrons to be attenuated by the organic NHC layer.[5]

The inelastic mean free path (IMFP, λ) describes the average distance an electron travels before losing significant energy. IMPF of a photoelectron emitted by an atomic orbital *i,* generally depends on its kinetic energy *KE(i)* and on the material *j* it travels through. Via an experimental correlation, λ_i^j may be approximated as follows using the material-dependent factor *B(j)*.[6,7]

$$\lambda_i^j = B(j)\sqrt{KE(i)} \qquad (1)$$

The resulting IMFP values λ_i^j, relevant for this work, is summarized in Table S1.

Table S1: The material parameters *B(j)*, the kinetic energies *KE(i)* and the IMFPs λ_i^j.

	$B(j)\ [nm\ eV^{-0.5}]$	$KE(i)\ [eV]$	$\lambda_i^j\ [nm]$
λ_{Au4f7}^{Au}	0.054	1402.7	2.02
λ_{Au4f7}^{org}	0.087	1402.7	3.26
λ_{N1s}^{org}	0.087	1086.3	2.87

According to the Beer-Lambert extinction law, the thickness *d* of the organic top layer can be estimated by comparison of the Au $4f_{7/2}$ signal intensity before (I_{Au4f7}) and after (I_{Au4f7}^0) ion-gun sputtering.[6]

$$I_{Au4f7} = I^0_{Au4f7}\, e^{\frac{-d}{\lambda^{org}_{Au4f7}}} \quad (2)$$

$$d = \lambda^{org}_{Au4f7}\, ln \frac{I^0_{Au4f7}}{I_{Au4f7}} \quad (3)$$

Based on this thickness, the photoelectron attenuation in the NHC layer and, therefore, the coverage can be assessed in the next step. XPS data were corrected for photoionization cross-section and detector sensitivity, yielding relative signal intensities.

Assessing the attenuation of Au $4f_{7/2}$ photoelectrons by gold atoms, Marder *et al.* describe the NHC coverage on gold as:[8]

$$\frac{n_{NHC}}{A} = \frac{1}{2}\frac{I^{rel}_{N1s}}{I^{rel}_{Au4f7}}\, \lambda^{Au}_{Au4f7}\, \frac{n_{Au}}{V} \quad (4)$$

Here, the coverage is given as the number of NHCs n_{NHC} per surface area A and directly proportional to the XPS signal ratio between nitrogen and gold. The atomic density of gold $\frac{n_{Au}}{V}$ is known to be $\frac{0.059}{Å^3}\ or\ \frac{59}{nm^3}$..

When implying the attenuation of Au $4f_{7/2}$ photoelectrons by the organic NHC layer of the thickness d, I^{rel}_{Au4f7} extends by one decay factor, resulting in:

$$\frac{n_{NHC}}{A} = \frac{1}{2}\frac{I^{rel}_{N1s}}{I^{rel}_{Au4f7}}\, \lambda^{Au}_{Au4f7}\, \frac{n_{Au}}{V} \times e^{\frac{-d}{\lambda^{org}_{Au4f7}}} \quad (5)$$

When applying equation 2 in equation 5 (ignoring the attenuation of N 1*s* electrons by the organic NHC layer of the thickness *d)*:

$$\frac{n_{NHC}}{A} = \frac{1}{2}\frac{I^{rel}_{N1s}}{I^{rel}_{Au4f7}}\, \lambda^{Au}_{Au4f7}\, \frac{n_{Au}}{V} \times \frac{I_{Au4f7}}{I^0_{Au4f7}} \quad (6)$$

Table S2: The relative signal intensities of Au $4f_{7/2}$, N 1*s* (normalized to add up to 100 %).

(*S*,*S*)–SINpEt @Au (10 minutes)	(*S*,*S*)–SINpEt @Au (20 minutes)	(*S*,*S*)–SINpEt @Au (30 minutes)	(*S*,*S*)–SINpEt @Au (60 minutes)	(*R*,*R*)–SINpEt @Au (10 minutes)	(*R*,*R*)–SINpEt @Au (20 minutes)	(*R*,*R*)–SINpEt @Au (30 minutes)	(*R*,*R*)–SINpEt @Au (60 minutes)

I^{rel}_{Au4f7}	96.59 %	95.13 %	95.24 %	92.75 %	96.78 %	95.94 %	93.85 %	93.63 %
I^{rel}_{N1s}	3.41 %	4.87 %	4.76 %	7.25 %	3.22 %	4.06 %	6.15 %	6.37 %

Table S3: The relative signal intensities of I_{Au4f7} and I^0_{Au4f7} for different surfaces.

	(*S*,*S*)–SINpEt @Au (10 minutes)	(*S*,*S*)–SINpEt @Au (20 minutes)	(*S*,*S*)–SINpEt @Au (30 minutes)	(*S*,*S*)–SINpEt @Au (60 minutes)	(*R*,*R*)–SINpEt @Au (10 minutes)	(*R*,*R*)–SINpEt @Au (20 minutes)	(*R*,*R*)–SINpEt @Au (30 minutes)	(*R*,*R*)–SINpEt @Au (60 minutes)
$\frac{I_{Au4f7}}{I^0_{Au4f7}}$	0.41	0.35	0.38	0.29	0.41	0.45	0.34	0.33

As the measured XPS signal intensities contain statistical errors, these might be propagated towards uncertainties of the obtained coverages. However, attenuation lengths resembled a major systematic uncertainty of this coverage calculation, underlining its approximate character. For applying more complex and accurate attenuation length models, too many parameters of the NHC layer (e.g., asymmetry parameter, density) are unknown. Exemplary for other NHCs on gold, some literature substituted the IMFP $\lambda^{Au}_{Au4f7} = 2.02\ nm$ with an effective attenuation length (EAL) of $1.62\ nm$ and $\lambda^{Org}_{Au4f7} = 3.26\ nm$ with an EAL of $3.89\ nm$.[5] This said, the excellent agreement between the two coverage estimations based on N 1*s* confirmed the assumption of iron atoms sitting at the outer edge of the NHC SAM. The obtained coverage values were also in good agreement with literature reports of similar NHC monolayers, confirming their dense monolayer character.[5,9]

Table S4: Coverage and molecular footprint of (*S*,*S*)–SINpEt and (*R*,*R*)–SINpEt on Au surface at different deposition times based on XPS.

Deposition time (minutes)	Coverage (molecules/nm^2) of (*S*,*S*)–SINpEt on Au surface	Coverage (molecules/nm^2) of (*R*,*R*)–SINpEt on Au surface
10	0.69±0.029	0.65±0.034

20	0.86±0.016	0.91±0.020
30	0.91±0.017	1.06±0.016
60	1.08±0.022	1.07±0.015

Section S-6: ToF-SIMS analysis

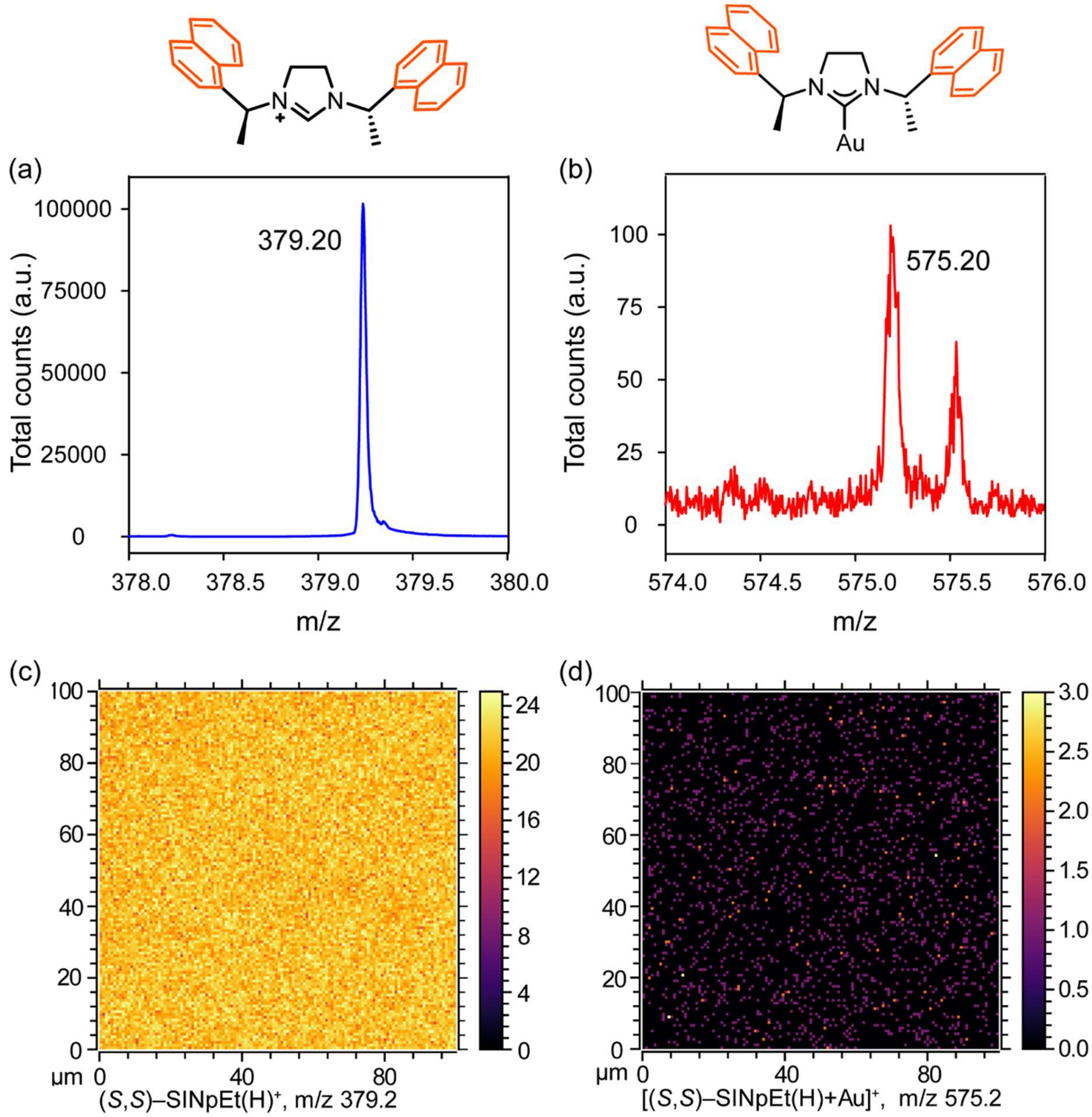

Figure S26. (a-b) ToF-SIMS spectra obtained from the (*S*,*S*)–SINpEt functionalized Au-Ni surfaces, respectively. The m/z obtained in the spectra confirmed the functionalization of the Au-Ni surface with (*S*,*S*)–SINpEt molecules. (c-d) The density mapping images of the respective species [(*S*,*S*)–

SINpEt(H)]$^+$ and [(*S*,*S*)–SINpEt(H)+Au]$^+$ found in the ToF-SIMS spectra revealed the uniform distribution of the (*S*,*S*)–SINpEt molecules functionalized over the Au-Ni surface.

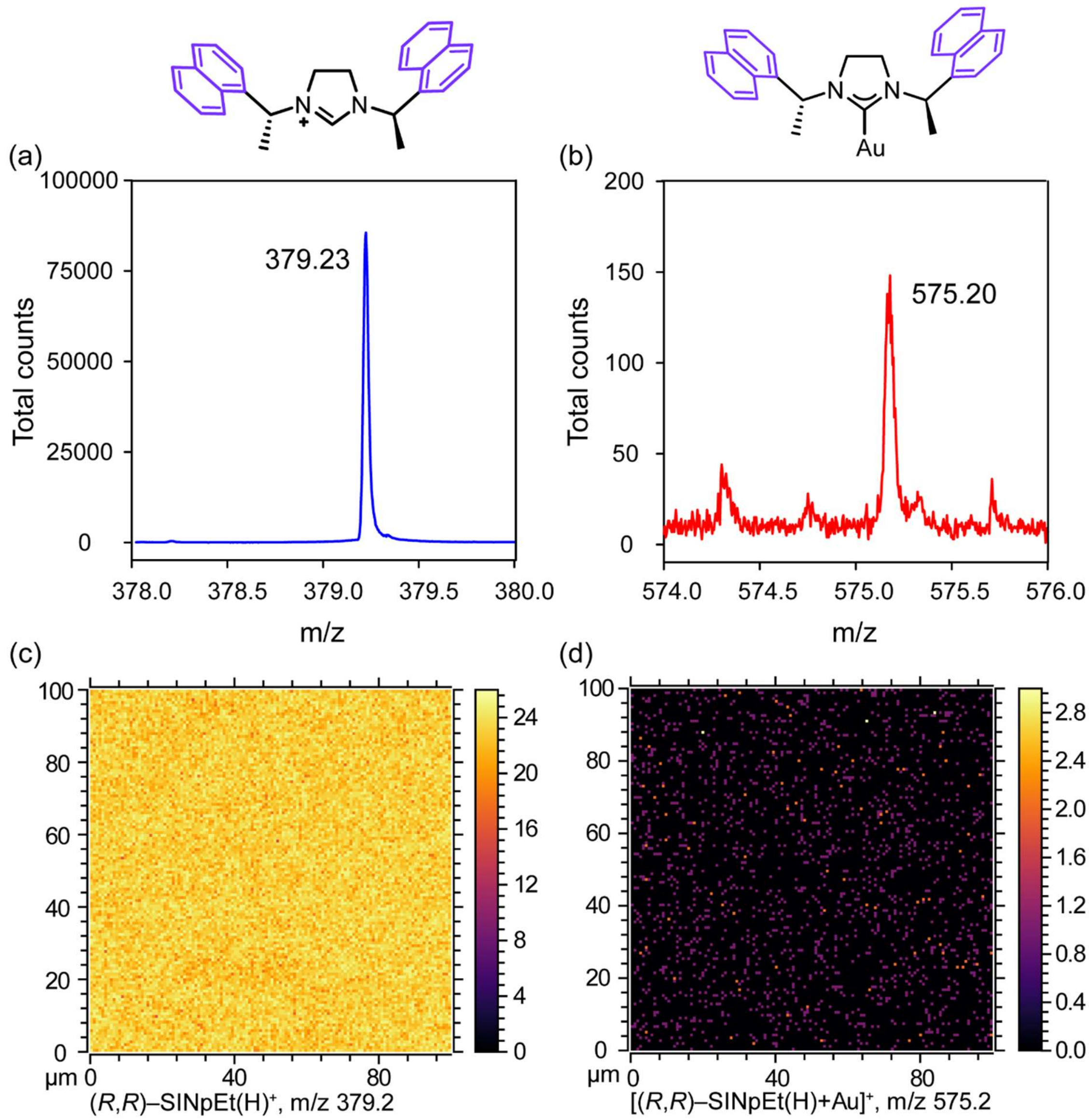

Figure S27. (a-b) ToF-SIMS spectra obtained from the (*R*,*R*)–SINpEt functionalized Au-Ni surfaces, respectively. The m/z obtained in the spectra confirmed the functionalization of the Au-Ni surface with (*R*,*R*)–SINpEt molecules. (c-d) The density mapping images of the respective species [(*R*,*R*)–SINpEt(H)]$^+$ and [(*R*,*R*)–SINpEt(H)+Au]$^+$ found in the ToF-SIMS spectra revealed the uniform distribution of the (*R*,*R*)–SINpEt molecules functionalized over the Au-Ni surface.

We also observed the [(SINpEt)$_2$Au]$^+$ species in the ToF-SIMS analysis on both the (*S*,*S*)–SINpEt@Au and (*R*,*R*)–SINpEt@Au surfaces, which is very characteristic for (benz)imidazolium carbene (such as BIMe). However, the intensity of the corresponding peak

at 953.4 m/z ($C_{54}H_{52}AuN_4^+$) is relatively small in intensity compared to the 379.23 m/z ($C_{27}H_{27}N_2^+$) and the peak at 575.20 m/z ($C_{27}H_{27}AuN_2^+$). The lower stability of the bidentate NHC (SINpEt) complexes with Au compared to the bis complex of the (benz)imidazolium [such as (BIMe)$_2$Au] could be attributed to the relatively lower sputter yield and subsequent lower peak intensity in the ToF-SIMS spectra.

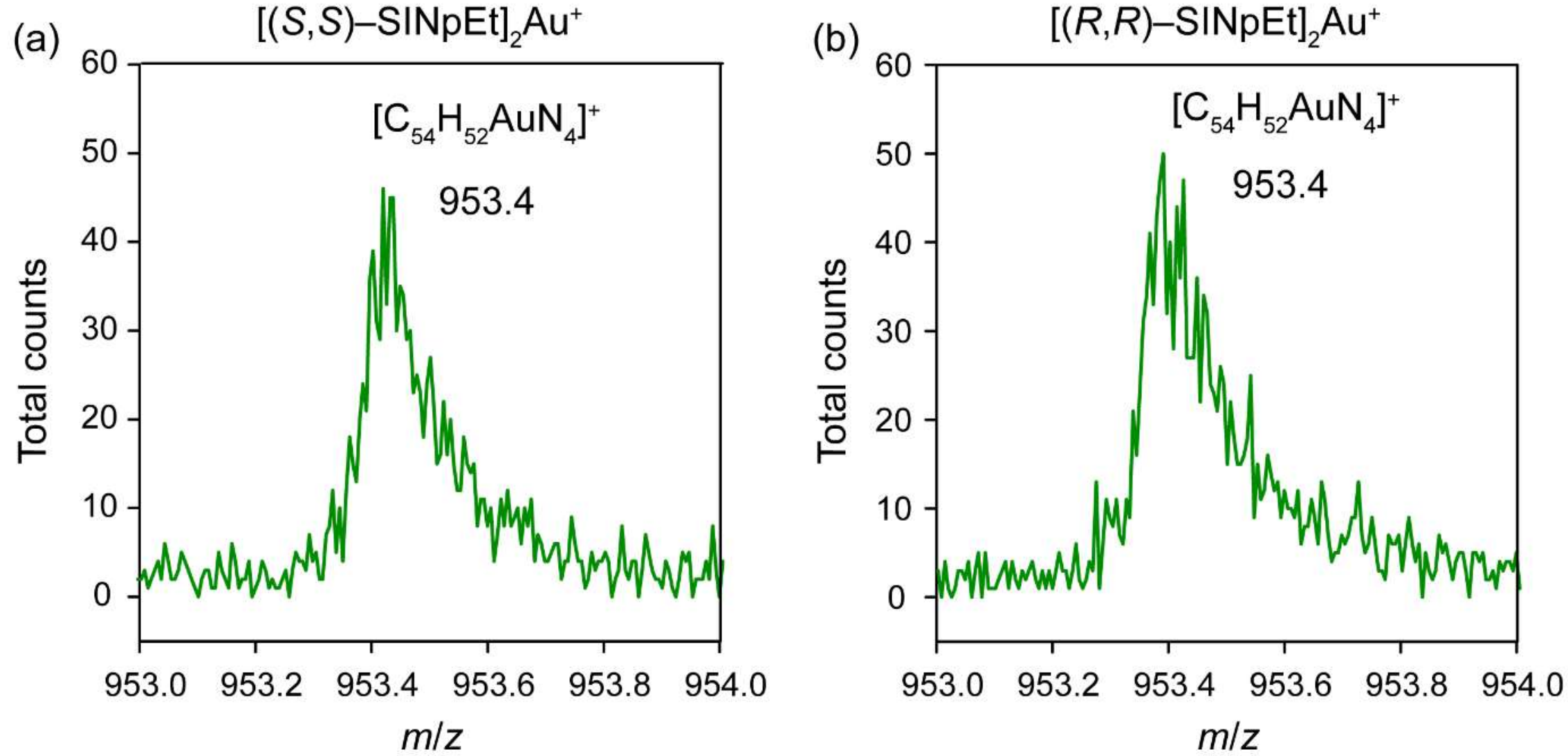

Figure S28. (ToF-SIMS analysis of (a) (*S,S*)–SINpEt@Au and (b) (*R,R*)–SINpEt@Au surfaces, revealing the presence of bidentate SINpEt–Au complexes at 953.4 m/z ($C_{54}H_{52}AuN_4^+$).

In the case of the (*S,S*)–SINpEt@Au-Ni surfaces, fabricated in method 1 (in solution functionalization from NHC(H)·[HCO_3] salt), ToF-SIMS mapping with a larger area, 500 µm x 500 µm, revealed the non-uniform distribution of the SINpEt molecules, and the presence of grain boundaries on the Au-Ni surface. The non-uniformity was also observed with a smaller scan area, 100 µm x 100 µm. We found a similar observation while characterizing the (*S,S*)–SINpEt@Au-Ni surfaces, fabricated in method 2 (free carbene functionalization from NHC(H)·[BF_4] salt). The ToF-SIMS mapping with a 100 µm x 100 µm scan area also revealed the presence of grain boundaries in the (*S,S*)–SINpEt@Au-Ni surfaces.

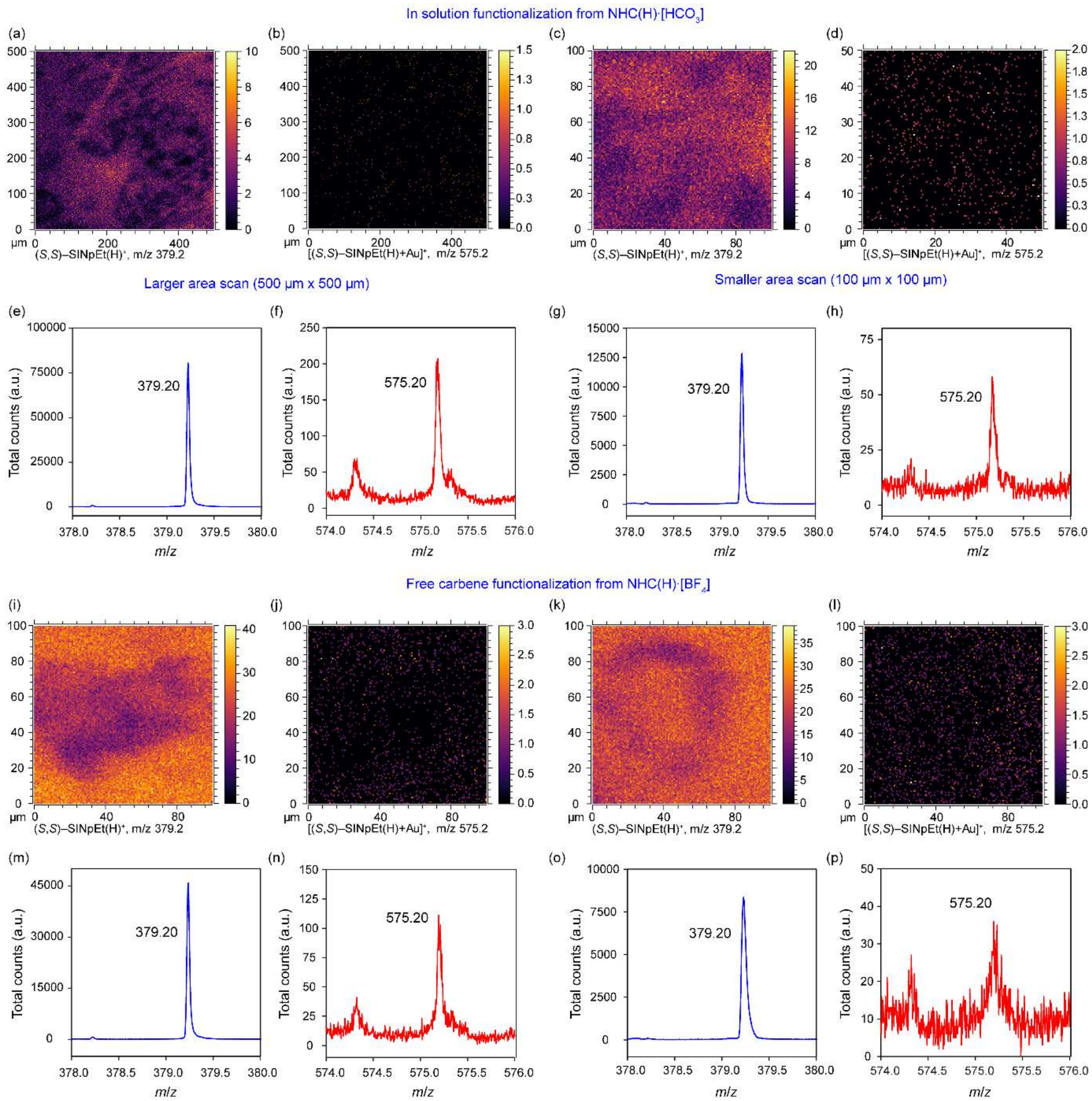

Figure S29. (a-b) Larger area (500 µm x 500 µm) and (c-d) relatively smaller area (100 µm x 100 µm) ToF SIMS mapping of (*S,S*)–SINpEt@Au-Ni surfaces, fabricated *via* the method: in solution in solution functionalization from NHC(H)·[HCO_3] salt. (e-h) Both peak for the $[M+H]^+$, mass 379.23 m/z ($C_{27}H_{27}N_2^+$) and the peak for $[M+Au]^+$ at 575.20 m/z ($C_{27}H_{27}AuN_2^+$) were observed. However, in both cases, peak intensities (total counts) were not consistent throughout the entire surface, which also indicated the non-uniform distribution of SINpEt molecules on the Au-Ni surface. (i-l) A similar phenomenon was also observed for the (*S,S*)–SINpEt@Au-Ni surfaces, fabricated *via* the method: free carbene functionalization from NHC(H)·[BF_4] salt. (m-p) The intensity of the peak for the $[M+H]^+$, mass 379.23 m/z ($C_{27}H_{27}N_2^+$) and the peak for $[M+Au]^+$ at 575.20 m/z ($C_{27}H_{27}AuN_2^+$) were also inconsistent in different areas of the (*S,S*)–SINpEt@Au-Ni surfaces.

Section S-7: Contact angle measurement

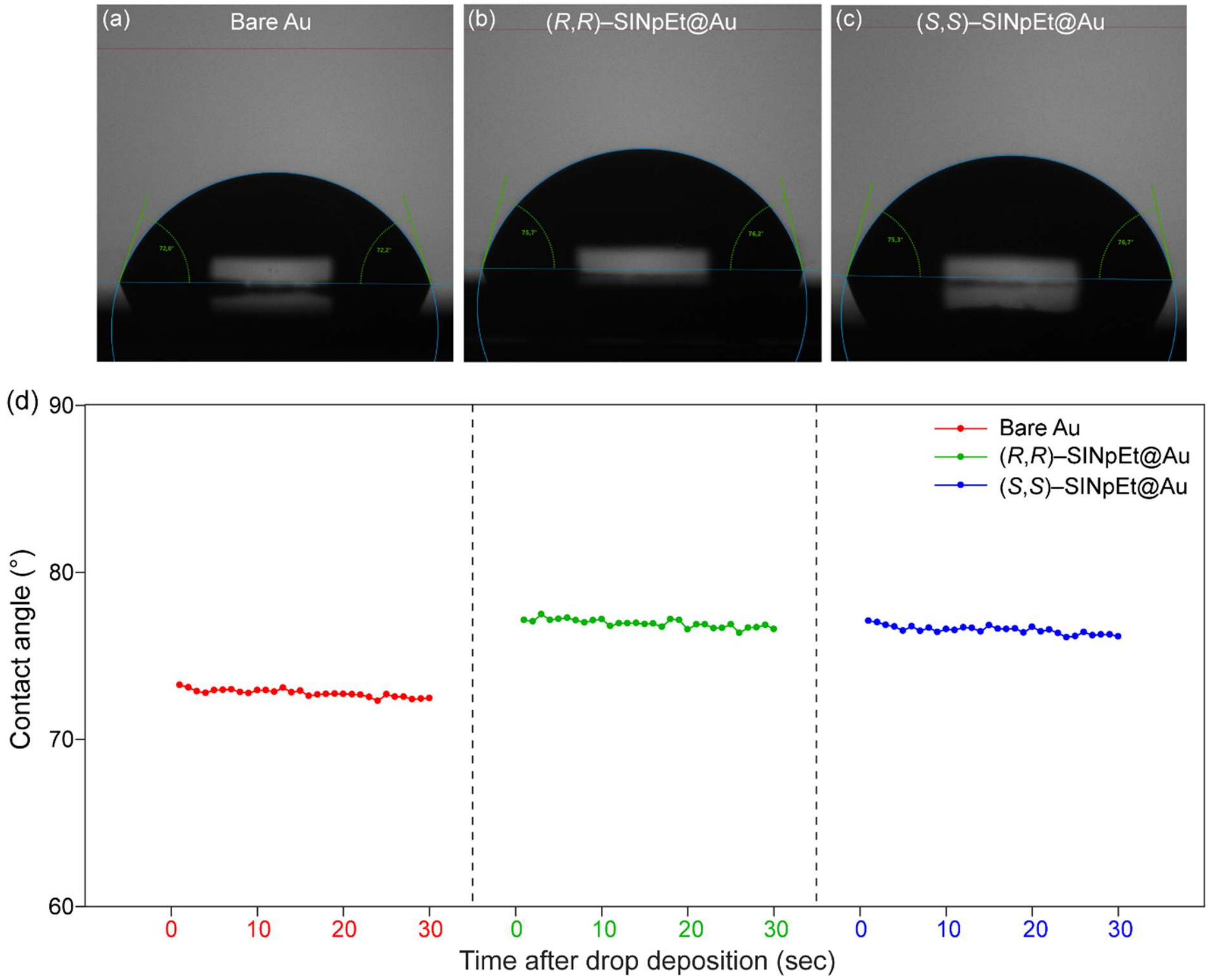

Figure S30. Average contact angle of the water droplet on (a) bare Au surface (surface B), (b) (*R*,*R*)–SINpEt functionalized Au surfaces, and (c) (*S*,*S*)–SINpEt functionalized Au surfaces, measured to be ~72.1°, ~76°, and ~76°, respectively. (d) The stability curve of the water droplet on the bare Au surface (surface B), (*R*,*R*)–SINpEt functionalized Au surfaces, and (*S*,*S*)–SINpEt functionalized Au surfaces with time.

Section S-8: Electrochemical stability

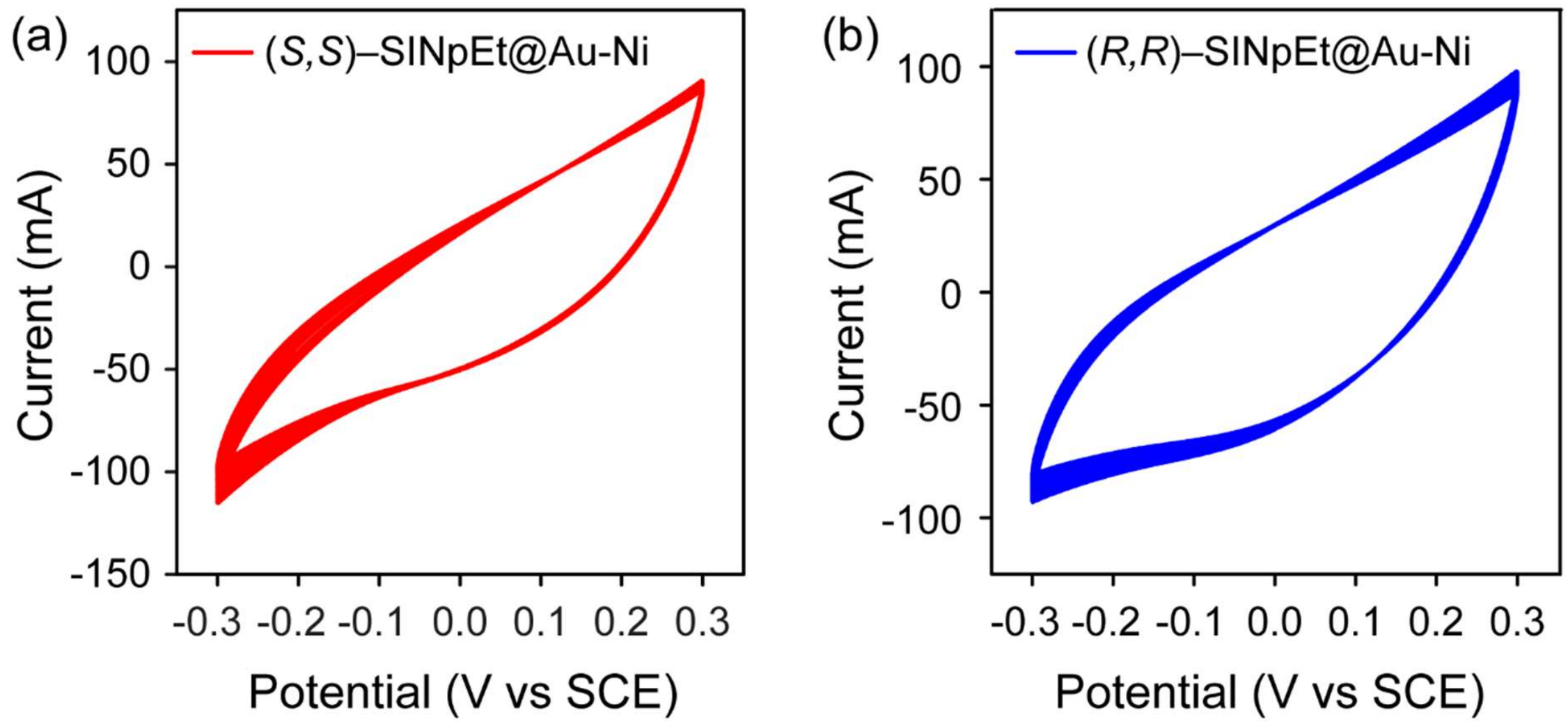

Figure S31. Continuous voltametric interrogation for 50 cycles within -0.3 to 0.3 V vs Ag/AgCl voltage window in an aqueous solution (pH 13) of the (a) (*S,S*)–SINpEt@Au-Ni and (b) (*R,R*)–SINpEt@Au-Ni surfaces, which revealed their electrochemical stability.

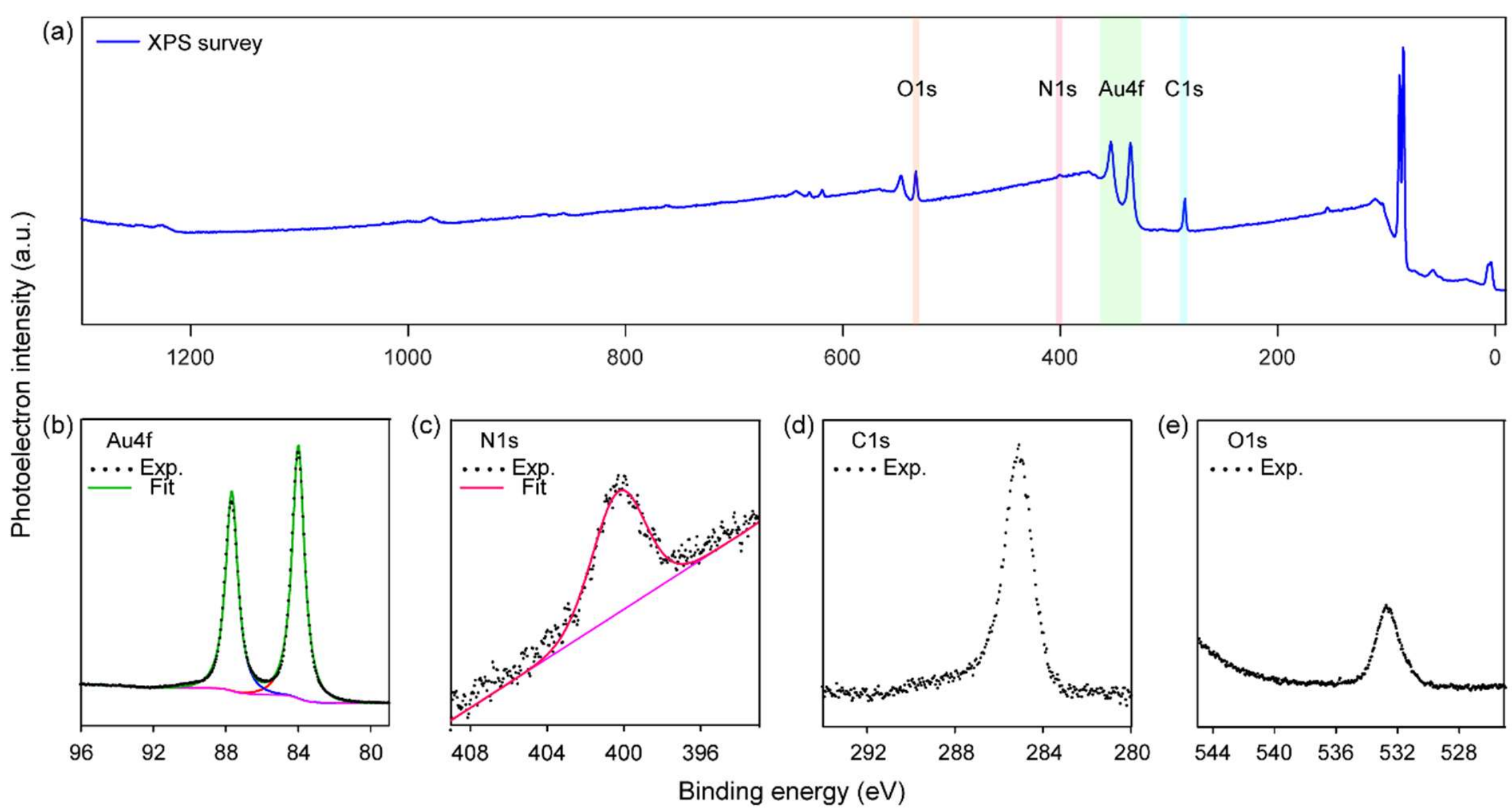

Figure S32: XPS spectra analysis of the (*S,S*)–SINpEt@Au-Ni surface after continuous voltametric interrogation for 50 cycles within -0.3 to 0.3 V vs Ag/AgCl voltage window in an aqueous solution (pH 13). (a) XPS survey spectra. High resolution (b) Au 4f (c) N 1s (d) C 1s and (e) O 1s spectra.

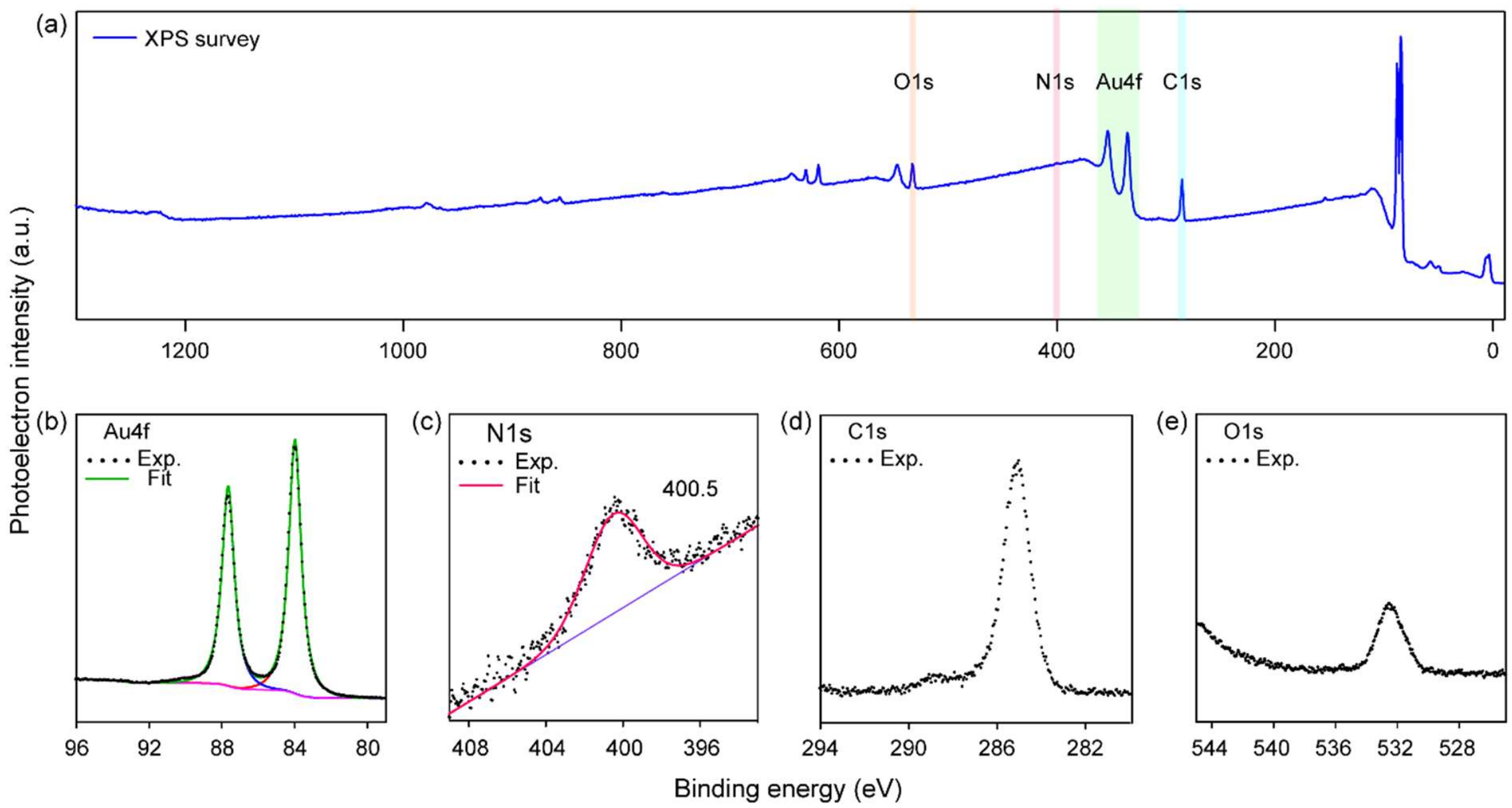

Figure S33: XPS spectra analysis of the (*R*,*R*)–SINpEt@Au-Ni surface after continuous voltametric interrogation for 50 cycles within -0.3 to 0.3 V vs Ag/AgCl voltage window in an aqueous solution (pH 13). (a) XPS survey spectra. High resolution (b) Au 4f (c) N 1s (d) C 1s and (e) O 1s spectra.

Section S-9: Spin-dependent cyclic voltammetry

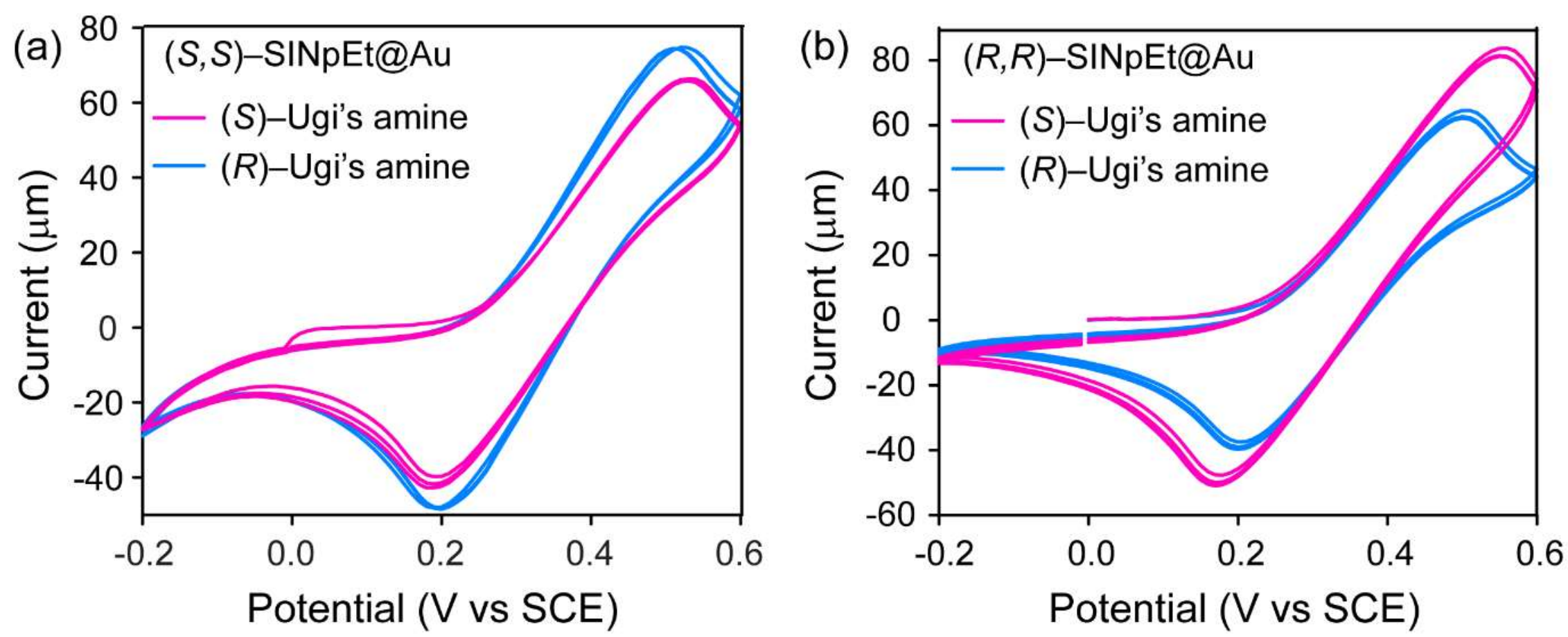

Figure S34. The CV presented in the main manuscript (Figure 5a-b) corresponds to the first CV scan from a single representative experiment. Additional CV cycles of the chiral N,N-dimethyl-1-ferrocenyl-ethylamine [(*R*)–and (*S*)–Ugi's amine], obtained while using the (a) (*S*,*S*)–SINpEt@Au and (b) (*R*,*R*)–SINpEt@Au surfaces as electrodes, respectively.

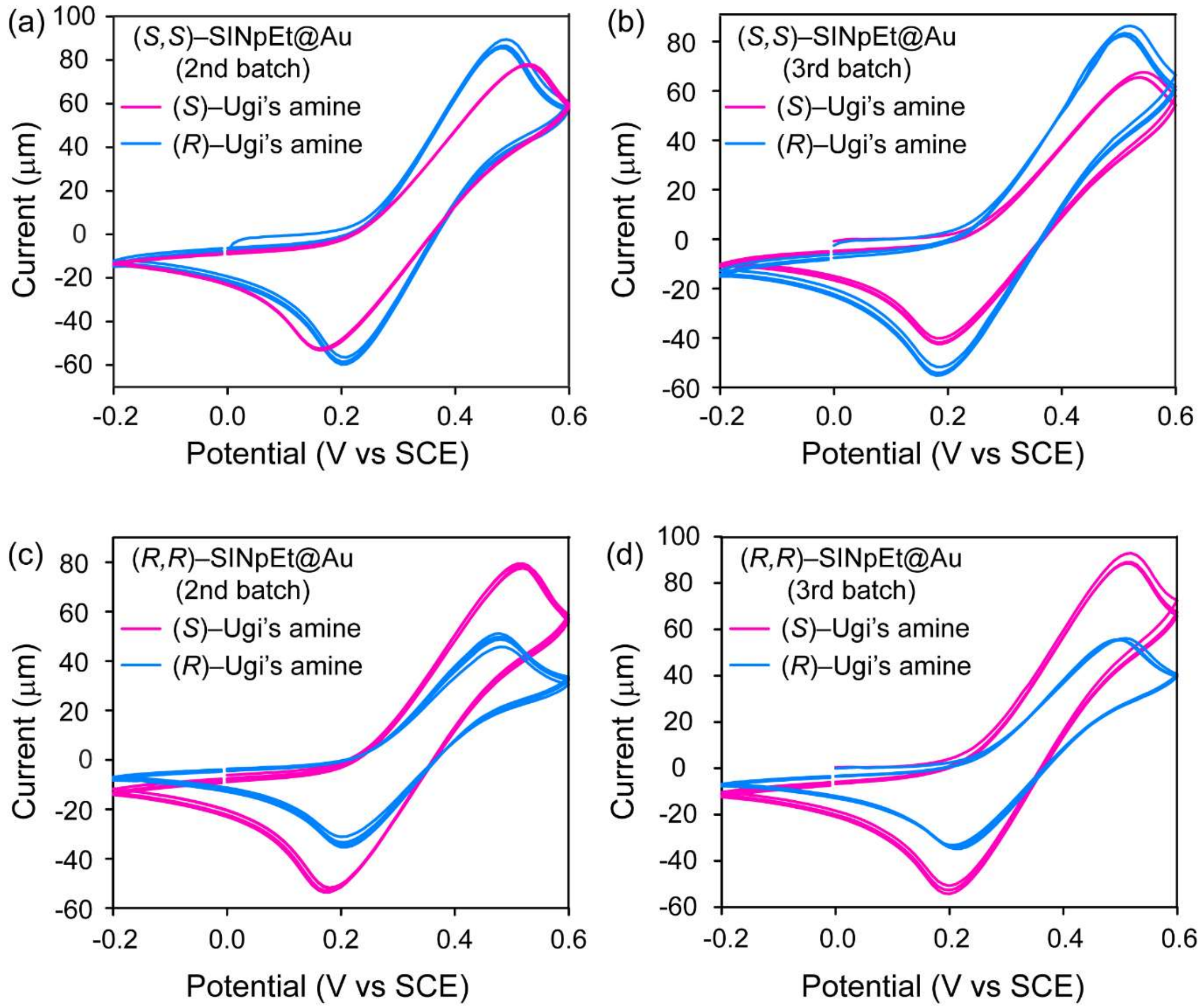

Figure S35. CV cycles of the chiral N,N-dimethyl-1-ferrocenyl-ethylamine [(*R*)–and (*S*)–Ugi's amine] obtained while using the 2nd batch and 3rd batch of (a-b) (*S*,*S*)–SINpEt@Au and (c-d) (*R*,*R*)–SINpEt@Au surfaces as electrodes, respectively, to check their reproducibility.

Section S-10: mcAFM measurements

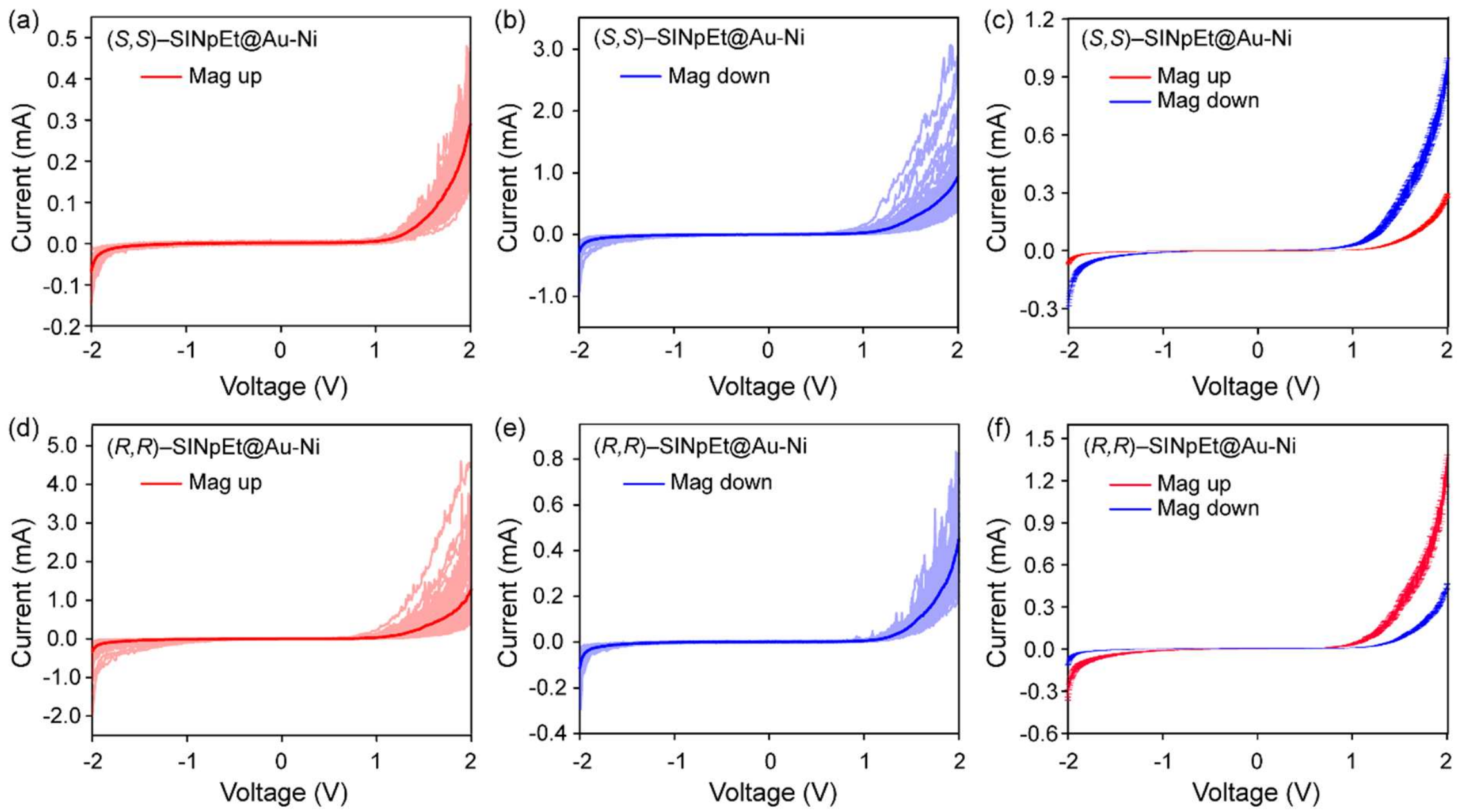

Figure S36: The current versus voltage (IV) curves measured for the (a-c) (*S*,*S*)–SINpEt@Au-Ni and (d-f) (*R*,*R*)–SINpEt@Au-Ni surfaces. The red curves correspond to the magnet's North pole pointing UP, and the blue curves correspond to the magnet's North pole pointing DOWN.

Section S-11: ORR with chiral Au surface

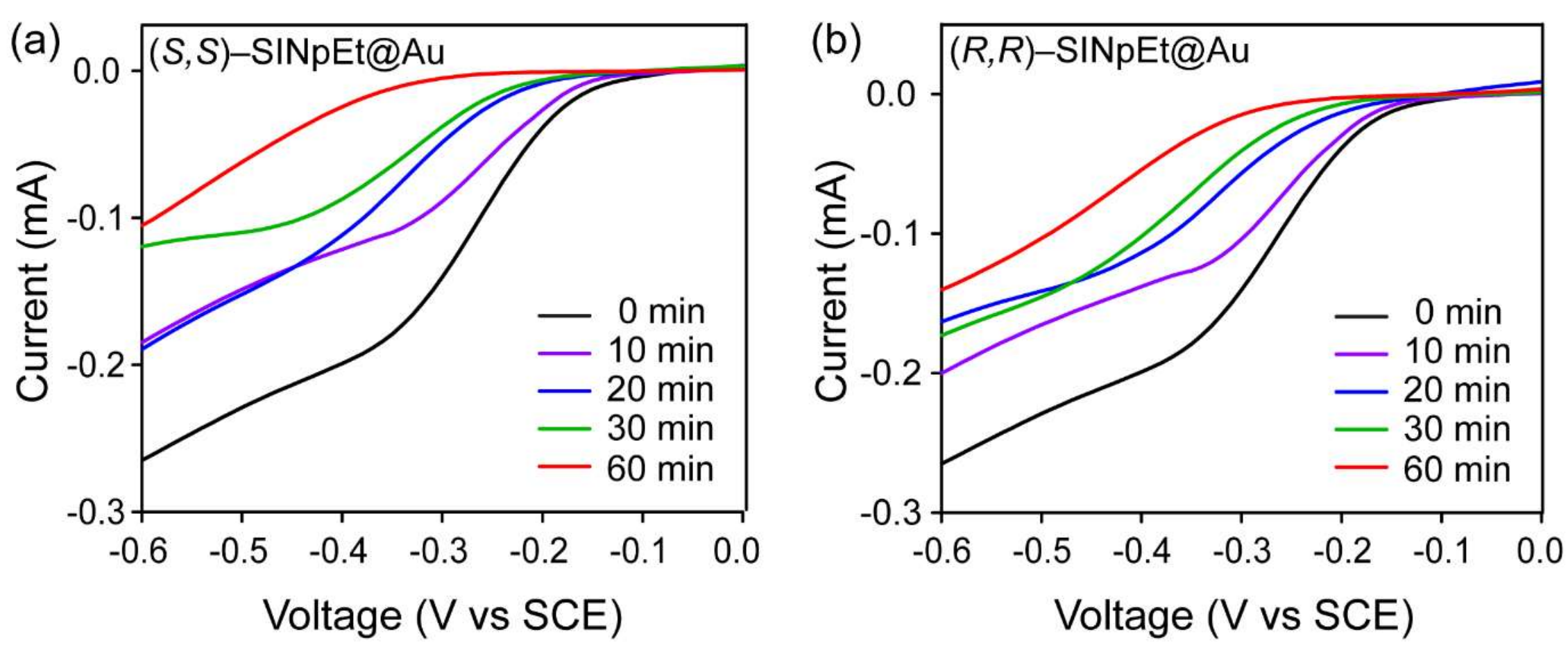

Figure S37: The ORR polarization curves of (c) (*S*,*S*)–SINpEt, (d) (*R*,*R*)–SINpEt functionalized Au surface at different thicknesses, controlled by the different deposition times.

We investigated the ORR performance of all the surfaces with different molecular coverage and thickness [both (*S*,*S*)-SINpEt@Au and (*R*,*R*)-SINpEt@Au]. We observed that the ORR

efficiency is thickness-dependent when we recorded the linear sweep voltammograms (LSV) for different thicknesses of (*S*,*S*)-SINpEt@Au and (*R*,*R*)-SINpEt@Au. The efficiency of ORR can be evaluated from various factors, i.e., onset potential, onset current, peak potential, and peak currents. Figures 5c and 5d revealed that as the thickness of chiral NHCs increased, both the reduction current and onset potential changed correspondingly with thickness. Hence, all those parameters are correlated. Figures 5e and 5f show the onset current and potential as a function of the film thickness, revealing a decrease in current with increasing thickness, accompanied by a weak oscillatory feature.

Deposition time (minutes)	Reduction current (mA) [(*R*,*R*)–SINpEt]	Onset Potential (V) [(*R*,*R*)–SINpEt]	Reduction current (mA) [(S,S)–SINpEt]	Onset Potential (V) [(S,S)–SINpEt]
0	0.097±0.006	-0.180±0.001	0.097±0.006	-0.180±0.001
10	0.073±0.001	-0.191±0.002	0.063± 0.001	-0.198±0.002
20	0.034±0.005	-0.247±0.011	0.027± 0.002	-0.312±0.004
30	0.022±0.005	-0.294±0.017	0.020±0.003	-0.286±0.014
60	0.0071±0.002	-0.344±0.012	0.002±.01165E-4	-0.402±0.003

Section S-12: ORR control experiment with achiral Au surface

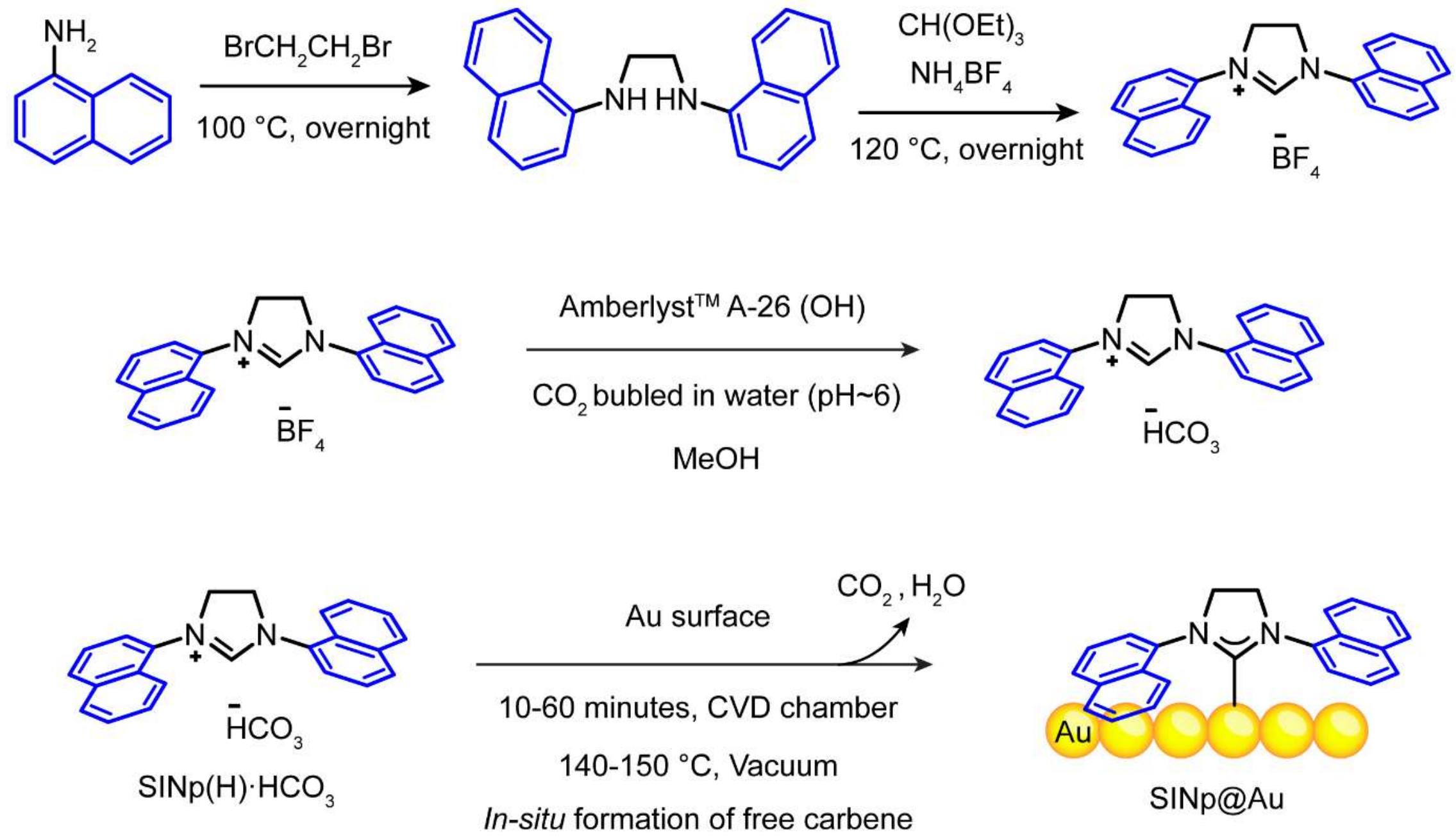

Figure S38: Synthesis of an achiral NHC precursor named SINp(H)·[HCO_3], (structurally similar to SINpEt), and the functionalization of the Au surface with the respective SINp molecules at different deposition times (10 min, 20 min, 30 min to 60 min) *via* chemical vapour deposition.

As a control experiment to confirm the effect of chirality on ORR efficiency, we also fabricated achiral NHC-functionalized Au surfaces following the aforementioned chemical vapour deposition procedure. We synthesised an achiral NHC precursor, named SINp(H)·[HCO_3], (structurally similar to SINpEt), to functionalize the Au surface *via* chemical vapour deposition at different time intervals (10 min, 20 min, 30 min, and 60 min) with SINp molecules. All the surfaces were thoroughly washed with isopropanol followed by dry acetone. The ^{1}H, ^{13}C NMR and ESI-MS confirmed the synthesis of SINp(H)·[HCO_3]. The direct inlet EIMS indicated an evaporation temperature of ~130 °C.

^{1}H NMR (400 MHz, $CDCl_3$): 8.36 (dq, *J* =8.5 Hz, 2H), 8.17-8.10 (m, 4H), 7.96 (dd, *J* =7.4 Hz, 1.1 Hz, 2H), 7.87-7.82 (m, 2H), 7.75-7.68 (m, 4H), 4.91 (s, 4H). The C2 and HCO_3 protons could not be observed due to their rapid exchange with the deuterated solvent on the NMR time scale.

^{13}C NMR (75 MHz, $CDCl_3$): 165.9, 134.6, 132.2, 130.5, 128.8, 128.1, 127.1, 125.3, 124.3, 121.3, 53.5. The carbene C2 is expected to be broadened by solvent exchange on the time scale of the NMR measurement.

ESI-MS: calculated for $[C_{23}H_{19}N_2]^+$: 323.1543, found: 379.15450.

Direct inlet EIMS: m/z (% rel. abundance) calculated for $[M]^{\cdot+} = [C_{23}H_{18}N_2]^{\cdot+}$ 322.2, at 130 (±20) °C, found $[M+H_2O]^{\cdot+}$ 340.2)

We performed XPS analysis on four Au surfaces functionalized with SINp (SINp@Au) at different deposition times: 10 min, 20 min, 30 min, and 60 min. The N 1s peak at ~400.5 (±0.2) in the XP spectra confirmed the functionalization of the Au surface with SINp molecules (Figure S35). We calculated the molecular coverage at different deposition times and found that the molecular coverage gradually increased from 0.55 to 0. 85, to 1.45 to 1.55 molecules nm^{-2} (Figure S35). We also performed the ORR on the fabricated SINp@Au surfaces, using them as electrodes. In the case of achiral NHC (SINp) functionalized Au surfaces, the oxygen reduction peak and overpotential shifted to very high voltage. Previously, in the case of (*S*,*S*)-SINpEt and (*R*,*R*)-SINpEt functionalized Au surfaces, their electronic spin filtration reduced the overpotential of ORR, making the reaction more efficient. Whereas, in the case of achiral SINp@Au, no spin filtering/polarization was observed, which was also reflected through their efficiency in ORR.

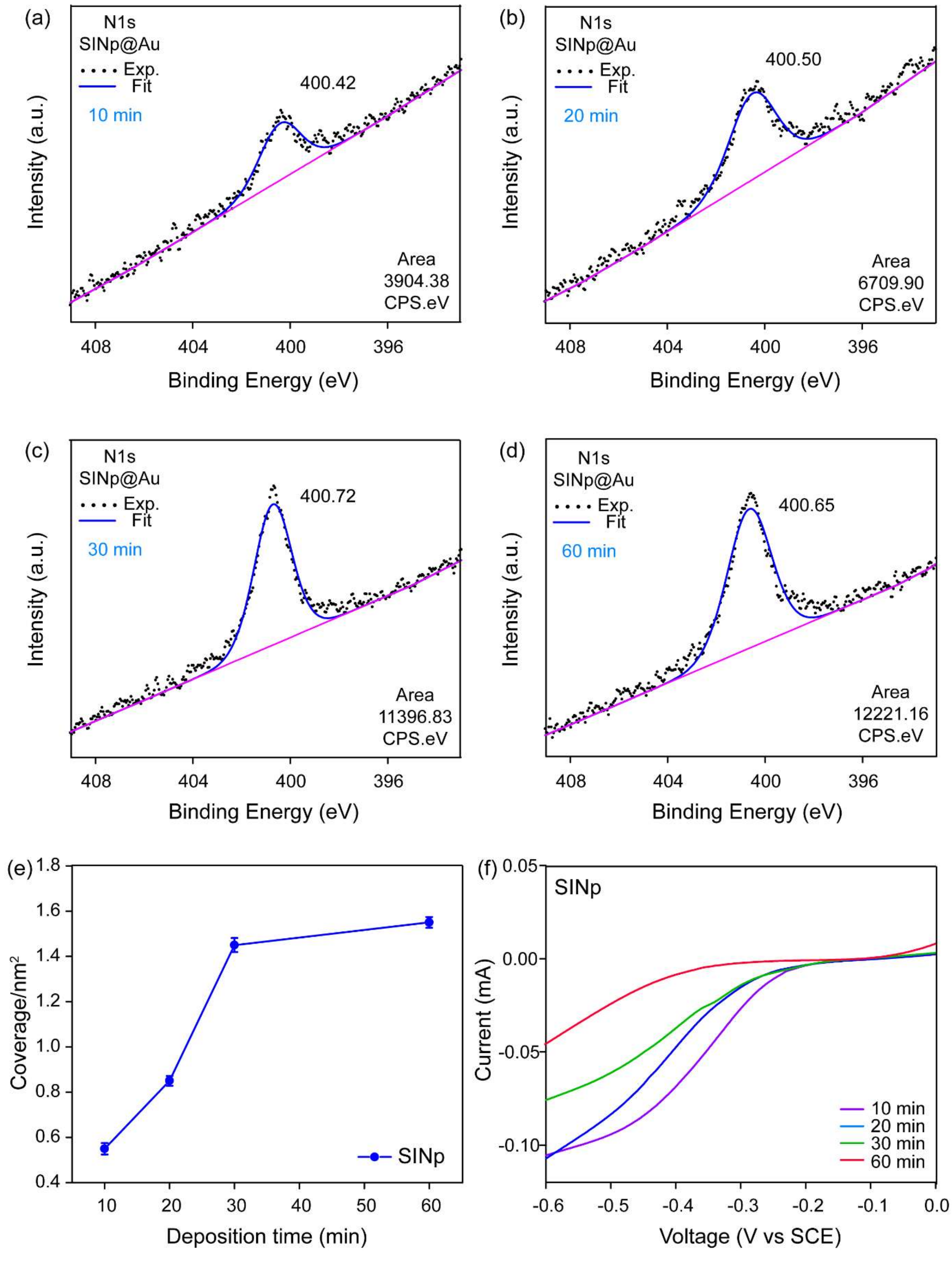

Figure S39: (a-d) N 1s XP spectra of the SINp@Au surfaces, obtained at different deposition times (10 min, 20 min, 30 min to 60 min). (e) The molecular surface coverage of SINp molecules on the Au surface, obtained at different deposition times during the chemical vapour deposition. (f) Linear sweep voltammograms (LSV) of the fabricated SINp@Au surfaces [obtained at different deposition times (10 min, 20 min, 30 min to 60 min *via* chemical vapour deposition], obtained in a three-electrode cell setup

for ORR. The reference electrode was a Hg/Hg_2Cl_2/saturated KCl (saturated calomel electrode), and the counter electrode was a platinum wire. A 0.1 M KOH solution served as the electrolyte at a pH of 12.6.

Table R5: The relative signal intensities of Au 4f7/2, N 1s (normalized to add up to 100 %).

	SINp@Au (10 minutes)	SINp@Au (20 minutes)	SINp@Au (30 minutes)	SINp@Au (60 minutes)
I_{Au4f7}^{rel}	97.72 %	95.85 %	92.04 %	91.46 %
I_{N1s}^{rel}	2.28 %	4.15 %	7.96 %	8.54 %

Table R6: The relative signal intensities of I_{Au4f7} and I_{Au4f7}^{0} for different surfaces.

	SINp@Au (10 minutes)	SINp@Au (20 minutes)	SINp@Au (30 minutes)	SINp@Au (60 minutes)
$\frac{I_{Au4f7}}{I_{Au4f7}^{0}}$	0.49	0.40	0.35	0.34

Using the following equation, we calculated the molecular surface coverage.

$$\frac{n_{NHC}}{A} = \frac{1}{2}\frac{I_{N1s}^{rel}}{I_{Au4f7}^{rel}}\,\lambda_{Au4f7}^{Au}\,\frac{n_{Au}}{V} \times \frac{I_{Au4f7}}{I_{Au4f7}^{0}}$$

Deposition time (minutes)	Coverage (molecules/nm^2) of SINp on the Au surface
10	0.55±0.025
20	0.85±0.022
30	1.45±0.031
60	1.55±0.024

Section S-13: Theoretical calculations

Computational details

Calculations have been performed using QuantumEspresso version 7.3.1[10,11] on the University of Münster's HPC cluster Palma II. The vdW-DF-obk8 density functional12 was used for calculations, as implemented in QuantumEspresso, to include explicit van der Waals corrections and reduce potential overbinding of the flat geometry due to Grimme-type dispersion corrections[13-17]. Pseudopotentials have been sourced from the SSSP PBE efficiency v1.1.2 library[18]. In particular, H.pbe-kjpaw_psl.1.0.0.UPF, N.pbe-kjpaw_psl.1.0.0.UPF, C.pbe-n-kjpaw_psl.1.0.0.UPF, and Au.pbe-n-kjpaw_psl.1.0.0.UPF have been used for the corresponding elements. The kinetic-energy cutoff (ecutwfc) was set to 60 Ry while the cutoff for the electron density (ecutrho) was set to 550 Ry. Geometry optimizations were performed using the BFGS algorithm[19]. The Marzari-Vanderbilt-DeVita-Payne cold smearing[20] method was used, incorporating a metal (gold) slab with a degauss value of 0.0038 Ry, to evaluate adsorption energies. Γ-point sampling was used here due to the large size of the unit cells considered in this study. For all calculations, the bottom layer of the metal slab was frozen during optimization to simulate the bulk material. In contrast, the top 2 layers could adjust based on interactions with the molecules.

For the charge density difference analysis, calculations have been performed in CP2K[21,22] using the Gaussian Plane Wave method. A double-zeta Gaussian basis set was used for the atoms H, C, N (DZVP-MOLOPT-GTH) and for Au (DZVP-MOLOPT-GTH-SR)[23], alongside Goedecker-Teter-Hutter[24,25,26] pseudopotentials. A planewave cutoff of 550 Ry and a relative cutoff of 60 Ry have been employed. The PBE[27] density functional has been used with Grimme's D3 dispersion correction with Becke-Johnson damping[28,29]. Calculations have been performed based on the geometries obtained from QuantumEspresso.

Coverage based on surface unit cell size

Based on the unit cell parameters for each AxBx3 unit cell, generated using the ASE[30] library for Python, considered in Table S5 below, the surface area can be calculated. From this, one can directly calculate the coverage, as only unit cells containing one molecule per unit cell were considered in the calculations. The coverages obtained in this manner are reported here for a few example surface unit cell sizes.

Table S7: Coverage of SINpEt@Au in molecules per nm² based on simulated surface unit cell size given as multiple of surface unit vectors.

Size of surface unit cell	Coverage [molecules/nm²]
4x3	1.156
5x3	0.925
6x3	0.771
7x3	0.661
5x4	0.694
6x4	0.578
7x4	0.496

Charge transfer analysis

The charge density difference was calculated in each case by taking the .cube files for the full system, as well as the individual molecule and surface obtained by single point calculations in CP2K. The geometries used are identical to the optimized structures for each case as obtained with QuantumEspresso. In this manner, only the electronic interaction is probed. Integrating over the (*x,y*) plane results in the charge density difference $\Delta\rho$ as a function of height z. To calculate the total charge transfer q_{CT}, one identifies the coordinate z_{min}, where the zero-crossing from positive to negative occurs between the surface and the molecule, then integrates up to z_{max}, where the charge density difference has decayed to 0.

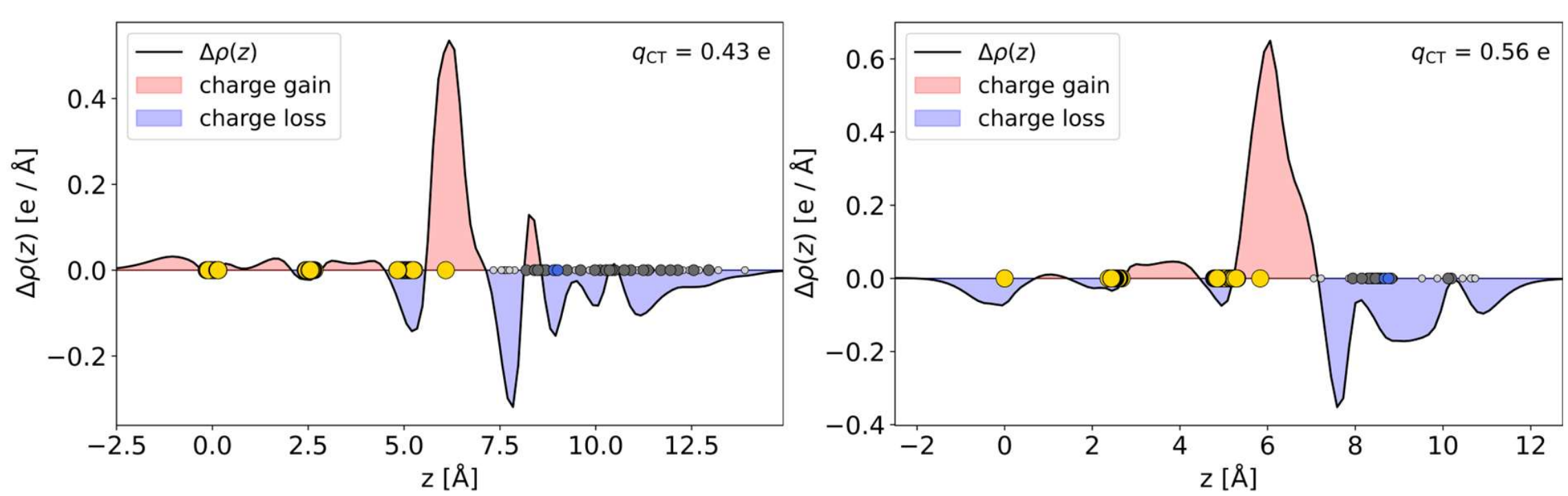

Figure S40: Charge density difference $\Delta\rho$ as a function of height z for the 11×9 (low coverage) surface together with the charge transfer q_{CT}. The atomic positions are indicated by coloured circles. Au =

yellow, H = light gray, C = dark gray, N = blue. The left image shows results for the upright geometry. The right image shows results for the flat geometry.

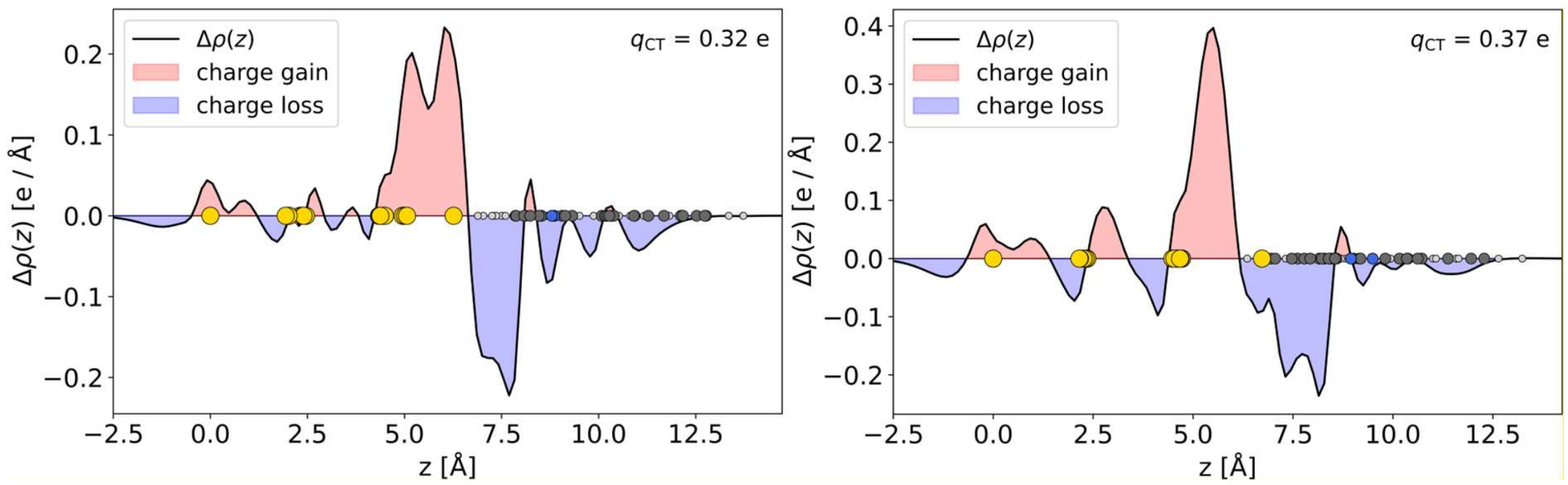

Figure S41: Charge density difference $\Delta\rho$ as a function of height z for the 5×3 (high coverage) surface together with the charge transfer q_{CT}. The position of the atoms is indicated by coloured circles. Au = yellow, H = light gray, C = dark gray, N = blue. The left image shows results for the upright geometry. The right image shows results for the flat geometry.

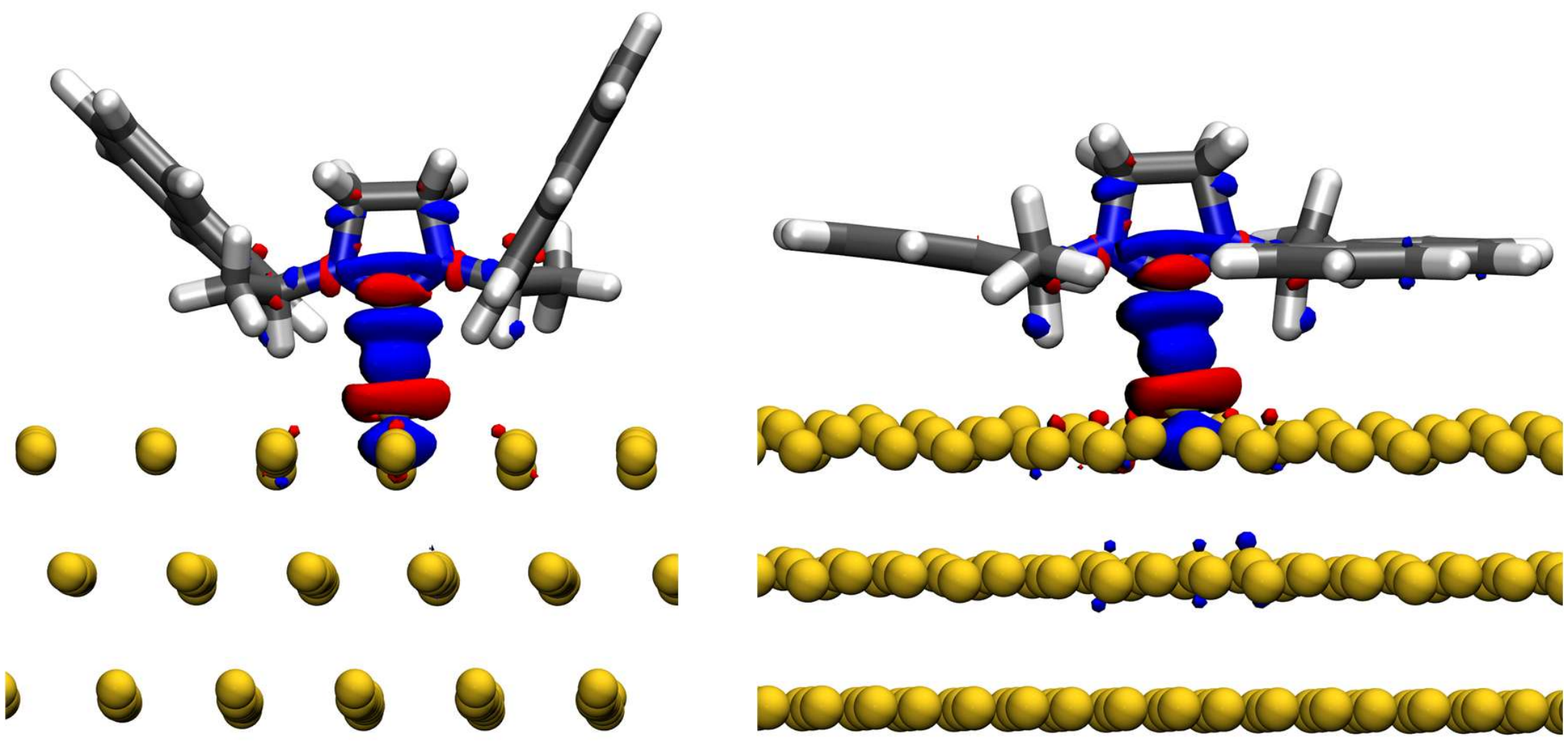

Figure S42: Charge density difference plotted at an isodensity value of 0.003 (positive: red, negative: blue) for the 11×9 surface unit cell. On the left is the upright geometry. On the right is the flat geometry.

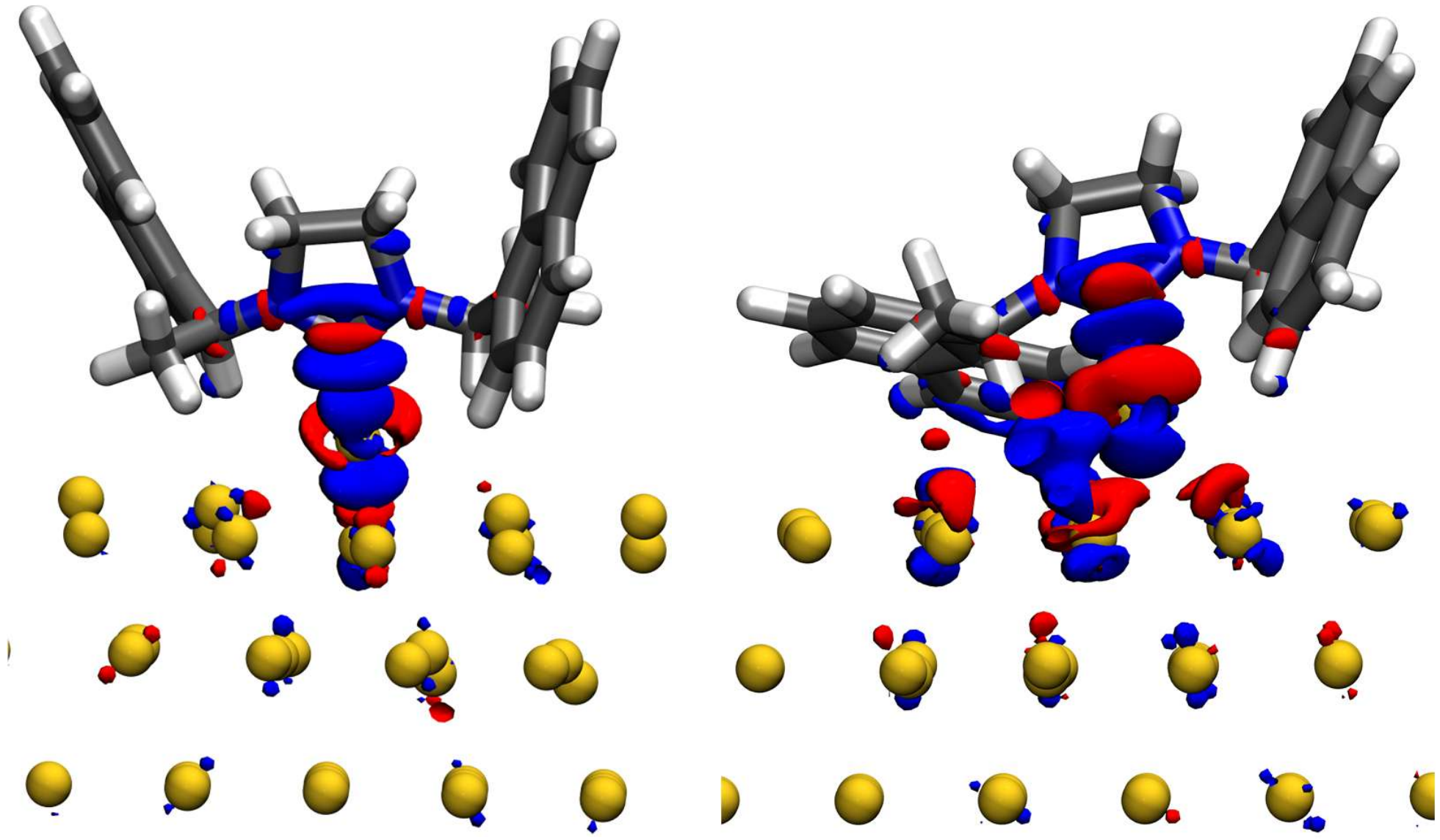

Figure S43: Charge density difference plotted at an isodensity value of 0.003 (positive: red, negative: blue) for the 5×3 surface unit cell. On the left is the upright geometry. On the right is the flat geometry.

Section S-14: References

1. Urban, S., Ortega, N. & Glorius, F. Ligand-Controlled Highly Regioselective and Asymmetric Hydrogenation of Quinoxalines Catalyzed by Ruthenium N-Heterocyclic Carbene Complexes. *Angew. Chem. Int. Ed.* **2011**, 50, 3803 –3806.
2. Crudden, C. M., Horton, J. H., Narouz, M. R., Li, Z., Smith, C. A., Munro, K., Baddeley, C. J., Larrea, C. R., Drevniok, B., Thanabalasingam, B., McLean, A. B., Zenkina, O. V., Ebralidze, I. I.; She, Z., Kraatz, H.-B., Mosey, N. J., Saunders, L. N., & Yagi, A. Simple direct formation of self-assembled N-heterocyclic carbene monolayers on gold and their application in biosensing. *Nat. Commun*. **2016**, 7, 12654.
3. Chandran, A., Dominique, N. L., Kaur, G., Clark, V., Nalaoh, P., Ekowo, L. C., Jensen, I. M., Aloisio, M. D., Crudden, C. M., Arroyo-Currás, N., Jenkins, D. M., & Camden, J. P. Forming N-heterocyclic carbene monolayers: not all deposition methods are the same. *Nanoscale* **2025**, 17, 5413–5428.
4. Franz, M., Chandola, S., Koy, M., Zielinski, R., Aldahhak, H., Das, M., Freitag, M., Gerstmann, U., Liebig, D., Hoffmann, A. K., Rosin, M., Schmidt, W. G., Hogan, C., Glorius, F., Esser, N., & Dähne, M. Controlled growth of ordered monolayers of N-heterocyclic carbenes on silicon. *Nat. Chem*. **2021**, 13, 828–835.

5. Gutheil, C., Gina Roß, G., Amirjalayer, S., Mo, B., Schäfer, A. H., Doltsinis, N. L., Braunschweig, B., & Glorius, F. Tailored Monolayers of N-Heterocyclic Carbenes by Kinetic Control. *ACS Nano* **2024**, 18, 3043−3052.
6. Seah, M. P. & Dench, W. A. Quantitative Electron Spectroscopy of Surfaces: A Standard Data Base for Electron Inelastic Mean Free Paths in Solids. *Surface and Interface Analysis* **1979**, 1, 2–11.
7. Jablonski, A. & Powell, C. J. The Electron Attenuation Length Revisited. *Surf Sci Rep* **2002**, 47, 33–91.
8. Kim, H. K., Hyla, A. S., Winget, P., Li, H., Wyss, C. M., Jordan, A. J., Larrain, F. A., Sadighi, J. P., Fuentes-Hernandez, C., Kippelen, B., Brédas, J. L., Barlow, S. & Marder, S. R. Reduction of the Work Function of Gold by N-Heterocyclic Carbenes. *Chem. Mater.* **2017**, 29, 3403–3411.
9. Qi, L., Mayall, R. M., Lee, D. S., Smith, C., Woods, A., Narouz, M. R., Hyla, A., Bhattacharjee, H., She, Z., Crudden, C. M. & Birss, V. I. Energetics and Redox Kinetics of Pure Ferrocene-Terminated N-Heterocyclic Carbene Self-Assembled Monolayers on Gold. *Langmuir* **2024**, 40, 17367–17377.
10. Gianozzi, P., Baroni, S., Bonini, N., Calandra, M., Car, R., Cavazzoni, C., Ceresoli, D., Chiarotti, G. L., Cococcioni, M., Dabo, I., Dal Corso, A., de Gironcoli, S., Fabris, S., Fratesi, G., Gebauer, R., Gerstmann, U., Gougoussis, C., Kokalj, A., Lazzeri, M., Martin-Samos, L., Marzari, N., Mauri, F., Mazzarello, R., Paolini, S., Pasquarello, A., Paulatto, L., Sbraccia, C., Scandolo, S., Sclauzero, G., Seitsonen, A. P., Smogunov, A., Umari, P. & Wentzcovitch, R. M. QUANTUM ESPRESSO: a modular and open-source software project for quantum simulations of materials. *J. Phys.: Condens. Matter* **2009,** 21, 395502.
11. Giannozzi, P., Andreussi, O., Brumme, T., Bunau, O., Buongiorno Nardelli, M., Calandra, M., Car, R., Cavazzoni, C., Ceresoli, D., Cococcioni, M., Colonna, N., Carnimeo, I., Dal Corso, A., de Gironcoli, S., Delugas, P., DiStasio, R. A. Jr, Ferretti, A., Floris, A., Fratesi, G., Fugallo, G., Gebauer, R., Gerstmann, U., Giustino, F., Gorni, T., Jia, J., Kawamura, M., Ko, H.-Y., Kokalj, A., Küçükbenli, E., Lazzeri, M., Marsili, M., Marzari, N., Mauri, F., Nguyen, N. L., Nguyen, H.-V., Otero-de-la-Roza, A., Paulatto, L., Poncé, S., Rocca, D., Sabatini, R., Santra, B., Schlipf, M., Seitsonen, A. P., Smogunov, A., Timrov, I., Thonhauser, T., Umari, P., Vast, N., Wu, X. & Baroni, S. Advanced capabilities for materials modelling with QUANTUM ESPRESSO. *J. Phys.: Condens. Matter* **2017**, 29, 465901.

12. Klimeš, J., Bowler, D. R., Michaelides, A., Chemical accuracy for the van der Waals density functional. *J. Phys.: Condens. Matter* **2010**, 22, 022201.
13. Grimme, S. Accurate description of van der Waals complexes by density functional theory including empirical corrections. *J. Comput. Chem*. **2004**, 25, 1463-1473.
14. Grimme, S., Antony, J., Ehrlich, S. & Krieg, H. A consistent and accurate ab initio parametrization of density functional dispersion correction (DFT-D) for the 94 elements H-Pu. *J. Chem. Phys*. **2010**, 132, 154104.
15. Dion, M., Rydberg, H., Schröder, E., Langreth, D. C. & Lundqvist, B. I. Van der Waals Density Functional for General Geometries. *Phys. Rev. Lett*. **2004**, 92, 246401.
16. Li, G., Tamblyn, I., Cooper, V. R., Gao, H.-J. & Neaton, J. B. Molecular adsorption on metal surfaces with van der Waals density functionals. *Phys. Rev. B*. **2012**, 85 121409.
17. Maurer, R. J., Ruiz, V. G., Camarillo-Cisneros, J., Liu, W., Ferri, N., Reuter, K. & Tkatchenko, A. Adsorption structures and energetics of molecules on metal surfaces: Bridging experiment and theory. *Prog. Surf. Sci*. **2016**, 91, 72-100.
18. Prandini, G., Marrazzo, A., Castelli, I. E., Mounet, N. & Marzari, N. Precision and efficiency in solid-state pseudopotential calculations. *Npj Comput. Mater*. **2018**, 4, 72.
19. Broyden, C. G. The convergence of a Class of Double-rank Minimization Algorithms 1. General Considerations, IMA *J. Appl. Math*. **1970**, 6, 76-90.
20. Marzari, N., Vanderbilt, D., De Vita, A. & Payne, M. C. Thermal Contraction and Disordering of the Al(110) Surface. *Phys. Rev. Lett*. **1999**, 82, 3296.
21. CP2K developers group. (2018), CP2K is freely available from https://www.cp2k.org/.
22. Kühne, T. D. et al. CP2K: An electronic structure and molecular dynamics software package - Quickstep: Efficient and accurate electronic structure calculations. *J. Chem. Phys.* **2020**, 152, 194103.
23. VandeVondele, J. & Hutter, J. Gaussian basis sets for accurate calculations on molecular systems in gas and condensed phases. *J. Chem. Phys.* **2007**, 127, 114105.
24. Hartwigsen, C., Goedecker, S. & Hutter, J. Separable dual-space Gaussian pseudopotentials. *Phys. Rev. B.* **1996**, 54, 1703.
25. Hartwigsen, C., Goedecker, S. & Hutter, J. Relativistic separable dual-space Gaussian pseudopotentials from H to Rn. *Phys. Rev. B.* **1998**, 58, 3641.
26. Krack, M. Pseudopotentials for H to Kr optimized for gradient-corrected exchange-correlation functionals. *Theor. Chem. Acc.* **2005**, 114, 145-152.

27. Perdew, J. P., Burke, K., Ernzerhoff, M. Generalized Gradient Approximation Made Simple. *Phys. Rev. Lett.* **1996**, 77, 3865.
28. Grimme, S., Antony, J., Ehrlich, S., Krieg, H. A consistent and accurate *ab initio* parametrization of density functional dispersion correction (DFT-D) for the 94 elements H-Pu. *J. Chem. Phys.* **2010**, 132, 154104.
29. Grimme, S., Ehrlich, S., Goerigk, L. Effect of the damping function in dispersion corrected density functional theory. *J. Comput. Chem.* **2011**, 32, 1456-1465.
30. Larsen, A. H., Mortenson, J. J., Blomqvist, J., Castelli, I. E., Christensen, R., Dułak, M., Friis, J., Groves, M. N., Hammer, B. & Hargus, C. The atomic simulation environment – a Python library for working with atoms. *J. Phys.: Condens. Matter* **2017**, 29, 273002.
